# Structural snapshots along K48-linked ubiquitin chain formation by the HECT E3 UBR5

**DOI:** 10.1038/s41589-023-01414-2

**Published:** 2023-08-24

**Authors:** Laura A. Hehl, Daniel Horn-Ghetko, J. Rajan Prabu, Ronnald Vollrath, D. Tung Vu, David A. Pérez Berrocal, Monique P. C. Mulder, Gerbrand J. van der Heden van Noort, Brenda A. Schulman

**Affiliations:** 1 Department of Chemistry, School of Natural Sciences, Technical University of Munich, Garching, Germany; 2 Department of Molecular Machines and Signaling, Max Planck Institute of Biochemistry, Martinsried, Germany; 3 Department of Proteomics and Signal Transduction, Max Planck Institute of Biochemistry, Martinsried, Germany; 4 Department of Cell and Chemical Biology, Leiden University Medical Centre, Leiden, the Netherlands

**Keywords:** Structural biology, Enzyme mechanisms, Post-translational modifications

## Abstract

Ubiquitin (Ub) chain formation by homologous to E6AP C-terminus (HECT)-family E3 ligases regulates vast biology, yet the structural mechanisms remain unknown. We used chemistry and cryo‐electron microscopy (cryo‐EM) to visualize stable mimics of the intermediates along K48-linked Ub chain formation by the human E3, UBR5. The structural data reveal a ≈ 620 kDa UBR5 dimer as the functional unit, comprising a scaffold with flexibly tethered Ub-associated (UBA) domains, and elaborately arranged HECT domains. Chains are forged by a UBA domain capturing an acceptor Ub, with its K48 lured into the active site by numerous interactions between the acceptor Ub, manifold UBR5 elements and the donor Ub. The cryo-EM reconstructions allow defining conserved HECT domain conformations catalyzing Ub transfer from E2 to E3 and from E3. Our data show how a full-length E3, ubiquitins to be adjoined, E2 and intermediary products guide a feed-forward HECT domain conformational cycle establishing a highly efficient, broadly targeting, K48-linked Ub chain forging machine.

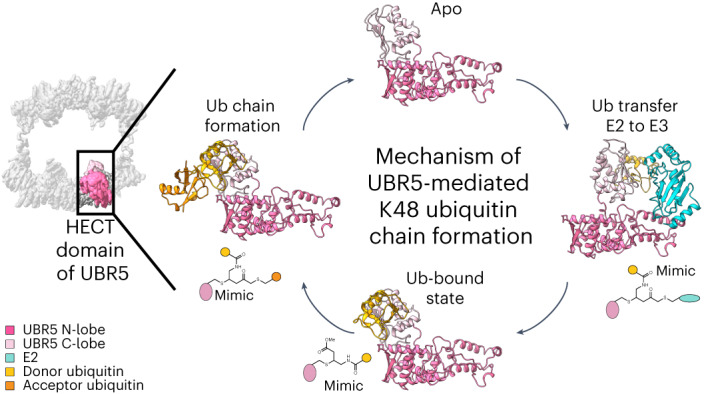

## Abstract (web summary)

Homologous to E6AP C-terminus (HECT) E3s forge polyubiquitin chains through multiple reaction steps. A HECT polyubiquitylation cascade was visualized step-by-step, through use of chemical tools and cryo‐EM, and revealed how K48 linkage-specificity is attained by oligomeric UBR5.

## Main

The ubiquitin (Ub) code depends on specific types of poly-Ub chains determining fates of modified proteins^1,2^. For example, widespread regulation is achieved by K48-linked Ub chains triggering proteasomal degradation, while chains linked between Ub’s N-terminus or K63 are associated with signaling and membrane protein trafficking^3–7^. Branched chains, where one type of Ub chain is further modified by K48-linkages, are particularly potent at eliciting protein turnover^8–13^. Given the fundamental roles of poly-Ub chains in biological regulation, and of K48-linked chains in particular, it is important to understand the structural mechanisms by which these linkages are forged.

Polyubiquitylation is achieved by E2 and E3 enzymes collaborating to link the C-terminus of a ‘donor Ub’ (Ub^D^) to a specific site on an ‘acceptor Ub’ (Ub^A^). The underlying mechanism depends on the type of E3 ligase catalytic domain^14–16^. For many E3s, really interesting new gene (RING) domains bind and activate Ub^D^ transfer from the catalytic Cys of an E2 to a recruited Ub^A^. As such, RING E3 linkage-specificity is typically determined by the E2 partner^17^. On the other hand, E3s with Homologous to E6AP C-termins (HECT) and RING-between-RING (RBR) catalytic domains directly mediate Ub^D^ transfer and determine the type of poly-Ub chain produced^6,18–20^. HECT- and RBR-family E3s have active site cysteines that generate Ub chains through two reaction steps^21–23^. The transition state 1 (TS1) directs transfer of Ub^D^’s C-terminus from an E2’s catalytic Cys to that of the E3, producing a reactive, thioester-bonded E3~Ub^D^ intermediate. The transition state 2 (TS2) directs Ub^D^ transfer from the E3 to Ub^A^. Structures of RING and RBR complexes, chemically trapped in action, have shown how these E3 families form Ub chains with various linkages^24–29^. However, structural knowledge for Ub chain formation by HECT E3s is lacking.

HECT E3s were the first family of Ub ligases discovered^21,22^. The nearly 30 human HECT E3s regulate numerous biological processes^19,30^. Their distinct regulatory functions are determined by unique regions N-terminal of the catalytic HECT domain^22^. HECT domains have the following bilobal structure: a larger N-terminal ‘N-lobe’ binds E2, and a smaller C-terminal ‘C-lobe’ harbors the catalytic Cys^31,32^. Two frequently observed N-lobe/C-lobe configurations are ‘L’ and ‘Inverted T’, named for the shape with the N-lobe on the bottom. These differ by rotation of ≈150° about the flexible interlobe linker, placing the C-lobe either to one side (L) or toward the middle of the N-lobe (Inverted T). Prior crystal structures have represented HECT domains in the Inverted T conformation receiving Ub^D^ from E2 during the TS1 reaction, in both conformations covalently linked to a Ub^D^, and in the L-conformation transferring Ub^D^ to a substrate^33–36^. Also, a comprehensive mutagenesis study suggested NEDD4-family HECT E3s build K63-linked Ub chains in the L-conformation^34^. Nonetheless, in the decade since this report, no further structures have been published that provide representation of any state during HECT E3-catalyzed ubiquitylation more than once, nor of any Ub transfer intermediate for a full-length HECT E3. The suite of intermediates has not been visualized for any HECT E3. Thus, whether HECT E3s transfer Ub through conserved catalytic architectures is unclear. Furthermore, the functional relevance of different N-lobe/C-lobe arrangements remains the subject of debate^32,37^.

A HECT ligase of emerging importance is the 2799-residue, multidomain, human UBR5 (ref. ^30,38–46^). UBR5 specifically generates K48-linked Ub chains, including by branching preformed K11- or K63-linked chains^9,11^. UBR5 has key roles in stem cell pluripotency, tumor suppression, oncogenesis and other important pathways^40–42,46^. Prior structure–function studies showed UBR5’s Ub-associated (UBA) domain binds Ub and suggested a role for this interaction during polyubiquitylation^47–52^. However, without structural data showing the transition states, knowledge of how the domains would be arranged across the cascade where Ub^D^ is transferred from an E2 to the HECT domain catalytic Cys and then to Ub^A^ remains rudimentary.

Here we address this problem with a suite of cryo‐electron microscopy (cryo‐EM) reconstructions for chemically stable proxies for the TS1, UBR5~Ub^D^ and TS2 intermediates. Comparison to prior structures identifies a conserved HECT domain conformational trajectory for Ub^D^ transfer from E2 to E3 to a target. The conformations and structural transitions along the polyubiquitylation cascade are achieved synergistically by elements of UBR5’s N-terminal regions, the HECT domain, the E2~Ub^D^ intermediate and the donor and acceptor Ubs. Furthermore, the structure of the TS2 intermediate shows an intricate web of interactions placing Ub^A^’s K48 into the ubiquitylation active site. The data reveal a HECT E3 linkage-specific polyubiquitylation cascade, for K48 chains forged by UBR5.

## Results

### UBR5 assembly and elements forging K48-linked chains

**Fig. 1:**
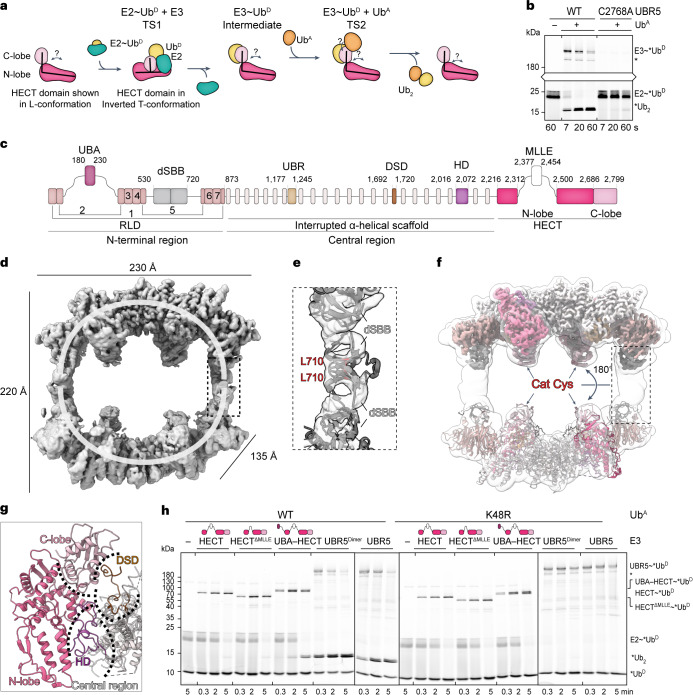
Cryo-EM of UBR5 reveals oligomeric scaffold elaborately arranging HECT domains. **a**, Cartoon depicting HECT E3-mediated Ub chain formation. **b**, Di-Ub synthesis assay testing activity of UBR5 WT and C2768A mutant. Fluorescently labeled K48R Ub^D^ (*Ub^D^) is tracked through the cascade. The resulting di-Ub product is labeled *Ub_2_. Only upper and lower portions of the nonreducing gels showing *Ub^D^-linked moieties are illustrated and connected for clarity. Throughout this work, single asterisk marks a UBR5-degradation-product that remains catalytically active. The assay was performed five times with similar results. **c**, UBR5 domains based on cryo-EM structure. **d**, Cryo-EM map of UBR5^C2768A^ highlighting the two U-shaped units. Dotted box indicates dSBB domains shown in **e** as mediating tetramerization between the two dimeric units. **e**, AlphaFold2 model of dSBB domains with L710, in red. **f**, Transparent low-pass filtered map of tetrameric UBR5^C2768A^ superimposed on UBR5^Dimer^ density (top half) and experimentally derived coordinates (lower half). Rotation of 180° across the two halves is indicated. Dotted box indicates the position of dSBB domains, not well-resolved in the UBR5^Dimer^ density. **g**, Close-up showing DSD and HD domain interactions with HECT domain C-lobe and N-lobe, respectively. **h**, Di-Ub synthesis assay testing various versions of UBR5:structurally redefined HECT domain with or without (HECT^ΔMLLE^) MLLE insertion, with the UBA domain connected by a 15-residue linker, dimeric or WT full-length UBR5. Linkage-specificity was confirmed by comparing WT Ub^A^ or K48R Ub^A^. The assay was performed twice with similar results. Source data

**Extended Data Fig. 1:**
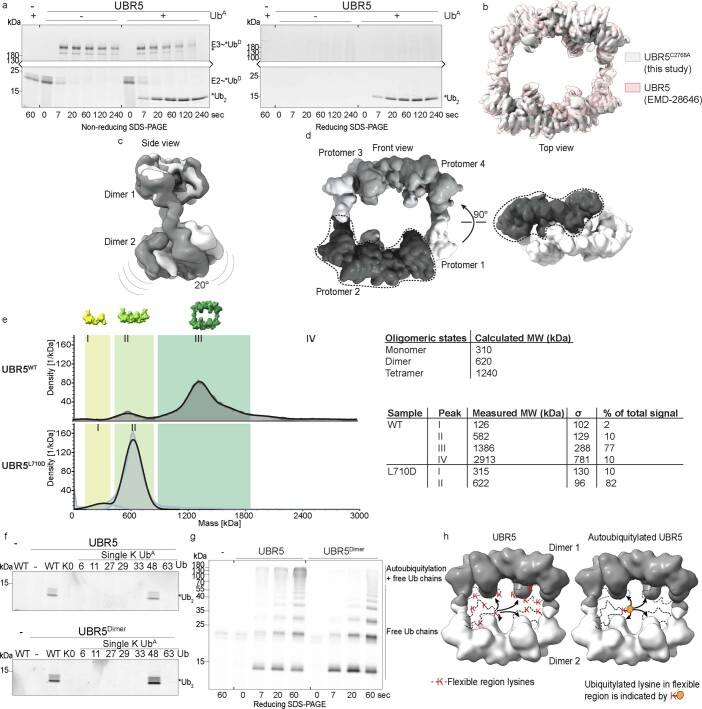
Functional characterization of UBR5 and UBR5^Dimer^. **a**, Autoubiquitylation assay of UBR5. E2~*Ub^D^ was added to UBR5 or UBR5 with Ub^A^. SDS-PAGE shows isopeptide-linked Ub^D^-bound products (reducing) or additionally thioester-bonded products (nonreducing). Two replicates were performed with similar results. **b**, Overlay of UBR5^WT^ map (EMD-28646) with UBR5^C2768A^ (this study). **c**, Breathing motion of UBR5^C2768A^ during 3D classification. **d**, Monomers within tetramer are shaded individually, one is further highlighted using a dotted line. **e**, Molecular weights of UBR5^WT^ and UBR5^L710D^ assemblies determined by mass photometry. Measured and calculated MWs for distinct oligomeric states are shown. Measurements in triplicates gave comparable graphs. **f**, Di-Ub synthesis assay testing linkage-specificity of UBR5^WT^ and UBR5^Dimer^. Ub^A^ with all lysines (WT), all lysines mutated to arginine (K0) or all but one Lys mutated to Arg, was used. Triplicates showed similar results. **g**, Free Ub chain formation in pulse-chase format for UBR5 and UBR5^Dimer^. Independent triplicates showed similar results. **h**, Low-pass filtered map of tetrameric UBR5 with dimers shaded. Left, unmodified UBR5 with flexible regions indicated by a dotted line, containing several lysines. Reachability of different HECT domains in cis or trans indicated by arrows. Right, autoubiquitylated UBR5. Accessibility of HECT domains in cis or trans for this Ub to serve as Ub^A^ is indicated. Source data

Enzymatic cascades for HECT E3s can be examined by pulse-chase assays monitoring Ub transfer, starting with an E2~Ub^D^ intermediate (here with UBE2D) generated in the pulse reaction^34^ (Fig. 1a). Fluorescent labeling allows detection of Ub^D^; a K48R variant prevents its use as acceptor. In the chase reaction, E2~*Ub^D^ is added to UBR5 and unlabeled Ub^A^. Tracking fluorescent Ub^D^ along transfer from E2 to E3 to Ub^A^ based on electrophoretic mobility in SDS–PAGE confirmed that wild-type (WT) UBR5 displays catalytic C2768-dependent Ub chain-forming activity and can forge free Ub chains (Fig. 1b and Extended Data Fig. 1a).

Cryo-EM data for catalytically inactive UBR5^C2768A^ showed a giant ≈230 Å × 220 Å × 135 Å ovoid multidomain assembly of two U-shaped units (Fig. 1c,d, Supplementary Fig. 1 and Supplementary Table 1; note the C2768A mutant was used because we produced this in large-scale before WT). Three-dimensional (3D) classifications and fitting models of domains generated by AlphaFold2 into a map refined at 3.7 Å resolution agree with cryo-EM data for WT UBR5 published during revision of our manuscript^51,52^ and revealed three key structural properties (Extended Data Fig. 1b,c). First, each U-shaped assembly seemed a fundamental structural unit, varying in angles relative to each other by up to ≈20° in different classes. Second, each U-shaped unit corresponds to a dimer of UBR5 protomers (Fig. 1). Two HECT domains project toward the center of each U-shaped dimer from opposite directions. Finally, there are two dimerization interfaces. Extensive interactions rigidly affix two protomers at the center of the U (Extended Data Fig. 1d). Interactions between double small β-barrel (dSBB) domains more loosely connect two dimers at the tops of the ‘U’s. Indeed, an L710D variant at the dSBB interface prevented tetramerization and led to the formation of a catalytically active dimer (Fig. 1e and Extended Data Fig. 1e–h).

**Extended Data Fig. 2:**
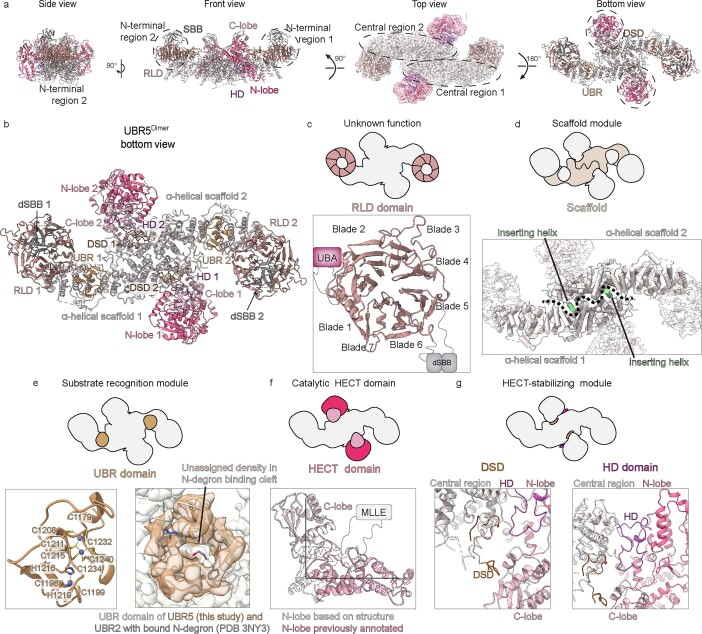
Structural characterization of UBR5^Dimer^. **a**, Views of UBR5^Dimer^ structure, termed ‘side’, ‘top’, ‘front’ and ‘bottom’, used in subsequent figures. **b**, Bottom-view of UBR5^Dimer^ showing domains of protomers 1 and 2. **c**, Structure of 7-bladed β-propeller (RLD). First blade starts at UBR5’s N-terminus, and is completed by two more C-terminal β-strands. The second blade has three antiparallel strands and a ~270 residue insertion including the UBA domain (plum, not visible in UBR5^Dimer^ map). The remaining blades are conventional, except for a >220-residue insertion containing dSBB domains, between the fifth and sixth blade. **d**, Analysis of scaffold module. α-helical bundles in protomers form intertwined dimerization-interface with one helix (1912-1928) particularly inserted into the other protomer. **e**, Left, structural analysis of a substrate recognition module: UBR domain is positioned within the scaffold, facing the HECT domain in trans. C1196/C1199/H1216/H1219, C1215/C1234/C1240/C1211, and C1211/C1179/C1208/C1232 coordinate three zinc ions. Right, cryo-EM map over UBR domain shows unassigned density in the canonical peptide-binding cleft indicated by overlay with crystal structure of UBR2s UBR domain bound to a substrate peptide (PDB: 3NY3). **f**, L-shaped HECT domain (white) compared with the previously annotated domain (N-lobe in pink, C-lobe in light pink). **g**, Structural analysis of HECT-stabilizing module. Left, zoom into DSD domain (brown) meandering between scaffold and HECT domain C-lobe. Right, zoom-in on HD domain (purple), interacting with the central region and the HECT domain N-lobe and contacting the interlobe linker connecting N- and C-lobe.

Cryo-EM data for the L710D variant, hereafter termed UBR5^Dimer^, yielded a map allowing the building and refining of a twofold symmetric experimentally derived structure at 2.7 Å resolution (Fig. 1f and Supplementary Fig. 2). The structure showed most domains within each protomer (Extended Data Fig. 2a–g). The N-terminal region consists of an interrupted RCC1-like domain (RLD) β-propeller, with UBA (not visible in the map) and dSBB domains inserted in blades 2 and 5, respectively. The central region is primarily α-helical but also embeds the UBR domain that has been proposed to bind substrate N-termini^48,53,54^. Interestingly, the putative substrate-binding cleft of UBR5’s UBR domain was occupied by additional density, although this could not be assigned. UBR5’s C-terminal HECT domain initiates with residues not originally annotated as being part of this domain and adopts the L-conformation. The HECT domain N-lobe is interrupted by insertion of the MLLE domain^49,55,56^, which has been implicated in substrate binding but is not visible in any of our cryo-EM maps. As in most crystal structures of isolated HECT domains, UBR5’s six C-terminal ‘tail’ residues are not visible and are presumably flexible^32^.

The HECT domain L-configuration is stabilized by numerous interactions. Two meandering sequences emanating from the scaffold—which we term domain swap dimerization (DSD, residues 1691–1720) and HECT display (HD, residues 2016–2076) domains—extensively bind the HECT domain. One protomer’s DSD domain contributes to the extensive dimerization interface within the scaffold, while a peptide-like loop projects from the scaffold to bind the C-lobe from one HECT domain. Meanwhile, one side of the HD domain interacts with the α-helical portion of the scaffold, and the other side nestles in a concave surface of the HECT domain N-lobe opposite the E2-binding site. Because the residues leading to and from the HD domain are not visible in the EM density, this domain could in principle rotate to display the HECT domain N-lobe in various orientations. Numerous additional interactions further position UBR5’s HECT domain (Fig. 1g), including between the central region and HECT domain N-terminus, and between the HECT domain N- and C-lobes. The elaborate nature of the assembly portended an important role of this specific HECT domain architecture in UBR5-mediated polyubiquitylation.

Some isolated HECT domains can mediate Ub chain formation, although less efficiently than full-length E3s^19,31,32,57^. We thus compared the activities of UBR5, UBR5^Dimer^, and the structurally redefined HECT domain (with or without the MLLE domain insertion). The truncated versions produced only little di-Ub (Fig. 1h). We considered that the UBA domain could have a key role, because other HECT E3s, as well as other types of Ub chain-forming enzymes, depend on Ub-binding domains to recruit an acceptor Ub^24–28,57,58^. A minimal version of UBR5 with the UBA and HECT domains connected by a 15-residue linker did generate di-Ub chains, albeit less efficiently than UBR5 and UBR5^Dimer^. This agrees with catalytic activities reported for other HECT E3s and suggests potentially similar chain-forming mechanisms^57^. Notably, both the UBA–HECT fusion and UBR5^Dimer^ retained specificity for forming K48-linked Ub chains (Fig. 1h and Extended Data Fig. 1f). WT UBR5 and UBR5^Dimer^ showed subtle differences in autoubiquitylation. It seems likely that the autoubiquitylation sites, and their linked Ubs, have additional opportunities to access the catalytic centers in the tetramer, not only in *cis* within a UBR5 protomer and in *trans* within the dimer but also two more geometries in *trans* (Extended Data Fig. 1g,h).

Overall, the data showed that the UBR5 UBA and HECT domains have critical roles in K48-linked Ub chain formation and that its high level of activity makes UBR5^Dimer^ suitable for structurally defining the Ub chain forming cascade.

### Visualizing TS1 assembly: Ub^D^ transfer from E2 to UBR5

**Fig. 2:**
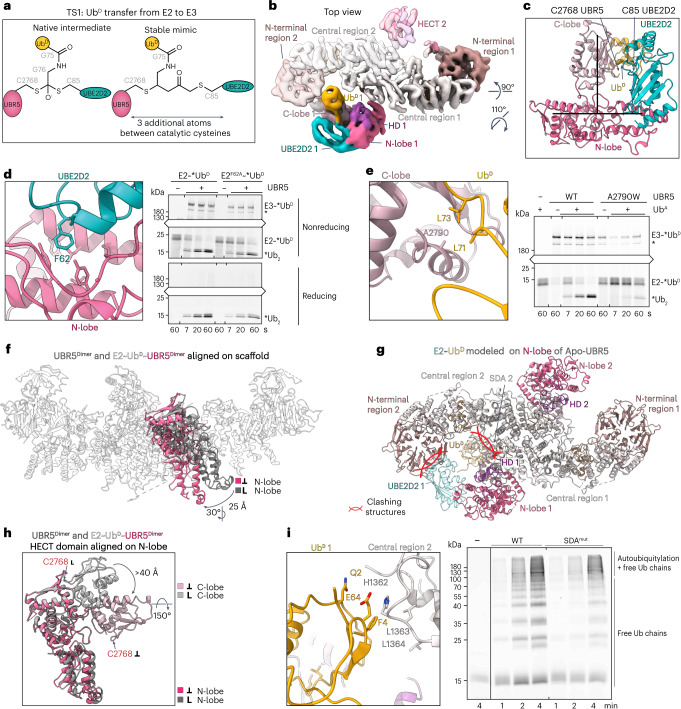
Cryo-EM map visualizing Ub^D^ transfer from E2 to UBR5. **a**, Chemical structures of native TS1 and stable mimic for Ub^D^ transfer from E2 to UBR5. E2-to-E3 catalytic Cys distance is increased by three atoms in stable mimic compared to native TS1. **b**, Cryo-EM map of UBE2D2~Ub^D^~UBR5^Dimer^. **c**, Model of E2~Ub^D^ (PDB: 3JVZ)-bound UBR5 HECT domain. Catalytic Cys of UBE2D2 (C85) and UBR5 (C2768) are labeled. **d**, Left, close-up of E2~Ub^D^~UBR5^Dimer^ model over E2-UBR5 interface. Right, di-Ub synthesis assay testing this interface using E2 F62A variant (UBE2D3 used). Three replicates showed similar results. A catalytically active UBR5-degradation-product is marked with an asterisk in **d** and **e.** **e**, Left, close-up of E2~Ub^D^~UBR5^Dimer^ model over C-lobe-Ub^D^-interface. Right, di-Ub synthesis assay to test interface by steric clash (A2790W). Three replicates showed similar results. **f**, Superposition of apo-UBR5 or E2~Ub^D^~UBR5^Dimer^ aligned on scaffold N-lobes in gray and pink. UBR5 is depicted up to residue 2686 for clarity. HECT conformations are indicated with colored boxes and symbols (representation also used in subsequent figures). **g**, Docking E2~Ub^D^ on apo-UBR5 shows clashing in this conformation, indicated with intersecting red arcs. **h**, Rotation of the C-lobe between apo-UBR5 (gray) and TS1 (pink), aligned on N-lobes. **i**, Left, close-up of UBR5 SDA loop and Ub^D^’s F4 patch in E2~Ub^D^~UBR5^Dimer^ model. Right, polyubiquitylation assay testing activity of SDA mutant (HLL1362-1364DDD). Three replicates showed similar results. Source data

**Extended Data Fig. 3:**
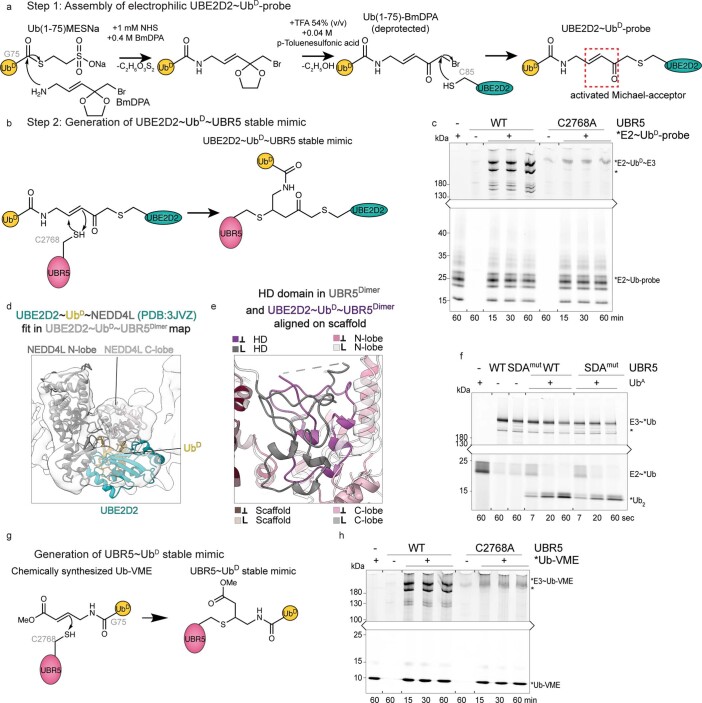
Chemical biology tools to visualize Ub^D^ transfer from E2 to UBR5 and subsequent UBR5~Ub^D^ intermediate. **a**, Stable mimic representing Ub^D^ transfer from E2 to UBR5 (TS1). Reaction scheme, first step: installation of an electrophile between Ub^D^ C-terminus (Ub^D^’s C-terminal G76 is deleted to approach near-native geometry) and active site Cys of E2 UBE2D2 (other cysteines mutated, see Methods). **b**, Reaction scheme, second step: the UBE2D2~Ub^D^ probe reacted with UBR5^Dimer^ generating UBE2D2~Ub^D^~UBR5^Dimer^ with E2, Ub^D^, and UBR5^Dimer^ linked at a single atom. **c**, Dependence for UBE2D2~Ub^D^~UBR5^Dimer^ complex formation on UBR5s catalytic Cys was tested using fluorescent UBE2D2 in the probe. Two replicates showed similar results. **d**, Crystal structure of UBE2D2~Ub^D^~HECT^NEDD4L^ (PDB: 3JVZ), containing E2~Ub^D^ oxyester linkage with native distances fit into UBE2D2~Ub^D^~UBR5^Dimer^ map. **e**, Close-up of HD domain in apo UBR5^Dimer^ and UBE2D2~Ub^D^~UBR5^Dimer^ (grey and purple), aligned on scaffold. HECT domain conformations are indicated. **f**, Di-Ub-synthesis assay for the SDA-mutant. Triplicates of this assay showed comparable results. **g**, Reaction scheme to generate stable mimic of UBR5~Ub^D^. Fully synthetic Ub-VME was reacted with UBR5^Dimer^. **h**, UBR5 catalytic Cys dependence for forming UBR5~Ub^D^ complex was tested using a fluorescent version of Ub-VME. The specificity was tested twice. Source data

We sought structural insights into the TS1 catalytic assembly, which mediates transfer of Ub^D^ from the E2’s active site Cys to the E3’s active site Cys (Fig. 2a). A stable mimic of this fleeting reaction was generated with the E2 UBE2D, by adapting our method previously applied to an RBR E3 (ref. ^59^). An electrophile installed at the active site of a proxy for the UBE2D~Ub^D^ intermediate reacted with UBR5^Dimer^, dependent on the catalytic Cys (Extended Data Fig. 3a–c). Cryo-EM of the E2~Ub^D^~UBR5^Dimer^ complex showed considerable heterogeneity (Supplementary Fig. 3). Nonetheless, a map refined without symmetry at 7.3 Å resolution showed density for one E2~Ub^D^~HECT domain assembly, which was readily fit with the prior crystal structure^33^ of NEDD4L’s HECT domain bound to an oxyester-linked mimic of UBE2D~Ub^D^ (Fig. 2b and Extended Data Fig. 3d). Because the latter complex maintained the geometry of the native ubiquitylation intermediate, its structural superposition supports suitability of our chemical proxy despite three additional atoms between E2 and E3 catalytic cysteines. A model of TS1 for UBR5 was generated by combining the docked E2~Ub^D^ moiety with three portions of the UBR5^Dimer^ cryo-EM structure (the scaffold, HD domain with HECT domain N-lobe, and HECT domain C-lobe; Fig. 2c). The model shows Ub^D^’s C-terminus and the active site cysteines of UBE2D and UBR5^Dimer^ juxtaposed as expected for the TS1 intermediate. Notably, in this state, the HECT domain N- and C-lobes adopt the Inverted T-conformation, placing the E2 and E3 active sites for Ub^D^ transfer between them. This conformation is stabilized by avid interactions between the HECT domain and the E2~Ub^D^ intermediate. Accordingly, reducing N-lobe contacts with a UBE2D F62A variant^60^, or introducing a steric clash in the C-lobe-Ub^D^ interface (A2790W)^33^, impaired E3 ligase activity (Fig. 2d,e).

While the scaffold itself superimposes between the apo and E2~Ub^D^-bound UBR5 assemblies, there are substantial differences in the relative orientations of the HECT domain N- and C-lobes. First, the N-lobe tilts by 25° relative to the scaffold (Fig. 2f). This rotation is required because the N-lobe position in apo-UBR5 is incompatible with E2~Ub^D^ binding—a bound E2 would clash with the RLD from the opposite protomer in the dimer, and its linked Ub^D^ would clash with the scaffold from one protomer and a loop we term ‘scaffold donor Ub approaching’ (SDA, residues H1362–L1364) from the other (Fig. 2g). At this resolution, we cannot unambiguously determine if UBR5’s N-lobe remains bound to the HD domain in the TS1 intermediate, although the necessary rotation could be achieved by eliminating some of its contacts to the scaffold (Extended Data Fig. 3e). Second, to face the N-lobe-bound E2, the C-lobe has rotated ≈150° about the interlobe tether (Fig. 2h). This shifts the position of the E3 catalytic Cys by >40 Å. This positioning of the C-lobe grants UBR5’s catalytic Cys access to the E2~Ub^D^ active site but would require its disengagement from the DSD domain. Thus, it seems that E2~Ub^D^ binding not only directs the catalytic architecture of the HECT domain but also orchestrates substantial rearrangement in the context of the UBR5 scaffold.

Finally, in the E2~Ub^D^~UBR5 assembly, the Ub^D^ F4 patch is poised to graze the SDA loop. We tested the function of the SDA loop by replacing the sequence with aspartates. The SDA mutation did not overtly affect Ub^D^ transfer from E2 to UBR5, nor to Ub^A^ in pulse-chase assays monitoring di-Ub synthesis (Extended Data Fig. 3f). However, it caused a subtle but obvious defect in forming low-molecular-weight poly-Ub chains in assays with multiple cycles of E1–E2–UBR5 activities (Fig. 2i).

### Cryo-EM of a stable proxy of UBR5~Ub^D^ intermediate

**Fig. 3:**
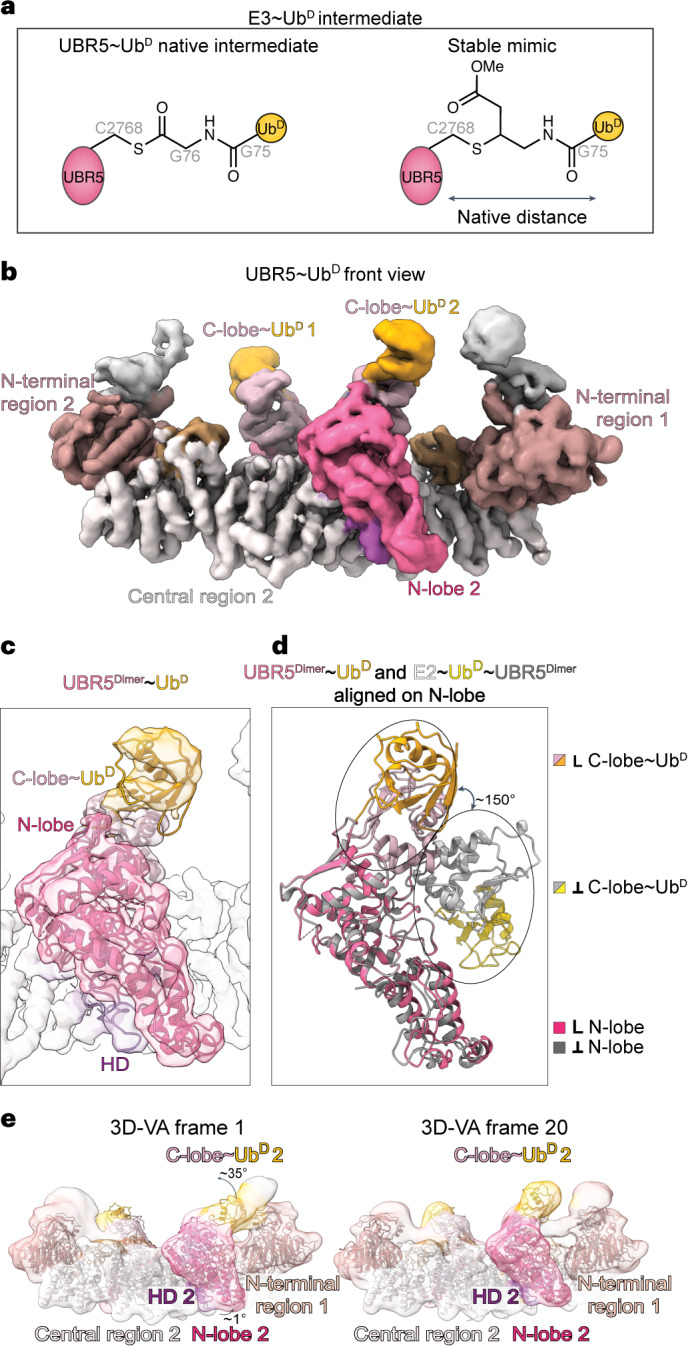
Cryo-EM of stable mimic of UBR5^Dimer^~Ub^D^ intermediate. **a**, Chemical structures of TS1 reaction product UBR5~Ub^D^ and chemically stable mimic. The stable mimic using Ub-VME to couple Ub^D^ to UBR5^Dimer^’s catalytic Cys maintains native number of atoms between the E3 Cys and Ub^D^ G75. **b**, Cryo-EM map of stable mimic representing UBR5^Dimer^~Ub^D^. **c**, Model of UBR5^Dimer^~Ub^D^ from fitting coordinates for UBR5^Dimer^ and Ub^D^ into the density. **d**, Overlay of HECT domain from UBR5^Dimer^~Ub^D^ (L-conformation) in pink/orange and from E2~Ub^D^~UBR5^Dimer^ (Inverted T-conformation) in gray/yellow indicates conformational change after Ub^D^ transfer from E2 to UBR5. For clarity, only the UBR5 HECT domain and Ub^D^ are shown. **e**, Left, frame 1 and right, frame 20 of 3D-VA performed on UBR5~Ub^D^. UBR5^Dimer^ N-terminal and central region, HECT domain N-lobe and C-lobe, and Ub^D^ were individually fitted into density.

After its transfer from the E2, Ub^D^ is thioester-bonded to UBR5’s catalytic Cys (Fig. 3a). We generated a stable proxy for this intermediate by mixing Ub-vinyl methyl ester (Ub-VME)^61^ with UBR5^Dimer^, which reacted depending on the catalytic Cys (Fig. 3a and Extended Data Fig. 3g,h). Cryo-EM of the resultant UBR5^Dimer^~Ub^D^ complex yielded a map at 5.3 Å resolution (Fig. 3b,c and Supplementary Fig. 4). The map superimposed with the structure of apo-UBR5^Dimer^, with additional density for Ub^D^ adjacent to both C-lobes. We generated a structural model by docking (1) Ub^D^, (2) the UBR5^Dimer^ structure through the HECT domain N-lobe, and (3) the C-lobe and DSD domain.

Comparing the cryo-EM-based models for E2~Ub^D^~UBR5^Dimer^ and UBR5^Dimer^~Ub^D^ showed common interactions between Ub^D^ and the HECT domain C-lobe. However, in the UBR5^Dimer^~Ub^D^ complex, the scaffold and HECT domain reverted to a configuration like apo-UBR5 (Fig. 3d). The structures suggest that after Ub^D^ transfer from E2 to UBR5 with the HECT domain in the Inverted T-configuration, the C-lobe and its linked Ub^D^ turn around and face the opposite direction, in the L-orientation. To gain insights into the structural heterogeneity of the UBR5^Dimer^~Ub^D^ complex, we applied 3D variability analysis (3D-VA)^62^. This revealed a spectrum of orientations for the Ub^D^-linked C-lobe (Fig. 3e and Supplementary Video 1). One extreme is intermediary between the HECT domain Inverted T- and L-conformations. The other extreme is the final L-orientation. This is consistent with a conformational progression whereby the C-lobe and its covalently linked Ub^D^ rotate about the linker to the N-lobe.

Because the UBR5^Dimer^~Ub^D^ model showed the scaffold and HECT domain N-lobe oriented as in the UBR5^Dimer^ structure, we speculate that after Ub^D^’s C-terminal linkage is transferred to UBR5, N-lobe reorientation would be enabled by E2 dissociation. Elimination of constraints from E2 clashing with UBR5’s N-terminal region presumably facilitates the relocation of the N-lobe, which we speculate then facilitates redirection of the Ub^D^-bound C-lobe into the L-configuration.

### Cryo-EM of UBR5 forging a K48-linked Ub chain

**Fig. 4:**
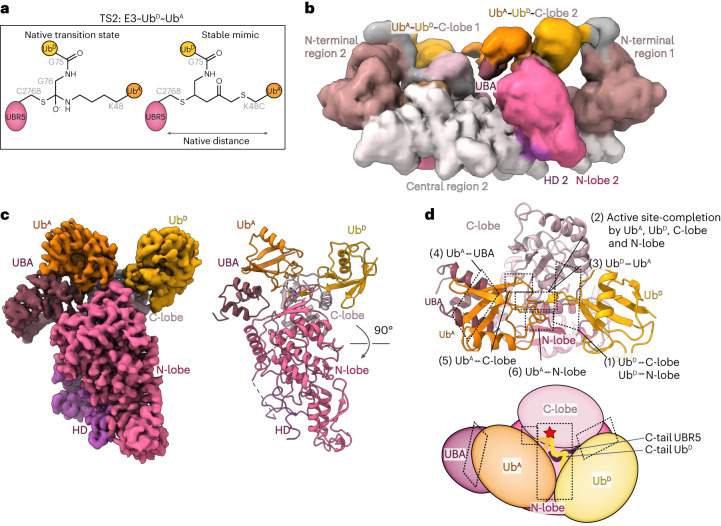
Cryo-EM structure visualizing HECT E3-mediated linkage-specific Ub chain formation. **a**, Chemical structures of native TS2 for Ub chain formation and chemically stable mimic. TS2 is mimicked by the installation of electrophilic moiety between the C-terminus of Ub (truncated at G75, representing Ub^D^, yellow) and a Cys replacement for acceptor Ub’s K48 (Ub^A^, orange). This traps UBR5^Dimer^’s catalytic Cys via a stable three-way cross-link while maintaining the native number of atoms between E3 catalytic Cys, G75 in Ub^D^ and the α-carbon for residue 48 in Ub^A^. **b**, Initial low-resolution cryo-EM map of stable mimic of the UBR5^Dimer^~Ub^D^~Ub^A^ complex (TS2). **c**, Local refined map (left) and atomic model (right) for catalytic complex mediating K48-linked Ub chain formation, wherein a UBA domain recruits Ub^A^ and residue 48 is placed at the HECT~Ub^D^ active site. For clarity, the remaining density corresponding to the scaffold is hidden. **d**, Structure above and cartoon below, indicating interfaces establishing the catalytic geometry for K48-linked Ub chain formation.

**Extended Data Fig. 4:**
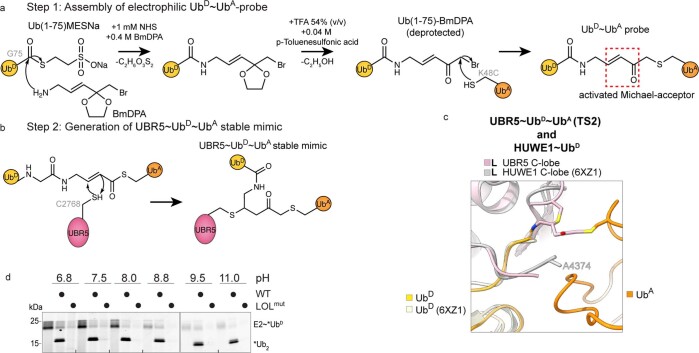
Chemical biology tools to visualize K48-linked Ub chain formation by UBR5. **a**, Stable mimic representing TS2, Ub^D^ transfer from UBR5 to Ub^A^, was generated in two steps. Reaction scheme for the first step: installation of electrophile between Ub^D^ C-terminus (here, Ub^D^’s C-terminal G76 is deleted to obtain native distances) and a Cys replacement for K48 in Ub^A^. **b**, Reaction scheme for second step to generate TS2 mimic: the Ub^D^~Ub^A^-probe was reacted with UBR5^Dimer^ to generate a UBR5^Dimer^~Ub^D^~Ub^A^ complex wherein the C-terminus of Ub^D^, residue 48 of Ub^A^, and UBR5’s catalytic Cys are linked at a single atom. **c**, Structural superposition of UBR5’s C-lobe bound to Ub^D^ and Ub^A^ in TS2 and HUWE1’s C-lobe bound to Ub^D^ (PDB: 6XZ1). The C-terminus of HUWE1 overlays with UBR5’s C-terminus in TS2. The ultimate residue was resolved for HUWE1 and provides a lead to UBR5’s ultimate residue. **d**, Di-Ub synthesis assay testing pH-dependency of LOL-caused deficiency. E2~*Ub^D^ was added to WT or LOL-mutant UBR5 at indicated pH. This experiment was performed twice with comparable results. Source data

K48-linked chains are formed through the transfer of Ub^D^’s C-terminus from UBR5’s catalytic Cys to K48 on Ub^A^ (Fig. 1a). To visualize the catalytic assembly, we adapted a method previously used to study deubiquitylating and RBR E3 enzymes^14,59^. Precise catalytic geometry is required for HECT E3-mediated polyubiquitylation^63^. Thus, a stable mimic of the transition state (TS2) maintaining native distances between α-carbons of key residues was generated (Fig. 4a) achieved by an electrophile installed between the C-terminus of a truncated Ub^D^ and a Cys replacement for K48 on Ub^A^ that was reacted with UBR5^Dimer^ (Extended Data Fig. 4a,b). An 8.3 Å resolution cryo-EM map of the resultant UBR5^Dimer^~Ub^D^~Ub^A^ complex largely superimposed with the structure of UBR5^Dimer^~Ub^D^ (Fig. 4b and Supplementary Fig. 5). The HECT domain C-lobe binds Ub^D^ much like in the cryo-EM maps for the E2~Ub^D^~UBR5^Dimer^ and UBR5^Dimer^~Ub^D^ complexes. Strikingly, the maps also showed clear density corresponding to Ub^A^.

Local refinement yielded a 3.3 Å resolution map resolving elements defining the configuration for polyubiquitylation (Fig. 4c,d). The interactions stabilizing the HECT domain L-conformation are maintained as in apo-UBR5^Dimer^. In addition to Ub^D^ and Ub^A^, the map also shows the UBA domain and additional density around the active site that was not visible in the other maps. The UBA domain binds Ub^A^ positioned with its residue 48 at the active site. With the HECT domain in the L-conformation, the Ub^D^-linked catalytic Cys is situated not only adjacent to the acceptor but also at the junction with the N-lobe, which is thus also poised to contribute to Ub chain formation.

**Fig. 5:**
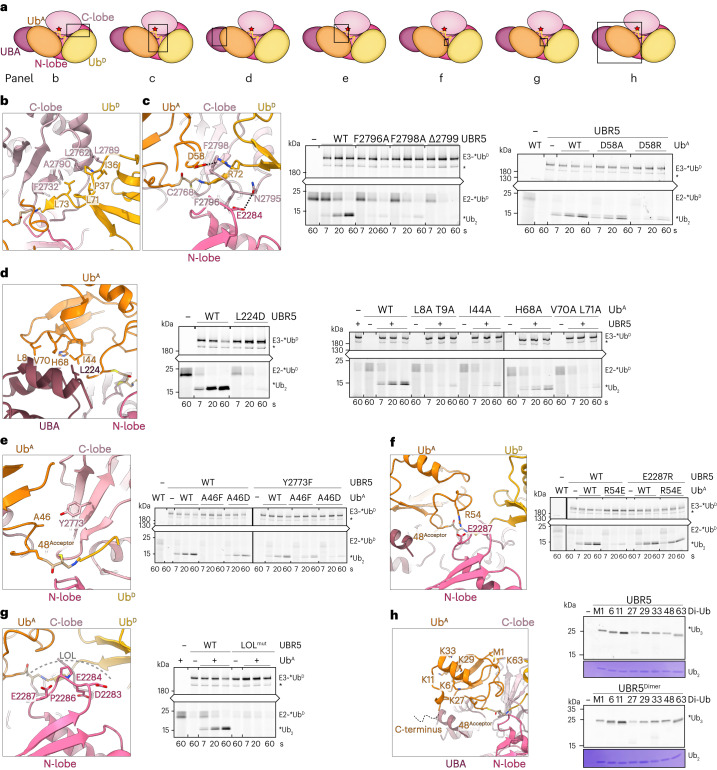
Elaborate interactions in HECT E3-mediated K48-linked Ub chain formation. **a**, Cartoon of polyubiquitylation complex, highlighting regions tested in **b**–**h**. **b**, Left, UBR5 C-lobe and Ub^D^ noncovalent interface. **c**, Left, multilayered active site assembly: UBR5’s C-terminal tail, Ub^D^’s C-terminus linked to UBR5’s catalytic Cys, Ub^A^ and UBR5 N-lobe. Dotted lines represent electrostatic contacts. Di-Ub synthesis assays test effects of mutating UBR5’s C-terminus and UbA’s D58. Three repetitions gave similar results. A catalytically active UBR5-degradation-product is marked with an asterisk. **d**, Left, UBA domain interactions with Ub^A^. Right, di-Ub synthesis assays, performed four times gave similar results. **e**, Left, Ub^A^ interactions with UBR5’s C-lobe. Right, di-Ub synthesis assay testing effects of mutating Ub^A^’s A46 or UBR5’s Y2773 in interface. Three replicates showed similar results. **f**, Left, Ub^A^ interactions with UBR5 N-lobe. Right, di-Ub synthesis assay testing mutations of Ub^A^ R54, or UBR5’s E2287. Two replicates gave similar results. **g**, Left, close-up of LOL. Right, di-Ub synthesis assay testing effects of LOL mutated to Ala. Three replicates gave similar results. **h**, Left, close-up of Ub^A^ showing its residues that could be linked to other Ubs for branched chain formation. Right, tri-Ub synthesis assay with UBR5 and UBR5^Dimer^, testing di-Ubs with indicated linkages as acceptors. Coomassie-stained gels show di-Ub input. Assays performed three times gave similar results. Source data

Numerous previously unobserved interactions converge to form the linkage-specific Ub chain-forming machinery (Figs. 4d and 5a). First, interactions between the HECT domain C-lobe and Ub^D^ shape the active site (Fig. 5b). The map revealed the details of the noncovalent interface between UBR5’s C-lobe and Ub^D^, observed across the cryo-EM maps for intermediates along the cascade. An intermolecular hydrophobic core is formed among UBR5’s F2732, L2762, L2789 and A2790 and Ub^D^’s I36, P37, L71 and L73. The hydrophobic interactions are further buttressed by numerous polar contacts.

Second, the active site is configured by elements from Ub^D^’s C-terminal tail, UBR5’s C-terminal tail and the HECT domain catalytic loop wrapping around each other as if in a four-layered sandwich (Fig. 5c). At one edge, the so-called -4Phe^64^ (UBR5 F2796) packs between the N-lobe and Ub^D^’s C-terminus linked to the catalytic Cys^36^. On the other side of UBR5’s C-terminal tail, N2795 forms a hydrogen bond stabilizing the β-sheet between Ub^D^’s C-terminus and the HECT domain C-lobe strand that culminates at the catalytic Cys. Meanwhile, Ub^D^’s R72 and C-terminus wrap around UBR5’s penultimate F2798. F2798, in turn, secures the UBR5^Dimer^~Ub^D^ active site and inserts into the interface as a molecular glue affixing Ub^A^. Although UBR5’s C-terminal V2799 is not visible, modeling based on the orthologous residue in a prior HECT~Ub^D^ structure^36^ suggests that this could further contribute to luring Ub^A^ (Extended Data Fig. 4c). Accordingly, Ub chain formation is impaired by Ala substitutions for either F2796 or F2798 or deletion of V2799. The latter mutation may remove favorable interactions and/or introduce a repulsive charge into the interface.

Third, Ub^A^ is also positioned by the C-lobe-linked Ub^D^ (Fig. 5c). Ub^D^’s R72, itself oriented by UBR5’s penultimate F2798, contacts D58 from Ub^A^. Although it was not possible to test the effects of Ub R72 variants due to requirements for this residue in generating the E2~Ub^D^ intermediate^65,66^, a charge-repulsive D58R variant Ub^A^ substantially reduced di-Ub formation.

Fourth, UBR5’s UBA domain binds and presents Ub^A^ to the active site (Fig. 5d). The UBR5 UBA domain-Ub crystal structure^47^ readily fits into the map. This allowed visualizing details despite relatively lower resolution in this region presumably caused by flexibility of the tethers between the scaffold and UBA domain. Mutations replacing UBR5 L224 at the center of the interface, or interacting residues from Ub^A^, caused accumulation of the UBR5~Ub^D^ intermediate, and severely impaired Ub^D^ transfer to Ub^A^.

Fifth, Ub^A^’s residue 48 (normally K48 but here chemically modified Cys) is secured in the active site through interactions made by adjacent residues. On one side, Ub^A^’s A46 nestles opposite Y2773 from UBR5’s C-lobe (Fig. 5e). Accordingly, mutating Ub^A^’s A46 to Asp or Phe, which would hinder the structurally observed interface, impairs di-Ub synthesis. The impact of the mutations scale with their predicted structural effects: while Ub^A^ A46F would be too large for the pocket, A46D could potentially retain a suboptimal contact with UBR5’s Y2773. Notably, a UBR5 Y2773F variant, which could accommodate Ub^A^’s A46, shows WT Ub chain-forming activity. The ultimate test of the importance of an interface is restoration by compensatory changes. Thus, we assayed mutant combinations predicted to improve structural compatibility. Indeed, the smaller UBR5 Y2773F side chain partially restores activity with the Ub^A^ A46F variant, while loss of the hydroxyl on the E3 side accounts for further reduced activity with Ub^A^ A46D.

On the other side of Ub^A^’s residue 48, its R54 projects toward E2287 in the HECT domain N-lobe (Fig. 5f). Introducing a charge-repulsive R54E Ub^A^ variant severely impairs di-Ub synthesis. Eliminating that repulsion by a corresponding UBR5 charge-swap (E2287R) restored di-Ub synthesis.

Finally, the HECT domain N-lobe loop comprising residues D2283–E2287, which includes the abovementioned E2287, also secures the catalytic architecture by serving as a platform aligning the active site (Fig. 5g). We thus term this ligation-organizing loop (LOL). Replacing the LOL sequence with alanines specifically impaired Ub chain formation. Because this loop contains three acidic residues, we also performed assays at high pH to test the structural role by offsetting potential effects on acceptor Lys deprotonation (Extended Data Fig. 4d). The loop mutant was defective in all conditions tested.

### Catalytic architecture accommodates chain branching

UBR5 has been implicated in generating cellular branched poly-Ub chains^9,11^. Examination of the UBR5^Dimer^~Ub^D^~Ub^A^ structure provided insights into how branched Ub chain formation could occur if Ub^A^ was linked in a chain. Notably, Ub^A^’s C-terminal tail (not visible in the map) points away from the catalytic assembly (Fig. 5h). This suggests that the distal Ub in diverse chains, including K48-linked chains, could be readily modified by UBR5. Among Ub^A^’s lysines and the N-terminus, only residue 48 is fully buried by the catalytic assembly. Nonetheless, K11 stands out as most distal from the active site, with its primary amino group fully exposed. This is notable because UBR5 was suggested to produce a major fraction of cellular K11/K48-branched Ub chains^9^. Consistent with the UBR5^Dimer^~Ub^D^~Ub^A^ structure, both WT UBR5 and UBR5^Dimer^ modify all possible di-Ubs in vitro, with a preference for K11-linked chains.

## Discussion

**Extended Data Fig. 5:**
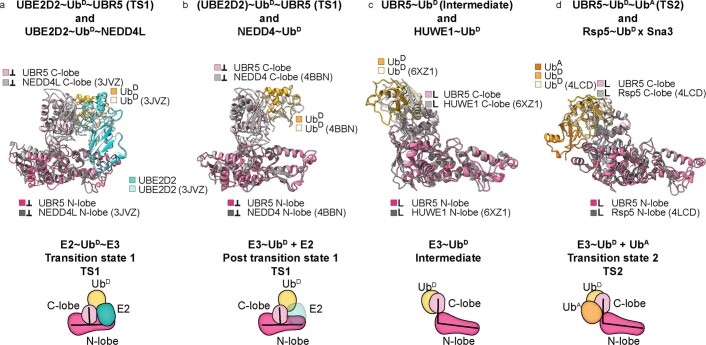
Conserved step-by-step conformational trajectory for HECT E3-catalyzed Ub transfer cascades. **a**, Structural superposition of UBE2D2~Ub^D^-bound HECT domains of UBR5 and NEDD4L (PDB: 3JVZ). The structures suggest E2 transfers Ub^D^ to HECT domains in the Inverted T-configuration. Colored boxes indicate the conformation of the respective complex. **b**, Structural superposition of Ub^D^ linked to HECT domain of UBR5 (TS1 model) and NEDD4 (PDB: 4BBN). This represents the Ub^D^-linked HECT domain in the Inverted T-configuration, immediately after Ub^D^ transfer from E2. **c**, Structural superposition of Ub^D^-loaded HECT domains of UBR5 (from UBR5^Dimer^~Ub^D^ intermediate) and HUWE1~Ub^D^ (PDB: 6XZ1), both in L-configuration. **d**, Structural superposition of HECT domains representing ubiquitylation complexes: K48-linked Ub chain formation by UBR5, and Ub^D^ transfer to a peptide substrate for Rsp5 (PDB: 4LCD). The structures suggest HECT domains transfer Ub^D^ to downstream targets - either substrates or acceptor Ub during polyubiquitylation - in the L-configuration.

Our collection of cryo-EM reconstructions reveals the structural basis for linkage-specific Ub chain formation by a HECT E3, and together with published work defines a conserved step-by-step conformational trajectory for HECT E3-mediated Ub transfer cascades. Although to date, progression across an entire ubiquitylation cascade has not been visualized for any other HECT E3 family member, there are crystal structures of HECT domains from four different E3s: human NEDD4L, NEDD4, and HUWE1, and yeast Rsp5—representing different states^33–36^. These superimpose on the various UBR5 structures and show HECT E3 ubiquitylation proceeds like a relay (Extended Data Fig. 5a–d). First, NEDD4L’s HECT domain receiving Ub^D^ from E2 (ref. ^33^) matches the Inverted T-configuration observed for TS1 of UBR5. This arrangement (1) copositions the N- and C-lobes and the E2~Ub^D^ conjugate, (2) extends the E2~Ub^D^ thioester bond, and (3) aligns the E2 and E3 active sites for Ub^D^ transfer between them^33^ (Extended Data Fig. 5a). All the structures further suggest that HECT C-lobes linked to Ub^D^ form a structural unit^33–36,67^. Second, after the formation of the E3~Ub^D^ conjugate, the E2 dissociates to allow this unit to swivel around the N-lobe to achieve the L-configuration (Extended Data Fig. 5b,c). Structural and/or biochemical data for Rsp5 (ref. ^34^), HUWE1 (ref. ^36^), Ufd4 (ref. ^68^) and UBR5 indicate that the L-configuration transfers Ub^D^ to a substrate, or to Ub^A^ during polyubiquitylation (Extended Data Figs. 4c and 5c,d). Thus, we propose that L-shaped HECT~Ub^D^ arrangements generally serve as catalytically active platforms that lure acceptors for modification. Furthermore, our data suggest the HECT E3 C-terminal tail arrangement allows penultimate hydrophobic side chains to serve as molecular glues between the donor and acceptor Ubs, at least during K48-linked Ub chain formation (Fig. 5).

**Fig. 6:**
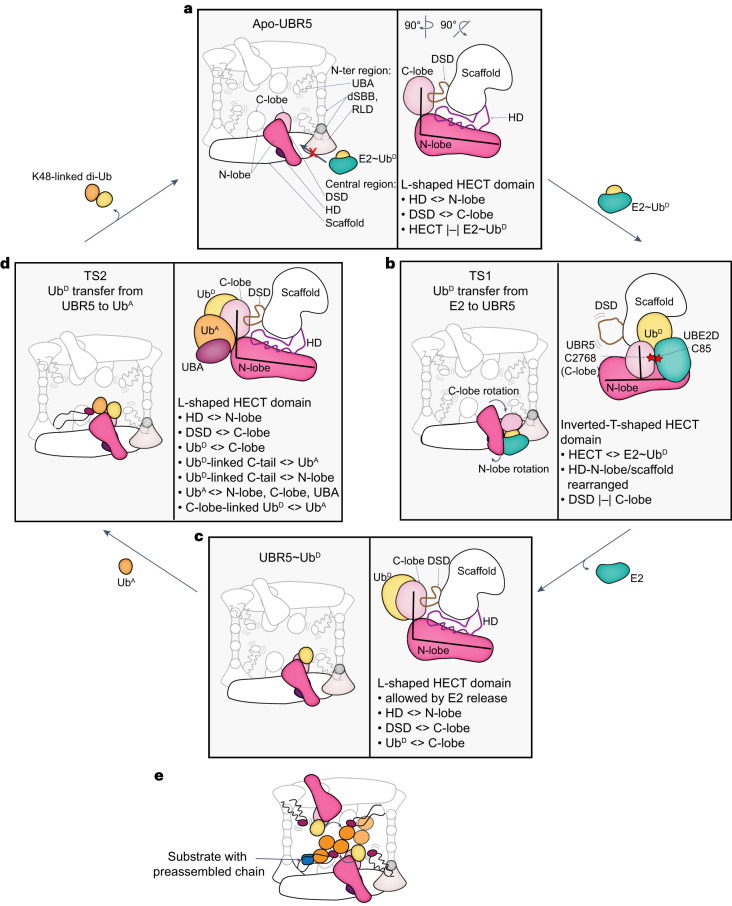
Feed-forward mechanism of UBR5 forming K48-linked Ub chains. **a**, UBR5 forms an oval tetramer composed of two dimeric, U-shaped multidomain assemblies, each containing a dimeric scaffold flexibly tethering UBA domains and connecting to HECT domains. Apo HECT domains adopt L-configuration, stabilized by HD and DSD domains, which is incompatible with E2~Ub^D^-binding. **b**, E2~Ub^D^ binding to HECT domain drives reorientation to Inverted Tconfiguration. **c**, Release of E2 after Ub^D^-transfer allows re-establishing scaffold connections with HECT domain in L-configuration. **d**, Ub^A^’s K48 and Ub^D^’s C-terminus linked to UBR5 are juxtaposed through multiple interactions, involving UBR5 regions and the Ubs being adjoined. **e**, Oligomerization could allow avid binding and modification of multiple Ub moieties within a chain.

Yet, UBR5’s catalytic HECT domain does not mediate polyubiquitylation on its own. This requires the acceptor Ub-binding UBA domain and is substantially potentiated by full-length UBR5 (Figs. 1h and 4c). UBR5 scaffold elements shape the Inverted T TS1 and L-shaped TS2 catalytic configurations by unique positive and negative interactions (Fig. 6 and Supplementary Video 2). The L-arrangement is stabilized by UBR5’s HD and DSD domains binding on one side to the scaffold, and on the other to the N- and C-lobes, respectively. E2 binding is incompatible with this arrangement between the scaffold and HECT domain. Instead, in the alternative arrangement observed for TS1, with the HECT domain in the Inverted T-configuration, E2 binding to the N-lobe is supported by the E2-linked Ub^D^ binding to the C-lobe. Subsequently, severance of the E2~Ub^D^ bond upon formation of the UBR5~Ub^D^ intermediate re-enables the L-configuration, which is further stabilized by interactions from the HECT C-terminal tail and Ub^D^. Ub^A^ is recruited by the UBA domain in a manner compatible with a distal Ub in any chain type and more proximal Ub in some. Ub^A^’s K48 is guided into the active site by interactions with UBR5’s LOL from the N-lobe, Ub^A^ binding site on the C-lobe and C-terminal tail, and by interactions with Ub^D^. After Ub^D^ transfer to Ub^A^, affinity of the produced chain for UBR5 would be diminished. Ub^D^ covalent linkage to UBR5 would be lost. Additional contacts would be lost upon dissolution of the UBR5 C-terminal tail structure that depends on E3 linkage to Ub^D^. This would reset UBR5 for another round of Ub chain formation. Thus, the conformational trajectory forging linkage-specific Ub chains is achieved by the E2~Ub^D^ and Ub^A^ substrates, and the reaction products, positively and negatively synergizing with numerous UBR5 structural features in a feed-forward manner.

It seems likely that interactions between N-terminal elements and the C-terminal HECT domain establish functions across the E3 family. Interestingly, another HECT E3 for which full-length structures are available, HUWE1, also shows its many N-terminal substrate-binding domains arranged in a scaffold interacting with a peripherally perched HECT domain^57,69^. However, HUWE1’s HECT domain is maintained in the autoinhibited conformation. Moreover, structures have also shown N-terminal regions autoinhibiting NEDD4-family HECT E3s^70–72^. Thus, the overall UBR5 architecture stands out for conformationally priming the HECT domain for activity toward pre-ubiquitylated substrates. Although future studies will be required to visualize how post-translational modifications and substrates further modulate HECT E3 catalytic architectures^32,37,73,74^, it seems that UBR5’s UBR and MLLE domains, facing or flexibly tethered to the HECT domain, respectively, are well situated to position recruited proteins adjacent to L-shaped UBR5~Ub^D^ active sites for modification^48,49,53,54,56^ (Extended Data Fig. 2a).

Finally, UBR5 is an oligomer (Fig. 1). Superimposing atomic models for each intermediate along Ub chain formation in the cryo-EM map of the tetramer shows their compatibility with this oligomeric state (Supplementary Video 3). Our structures show how multiple UBA domains simultaneously present acceptor Ubs to multiple active sites. The long flexible tethers connecting the UBA domains likely offer innumerable paths for them to avidly capture substrate-linked Ubs and present them for K48 modification (Fig. 6e). Thus, the structural data explain why UBR5 is a highly efficient K48-linked Ub chain-forming machine.

## Methods

### Construct design

All constructs in this study were generated using standard molecular biology techniques using the oligonucleotides described in Supplementary Table 2 and verified using Sanger sequencing. All constructs used for recombinant protein expression and subsequent protein purification are listed in Supplementary Table 3.

### Protein expression and purification

Expression of Ub, Ub variants and the E2 UBE2D was performed in *Escherichia* *coli* BL21(DE3) RIL cells. BL21(DE) RIL cells containing the respective plasmid were grown at 37 °C in TB medium, supplemented with appropriate antibiotics. Upon reaching an optical density of 0.8, temperature was lowered to 18 °C and expression was induced by adding isopropyl β-D-thiogalactopyranoside (IPTG) to a final concentration of 0.6 mM. Consequently, expression was conducted for 18 h, before collecting cells by centrifugation at 4 °C for 15 min, 4,500*g*. The pellet was resuspended in ice-cold lysis buffer containing 50 mM Tris–HCl (pH 8.0), 200 mM NaCl, 5 mM dithiothreitol (DTT) or 5 mM β-mercaptoethanol for His-tagged constructs, 2.5 mM phenylmethylsulfonyl fluoride (PMSF), and additionally 10 μg ml^−1^ leupeptin, 20 μg ml^−1^ aprotinin and 10 μg ml^−1^ DNase for constructs expressed in insect cells or HEK293S cells. Cells were lysed by sonication on ice, and lysate was precleared by centrifugation for 40 min at 4 °C at 20,000*g*.

#### His-tagged proteins

His-tagged acceptor Ubs were purified via Ni-NTA affinity chromatography in a gravity flow column setup. The resin was washed with 20 mM imidazole, 50 mM Tris–HCl (pH 8.0), 200 mM NaCl and 5 mM β-mercaptoethanol. Elution of specifically bound proteins was achieved with 300 mM imidazole. Subsequently, size-exclusion chromatography was performed in 25 mM 2-[4-(2-Hydroxyethyl)piperazin-1-yl]ethane-1-sulfonic acid (HEPES) (pH 7.5) and 150 mM NaCl at 4 °C, which yielded pure His-tagged protein.

#### GST-tagged proteins

E2 enzymes, single lysine acceptor Ubs and M1-linked di-Ub were purified by incubating precleared lysate with glutathione-S-transferase (GST)-sepharose resin for 1 h at 4 °C. After extensively washing the resin (50 mM Tris–HCl (pH 8.0), 200 mM NaCl and 5 mM DTT), GST-fusion protein was eluted using 10 mM reduced glutathione. Cleavage of the GST-tag was achieved using proteolytic digest overnight at 4 °C with His-tagged human rhinovirus (HRV) 3C protease for single lysine Ub-constructs or His-tagged tobacco etch virus (TEV) protease for E2 enzymes and M1-linked di-Ub. E2 and di-Ub protein was subsequently purified using ion-exchange chromatography and size-exclusion chromatography at 4 °C with final buffer of 25 mM HEPES (pH 7.5) and 150 mM NaCl (+1 mM DTT for E2). The single lysine Ubs were further purified using size-exclusion chromatography in a buffer containing 25 mM HEPES (pH 7.5) and 150 mM NaCl subsequent to affinity purification.

#### Fluorescently labeled Ub

Donor Ubs, which were ultimately fluorescently labeled, were expressed as GST-3C-fusions in a pGEX-vector with an additional N-terminal cysteine. In brief, GST-affinity chromatography followed by HRV 3C protease cleavage was performed. Next, size-exclusion chromatography into 50 mM HEPES (pH 7.5), 200 mM NaCl and 5 mM DTT was carried out. The high concentration of reducing reagent is crucial to ensure complete reduction of the N-terminal cysteine, which is modified later. Before coupling to maleimide, DTT was removed by desalting twice with Zeba Spin desalting columns into reaction buffer (50 mM HEPES (pH 7.5) and 150 mM NaCl). Fluorescein-5-maleimide or tetramethylrhodamine-5-maleimide (TAMRA) was resuspended in anhydrous dimethylsulfoxide (DMSO) and added to desalted Ub in a 10x fold molar excess while keeping the final DMSO concentration below 5%. The reaction was incubated for 2 h at room temperature before being quenched by the addition of 10 mM DTT. Consequently, desalting of samples was repeated to remove unreacted maleimide, followed by two size-exclusion chromatography runs to yield highly pure labeled Ub conjugates. Di-Ub synthesis assays were performed with TAMRA-labeled Ub with Lys 48 mutated to Arg to prevent its use as acceptor. The autoubiquitylation assay to compare UBR5 and UBR5^Dimer^ as well as the polyubiquitylation assay to compare UBR5 with the SDA mutant were performed using fluorescein-labeled WT Ub.

#### Tagless Ub via acidic precipitation

Tagless Ubs were used as acceptors in the di-Ub synthesis assay in Fig. 1h, as basis for the generation of various Ub chains, which were used as acceptor di-Ubs in the tri-Ub synthesis assay in Fig. 5h, and added to improve cryo-EM samples. Additionally, Ub^K48C^ was one of the building blocks for the formation of the di-Ub probe. To obtain tagless Ub, it was expressed using a pET22b vector. Expression as well as cell lysis was conducted as described. Next, acetic acid (glacial) was slowly added to the lysate until a pH of ~4.5 was reached to precipitate out other proteins except for Ub. Subsequent ion-exchange chromatography of the cleared supernatant was followed by size-exclusion chromatography into 25 mM HEPES (pH 7.5) and 150 mM NaCl (+1 mM DTT in case of Ub^K48C^) at 4 °C to yield tagless Ub and Ub variants.

#### Insect cell-derived proteins

Expression of the isolated UBR5 HECT domain, with or without interrupting MLLE domain, as well as a UBA–HECT fusion, was performed in Hi5 insect cells. These constructs contained an N-terminal GST-tag followed by a TEV-cleavage site. Additionally, human UBA1 was also expressed as GST-TEV fusion in the same cell system. Cell lysis and preclearance of the lysate were performed as described for bacterial expressions. Protein purification was performed at 4 °C, using gravity flow affinity purification with GST-resin, followed by proteolytic cleavage of GST-fusion protein with His-tagged TEV protease. Finally, ion-exchange chromatography and size-exclusion chromatography were carried out with the final buffer consisting of 25 mM HEPES (pH 7.5), 150 mM NaCl and 1 mM DTT.

#### Expression and purification of human UBR5

The GFP-UBR5 plasmid was a gift from D. Saunders (Addgene plasmid 52050; http://n2t.net/addgene:52050; RRID: Addgene_52050)^75^ and was recloned into a pEG vector to enable its recombinant expression in HEK293S cells using the BacMam system. The starting gene contained a K503R point mutation and therefore, all UBR5 constructs used here also contain this mutation even though being referred to as WT throughout this study. Baculovirus of the respective construct was prepared using Sf9 cells and used to infect HEK293S cells that were grown to a cellular density of ~3 Mio cells per ml in Dulbecco’s modified Eagle medium, supplemented with 10% fetal calf serum. To improve UBR5 expression, cells were additionally supplemented with 100 μM ZnSO_4_. Eight hours postinfection, sodium butyrate was added to a final concentration of 10 mM and cells were grown for 60 h at 30 °C. Cells were collected by centrifugation for 15 min, 450*g* at 4 °C, resuspended in lysis buffer and lysed as described above. All purification steps were performed at 4 °C. TwinStrep-tagged GFP-fusion protein was isolated using Strep-affinity chromatography, followed by overnight cleavage with His-tagged HRV 3C protease. The next day, size-exclusion chromatography was carried out. For UBR5^C2768A^ and UBR5^Dimer^, which were used for the collection of the apo-UBR5 datasets, the final size-exclusion buffer consisted of 25 mM HEPES (pH 7.5), 150 mM NaCl and 1 mM DTT. Other UBR5 variants including UBR5^Dimer^, used for cryo-EM on the distinct ubiquitylation transition states, were purified in a final buffer containing 25 mM HEPES (pH 7.5), 150 mM NaCl and 1 mM tris(2-carboxyethyl)phosphine hydrochloride (TCEP). Despite intense efforts to circumvent this, sodium dodecylsulfate polyacrylamide gel electrophoresis (SDS–PAGE) gel analysis revealed degradation products of UBR5 after size-exclusion chromatography. The identity of these truncated species was confirmed to be UBR5 by mass spectrometry analysis and remains somewhat catalytically active. The SDS–PAGE band(s) corresponding to the modified truncations are marked with ‘*’ in Fig. 1b,h, Fig. 2d,e, Fig. 5c,d,e,f,g,h, Extended Data Fig. 1a,f, and Extended Data Fig. 3c,f,h.

### Mass photometry

To determine the oligomeric state of UBR5, mass photometry measurements were performed on the Refeyn TwoMP using Refeyn AcquireMP 2.3.0 software. Mass calibration was achieved by measurement of a protein mixture, providing a range of molecular masses as follows: conalbumin (75 kDa), aldolase (158 kDa), ferritin (440 kDa) and thyroglobulin (669 kDa) in a final concentration of ~50 nM of each component. Measurements of either UBR5 or UBR5^Dimer^ were carried out by diluting UBR5 to a final concentration of ~140 nM in the same buffer used for focus-finding (25 mM HEPES (pH 7.5), 150 mM NaCl and 1 mM DTT). Videos were collected for 1 min. Data were then analyzed using DiscoverMP 2.3.0 (Refeyn) software and the collected mass calibration as a reference.

### Generation of TS1 and TS2 probes

#### Semi-synthesis of deprotected Ub–BmDPA

Deprotected Ub-(E)-3-(2-(bromomethyl)-1,3-dioxolan-2-yl)prop-2-en-1-amine (Ub-BmDPA) is the building block of our activity-based probes, which enabled obtaining structures representing the conformations of assemblies mediating the transfer of Ub^D^ from E2 to E3 and subsequently from E3 onto an acceptor Ub. With these approaches, we are trying to mimic native geometry as closely as possible. For this, His-Ub(1-75)-intein–chitin-binding domain (CBD) was expressed in *E. coli*, and cells were lysed in buffer containing 20 mM Tris–HCl (pH 6.8), 50 mM NaOAc, 100 mM NaCl and 2.5 mM PMSF. His-Ub(1-75)-intein-CBD was purified using Ni-NTA affinity chromatography, and intein-based cleavage was induced by the addition of 100 mM MESNa^76^. The resulting His-Ub(1-75)-MESNa was then purified using size-exclusion chromatography in a final buffer of 25 mM HEPES (pH 6.8), 100 mM NaCl and 20 mM NaOAc. The hydrolysis ratio of His-Ub(1-75)-MESNa was analyzed using liquid chromatography coupled to mass spectrometry (LC–MS) and was taken into account for all further steps. To convert the obtained His-Ub(1-75)-MESNa into chemical proxies, the thioester group was modified. His-Ub(1-75)-MESNa (10 mg ml^−1^) was first coupled to 0.4 M BmDPA (ChiroBlock) in the presence of 1 mM N-hydroxysuccinimide in 10% (vol/vol) DMSO and 50 mM HEPES (pH 6.8). After incubating the sample overnight at 30 °C, 300 rpm, it was desalted into 25 mM HEPES (pH 6.8) and 100 mM NaCl, and completion of the reaction was confirmed using LC–MS. Next, the product was deprotected by incubating it at a concentration of ~1 mg ml^−1^ in 40 mM p-toluenesulfonic acid and 54% (vol/vol) trifluoroacetic acid (TFA) for 1 h at room temperature. Removal of TFA was achieved by washing the suspension several times with ice-cold diethyl ether. After air-drying the obtained Ub flakes, they were resuspended in 100 mM Na_2_HPO_4_ (pH 6.0), 500 mM NaCl and 8 M urea, and the protein was refolded by dialysis in 20 mM Na_2_HPO_4_ (pH 6.0) and 100 mM NaCl overnight at 4 °C.

#### Sortase-mediated transpeptidation of carboxyfluorescein–PEG5–LPETGG to UBE2D2

Due to UBR5’s large size of >300 kDa, another read-out for the reactivity of the UBE2D2–BmDPA–Ub probe apart from a mass shift on an SDS–PAGE gel of UBR5 upon reaction was required. Therefore, we aimed to fluorescently label the E2 enzyme and enable visualization of probe conjugates via fluorescence detection on a Typhoon Scanner. To achieve this, we designed a fluorescein-labeled LPETGG peptide with a PEG5 linker between the fluorophore and sortase recognition sequence (CF–PEG5–LPETGG, MPIB core facility). We then took advantage of the Gly–Ser remnant at the N-terminus of UBE2D2 that remains after proteolytic TEV cleavage. This was recognized by sortase A as a substrate for the transpeptidation reaction with the labeled peptide. Thus, we incubated 6× fold molar excess of labeled peptide (300 µM) with 50 µM UBE2D2 (C21A/C107A/C111S)^77^ and 5 µM His-tagged sortase A for 1 h at room temperature in 50 mM Tris–HCl (pH 8.0), 150 mM NaCl and 10 mM CaCl_2_. Sortase A was removed via His-affinity chromatography, and the E2 enzyme-containing flow-through was collected. Subsequently, the labeled E2 enzyme was purified via size-exclusion chromatography in 50 mM HEPES (pH 7.5) and 150 mM NaCl.

#### Formation of UBE2D2–Ub probe for TS1 mimic

The TS1 probe creates a stable mimic representing Ub transfer from the catalytic cysteine of UBE2D2 to UBR5’s catalytic cysteine. The E2 enzyme was incubated with 1 mM TCEP for 30 min at room temperature to ensure complete reduction of the catalytic cysteine and was then desalted into reducing reagent-free buffer before conversion with Ub–BmDPA. For the generation of unlabeled UBE2D2–Ub-probe, we incubated 100 µM of deprotected Ub–BmDPA with 5× fold molar excess of UBE2D2^C21A,C107A,C111S^ for 2 h at 30 °C. Excess E2 enzyme was removed via His-affinity chromatography and further purified by size-exclusion chromatography in 25 mM HEPES (pH 7.5) and 150 mM NaCl. To obtain a fluorescently labeled UBE2D2–Ub probe, the molar excess was reversed due to the limited yield of the labeled E2. Consequently, 50 µM of fluorescein-UBE2D2 was incubated with 5× fold molar excess of Ub–BmDPA (250 µM) for 2 h at 30 °C. The conjugated probe was purified via size-exclusion chromatography in 50 mM HEPES (pH 7.5) and 150 mM NaCl. A synthesis scheme is shown in Extended Data Fig. 3a,b and was prepared using ChemDraw v22.2.0.

#### Generation of Ub^D^–Ub^A^ as TS2 probe

Deprotected Ub–BmDPA was also the building block for the TS2 probe, which creates a stable mimic representing the assembly mediating transfer of Ub^D^ from UBR5’s catalytic cysteine to Ub^A^’s K48. To generate this reactive probe, the targeted lysine on Ub^A^ (K48) was mutated to Cys. It was essential for Ub^K48C^ to be incubated with fresh reducing reagent (1 mM TCEP) before the conjugation reaction. After desalting into 25 mM HEPES (pH 7.5) and 150 mM NaCl, Ub^K48C^ was incubated with 5× fold excess of deprotected Ub–BmDPA for 1 h at 30 °C in the aforementioned buffer. Excess protein was removed using size-exclusion chromatography with the final buffer consisting of 25 mM HEPES (pH 7.5) and 150 mM NaCl. The synthesis scheme is shown in Extended Data Fig. 4a,b. Chemical structures were drafted using ChemDraw v22.2.0.

### Cryo-EM sample preparation, data collection and processing

Cryo-EM data collection, refinement and validation statistics for all presented datasets are summarized in Supplementary Table 1. All data acquisitions were set up using SerialEM v.3.8.0-b5 and FEI EPU v2.7.0.

#### Tetrameric UBR5^C2768A^

Structure determination by single-particle cryo-EM is typically facilitated by use of purified material. During the course of this study, we obtained sufficient amounts of purified catalytically inactive UBR5^C2768A^ protein before performing a large-scale purification of WT UBR5. Thus, we proceeded with cryo-EM of UBR5^C2768A^ with the intent to guide further studies. UBR5^C2768A^ was freshly purified as described earlier and supplemented with 3-((3-cholamidopropyl)-dimethylammonio)-2-hydroxy-1-propanesulfonate (CHAPSO) shortly before plunging (8 mM). In total, a 3.5 μl sample concentrated to 2.5 mg ml^−1^ was applied onto freshly glow discharged R1.2/1.3, Cu 200 mesh holey carbon grids (Quantifoil) using a Vitrobot Mark IV (Thermo Fisher Scientific) at 100% humidity, 4 °C. Grids were blotted for 3 s with blot force 4 and plunge-frozen into liquid ethane. Data were collected on a Titan Krios transmission electron microscope (TEM) operating at 300 kV, equipped with a post-GIF Gatan K3 Summit direct electron detector in counting mode. Frames were recorded at a nominal magnification of 64,000× fold and a pixel size of 1.384 Å per pixel at the specimen level with a target defocus range of −2.4 μm and −0.8 μm and total exposure of 56.28 electrons per Å^2^. In total, 10,091 micrographs were collected, and alignment as well as dose-weighing was performed using MotionCorr2 (ref. ^78^) followed by contrast transfer function (CTF) estimation using GCTFv1.06 (ref. ^79^). Screening datasets of UBR5^C2768A^, collected on a Glacios TEM at 200 kV and equipped with a Gatan K2 detector in counting mode, yielded an initial model. This model was obtained using RELION 3.1.1 (ref. ^80^) and was used as reference. Severe preferred orientation of particles was observed in screening datasets but could be reduced by addition of detergent. In total, 1.1 million particles were picked using template-based picking with Gautomatch (K. Zhang, MRC Laboratory of Molecular Biology). Two-dimensional (2D) classification, followed by extensive 3D classification, 3D refinements, CTF refinement, particle polishing and postprocessing were performed using RELION 3.1.1 (ref. ^80^). Binning of the particles was lifted stepwise during 3D classifications. Comparison of 3D classes revealed breathing motions of the upper and the lower dimeric unit with respect to each other.

A final map was generated by applying C2 symmetry during 3D refinement followed by map-sharpening using either DeepEMhancer^81^ (shown in Fig. 1) or postprocessing, resulting in a map of 3.7 Å. The intrinsic flexibility of the two dimeric units severely impacted the map quality of the lower half of the map. The processing scheme is depicted in Supplementary Fig. 1.

#### UBR5^Dimer^

The mutant UBR5^L710D^, referred to as UBR5^Dimer^, was supplemented with n-octyl-β-d-glucopyranoside (β-OG) at 0.1% (wt/vol) shortly before plunging it at a concentration of 1.3 mg ml^−1^. Plunging and data collection were carried out as described for UBR5^C2768A^. Frames were collected at 105,000× fold magnification with a pixel size of 0.8512 Å per pixel, a target defocus range of −3.0 to −0.5 and a total exposure of 67.8 electrons per Å^2^. In total, 21,270 micrographs were collected and subjected to alignment and dose-weighing as described above. Once again, Glacios microscope-derived screening datasets of UBR5^Dimer^ yielded an initial model that was used as 3D reference and template for particle picking. Template-based particle picking resulted in 762,722 particles. Data processing was performed using RELION 4.0 (ref. ^82^). 2D classification, followed by 3D classification, 3D refinements and two iterative rounds of CTF refinement as well as particle polishing resulted in a 2.7 Å map after applying C2 symmetry during 3D refinement and map-sharpening using postprocessing or DeepEMhancer^81^.

Converting UBR5 to a dimeric species substantially reduced preferred orientations.

Polished and refined particles of the final 3D refinement were transferred from RELION to CryoSparc4.2.0 (ref. ^83^) to improve density around regions corresponding to the RLD and DSD domains. Nonuniform refinement and local refinement were carried out, covering the RLD, a part of the scaffold, and the proximal HECT domain^83,84^. With the local refinement map in hand, DeepEMhancer implemented in CryoSparc^83^ was used to calculate a map with a final resolution of 2.98 Å. While the overall resolution is lower, local resolutions are much higher in the RLD region compared to the full map. The processing scheme is depicted in Supplementary Fig. 2.

#### UBE2D2~Ub^D^~UBR5^Dimer^: TS1

To mimic the fleeting TS1, a chemical mimic was used. This stable proxy contains three additional atoms connecting the catalytic cysteines of E2 and UBR5, compared to the native transition state.

A fluorescent version of the TS1-probe was used to determine whether probe reactivity was dependent on the catalytic cysteine of UBR5. For this, UBR5 or the catalytically inactive UBR5^C2768A^ was mixed with ~5× fold molar excess of probe and incubated at room temperature for the indicated time points. In-gel fluorescence was measured after performing SDS–PAGE to show progression of the probe reaction (Extended Data Fig. 3c). Notably, UBR5^C2768A^ exhibited base-levels of fluorescent signal, possibly due to incomplete proteolytic cleavage of the GFP-tag.

Cryo-EM sample preparation was performed by incubating UBR5^Dimer^ with equimolar amount of K63-linked tetra-Ub chain and 2× fold molar excess of UBE2D2~BmDPA~Ub for 2 h on ice. The reaction mix was plunged without further purification at a concentration of 2 mg ml^−1^.

Incubation with β-OG (Sigma-Aldrich) at a final concentration of 0.1 % (wt/vol) resulted in a higher density of particles. The detergent was added shortly before applying 3 μl of UBE2D2~Ub^D^~UBR5^Dimer^ to freshly glow-discharged holey carbon grids (Quantifoil, R1.2/1.3, 200 mesh). The sample was consequently plunge-frozen into liquid ethane at 95% humidity and 4 °C (blot force 3, blot time 4 s). Data were collected on thin ice with an Arctica electron microscope, equipped with a Falcon III electron detector in linear mode. Frames were collected at a nominal magnification of 73,000, equaling 1.997 Å per pixel at the specimen level. The target defocus ranged between −3.5 and −1.0 μm and a total exposure of ~70 electrons per Å^2^ was distributed over 40 frames.

RELION 4.0 (ref. ^82^) was used for motion correction and dose-weighing of 1,740 micrographs. The contrast transfer function was estimated using CTFFIND-4.1 (ref. ^85^). The structure of UBR5^Dimer^ with the HECT domains in L-configuration was used as template for picking with Gautomatch and as 3D reference. In total, 1.4 million particles were extracted (2.1× binned) and subjected to extensive 3D classification. While structures of the initial 3D classification resembled the reference structure with both HECT domains in L-conformation, further classifications displayed either one of the HECT domains in an Inverted T conformation with Ub conjugated to the C-lobe. At the same time, sample heterogeneity became apparent as the HECT domains of both UBR5 protomers would adopt either L, Inverted T or a mix of both conformations. Extensive 3D classification was performed to visualize combinations of HECT conformations and classify out single, stable conformations. This was followed by focused classification with the newly obtained HECT in Inverted T-conformation as reference to search for the most stable Inverted T-conformation with robust Ub density.

After deriving a clean set of 46,615 particles with one of the two HECT domains fixed in the Inverted T-conformation, particles were extracted to full pixel size. Finally, the particle set was refined to 7.3 Å and the 3D reconstruction was sharpened using either RELION postprocessing or DeepEMhancer^81^. The processing scheme is depicted in Supplementary Fig. 3.

#### UBR5^Dimer^~Ub-VME: intermediate state

A stable mimic of the E3~Ub^D^ intermediate was generated using Ub-VME. Using this chemical proxy, native distance between the UBR5 catalytic Cys and Ub^D^ could be retained.

Ub-VME was synthesized as described in the Supplementary Note.

To test whether the reactivity of Ub-VME depends on the catalytic cysteine of UBR5, Rho-Ub-VME was incubated with either UBR5 or the catalytically inactive UBR5^C2768A^ at ~5× fold molar excess and incubated at room temperature for indicated time points. SDS–PAGE with a subsequent in-gel fluorescence measurement exhibited reacted species (Extended Data Fig. 3h). Background signal of UBR5^C2768A^ even in the absence of probe indicates remnants of uncleaved GFP-labeled UBR5.

To prepare the sample for cryo-EM, UBR5^Dimer^ was incubated with equimolar amount of K63-linked tetra-Ub chain and 10× fold molar excess of Ub-VME for 2 h at room temperature. The reaction mix was consequently subjected to size-exclusion chromatography (25 mM HEPES (pH 7.5), 150 mM NaCl and 0.5 mM TCEP), and peak fractions were concentrated to 1.5 mg ml^−1^.

Shortly before plunging, CHAPSO (Sigma-Aldrich) was added to the protein sample at a final concentration of 8 mM. Subsequently, holey carbon grids (Quantifoil, R1.2/1.3, 200 mesh) were glow discharged, and 3 μL of UBR5^Dimer^~Ub-VME were applied to the grid at 95% humidity and 4 °C in a Vitrobot Mark IV (Thermo Fisher Scientific) and plunge-frozen into liquid ethane (blot force 3, blot time 4 s). Data were collected on medium-thick ice with a Glacios electron microscope, equipped with a K2 Summit direct electron detector in counting mode. Frames were collected at a nominal magnification of 22,000×, equaling 1.885 Å per pixel at the specimen level. The target defocus ranged between −2.6 and −0.8 μm, and a total exposure of ~60 electrons per Å^2^ was distributed over 40 frames.

RELION 4.0 (ref. ^82^) was used for motion correction and dose-weighing of 1,808 micrographs. The contrast transfer function was estimated using CTFFIND-4.1, and particles were picked template-free with Gautomatch. In total, 834,722 particles were extracted (2.3× binned) and subjected to 3D classification with the structure of UBR5^Dimer^ serving as 3D reference. After the first round of classification, 3D structures displayed robust density for Ub, conjugated to the C-lobe of each protomer. No un-conjugated C-lobe could be observed during the processing of the dataset, suggesting complete reaction of UBR5 with Ub-VME. However, while secondary structures were visible for the HECT domain, Ub density was less defined and of lower resolution-implying flexibility. To investigate this, re-extracted particles to full pixel size of the refined UBR5^Dimer^~Ub-VME structure were imported to CryoSparc to perform 3D-VA^62^. Default parameters were used for the 3D-VA with structures being low-pass filtered to 9 Å. Substantial movements of the Ub-conjugated C-lobe and the dSBB domain could be observed, justifying lower resolution of those domains. The final masked refinement of UBR5^Dimer^~Ub-VME with 197,281 particles yielded a 3D reconstruction at 5.3 Å and was sharpened using either RELION postprocessing or DeepEMhancer^81^. The processing scheme is depicted in Supplementary Fig. 4.

#### UBR5~Ub^D^~Ub^A^ TS2

To visualize the UBR5 complex as if in action of forming a K48-linked Ub chain, a stable mimic of TS2 was generated that retained native distances between UBR5’s catalytic Cys and the α-carbon of Ub^A^. Transfer of Ub^D^ from the catalytic cysteine of UBR5 to Ub^A^ was mimicked by conjugation of UBR5^Dimer^ with a ~50× fold molar excess of the Ub^D^–BmDPA–Ub^A^ in 25 mM HEPES (pH 7.5), 150 mM NaCl and 1 mM TCEP for 2 h at room temperature. UBR5~Ub^D^~Ub^A^ was purified at 4 °C using size-exclusion chromatography in a final buffer of 25 mM HEPES (pH 7.5), 150 mM NaCl and 1 mM TCEP. The peak fractions were pooled and concentrated to 0.6 mg ml^−1^ and subsequently supplemented with CHAPSO (final detergent concentration 8 mM). Plunging was performed as described for UBR5^C2768A^, and a dataset was collected on a Glacios screening microscope with a target defocus of −3.0 and −0.3 μm and a total exposure of ~60 electrons per Å^2^ partitioned into 40 frames. RELION 3.1.1 was used for motion correction and dose-weighing of 705 micrographs. The contrast transfer function was estimated using GCTF. A low-pass filtered map of UBR5^Dimer^ was used as template for picking with Gautomatch and as initial 3D reference. In total, ~300,000 particles were picked and subjected to 2D and 3D classification. 3D refinement with subsequent sharpening using postprocessing or DeepEMhancer^81^ resulted in an 8.3 Å resolution map that had substantial additional density next to the C-lobe of both HECT domains. Its overall architecture resembled UBR5^Dimer^~Ub^D^; however, additional density next to the C-lobe and Ub^D^ could accommodate Ub^A^ and the UBA domain.

To improve map quality of the HECT domain linked to Ub^D^ and Ub^A^ and further revealing the Ub^A^-binding UBA domain, a second dataset was collected on UBR5~Ub^D^~Ub^A^ on a Titan Krios electron microscope with a magnification of 105,000× fold, a pixel size of 0.8512 Å per pixel and a defocus range of −2.2 to −0.6. In total, 17,689 micrographs were collected, and alignment as well as dose-weighing were performed as described for UBR5^C2768A^ and UBR5^Dimer^. Template-based picking with the UBR5^Dimer^ model resulted in 1.7 million particles. 2D classification as well as several rounds of 3D classification were performed using RELION 3.1.1 (ref. ^80^). A mask was created covering the HECT domain, Ub^A^ and Ub^D^, as well as the neighboring UBA domain, and two rounds of masked 3D classifications were carried out. Next, classified particles and the 3D reconstruction were imported to CryoSparc to further resolve the HECT domain (including observing for the first time its C-terminal tail) bound to Ub^D^ and Ub^A^, as well as the UBA domain. Nonuniform refinement^84^ followed by local refinement, focusing on the HECT domain, Ub^A^, Ub^D^, and the UBA domain was performed and a resolution of 3.3 Å was achieved. The map was sharpened using the implemented DeepEMhancer^83^. Processing schemes for both UBR5^Dimer^~Ub^D^~Ub^A^ datasets are depicted in Supplementary Fig. 5.

### Model building and refinement

#### Model for tetrameric UBR5

An initial model of UBR5 was generated using AlphaFold2. The obtained model was split into smaller parts, which were docked into the density map obtained for UBR5^C2768A^ using UCSF Chimera. This allowed the determination of residue L710 for mutagenesis to disrupt the interaction connecting both U-shaped units.

#### Structure for UBR5^Dimer^

The high-resolution map of UBR5^Dimer^ allowed precise building of the protein backbone including side chains in most parts of the structure using COOT v.0.9.6 (ref. ^86^). Due to the low resolution for the dSBB domain, the barrels could not be built but were docked into the structure using the AlphaFold2 model instead. Unfortunately, we were not able to determine how residues 1523–1773 connect at the heterodimerization interface unambiguously. For this reason, we assigned them as four separate chains in the coordinates. However, we note that AlphaFold2 predicts the structural connection between these, and protomers were therefore assigned on this basis for figures. To build the RLD and DSD domain, the focused map of UBR5^Dimer^ was used due to better map quality in those regions. Because density for the connection of the DSD domain to the scaffold was missing, it could originate from either monomer and was therefore kept as separate chain. However, the closer distance of the monomer in *trans* suggests that the DSD domain originates there and integrates into the other monomer to increase the dimerization interface.

In early refinement cycles, twofold symmetry was applied to the structure of a monomer to obtain the structure of a dimer. Finally, several rounds of real-space refinement were performed using PHENIX v1.19.2 (ref. ^87^). The atomic model of UBR5^Dimer^ was validated using Molprobity v.4.2 (ref. ^88^). A complete summary of data collection and refinements statistics were provided in Supplementary Table 1.

#### Model for UBE2D2~Ub^D^~UBR5^Dimer^ TS1

To generate a model for the TS1 intermediate (UBE2D2~Ub^D^~UBR5^Dimer^), the structure of UBR5^Dimer^ was split into several parts—the scaffold that could be readily docked into the density, the HD domain and the N-lobe of the HECT domain, which had to be tilted slightly compared to the apo-UBR5-structure. The HECT domain C-lobe had to be massively rearranged. UBE2D2~Ub^D^ was extracted from a preexisting structure where it was bound to NEDD4L^33^ (PDB: 3JVZ). Using UCSF Chimera, these components were fitted into the obtained map. Note that UBE2D2 in 3JVZ has the catalytic cysteine mutated to serine to generate a more stable oxyester-linked complex. Instead, UBE2D2 used in our study retains the catalytic cysteine; however, the three remaining cysteines are mutated as follows: C21A, C107A and C111S^77^. Because the obtained density for the TS1 complex only had one HECT domain positioned in the Inverted T-conformation (HECT 1) and the other HECT domain presumably being a mix of L- and Inverted T-conformation (HECT 2), the model only applies to HECT domain 1.

#### Model of UBR5^Dimer^~Ub^D^ intermediate

An initial model for the intermediate was made based on a previously published structure of Rsp5~Ub-Sna3 (ref. ^34^; PDB: 4LCD) and the structure of UBR5^Dimer^. The Protein Data Bank (PDB) 4LCD was fitted into the density for HECT domain 2 using UCSF Chimera. Subsequently, Ub^D^ was extracted from this PDB and docked into the density individually. The final model for the intermediate state, shown in Fig. 3, was built based on the structure for TS2. Because the HECT~Ub^D^ conformation of the TS2 structure fits nicely into the density, these parts were docked into the density together with the remaining parts of the UBR5^Dimer^ structure. Because the EM map does not exhibit density for the C-terminus of UBR5 and C-terminus of Ub^D^ (also differing in the reactive group compared to TS2), these parts were truncated for the final model of UBR5^Dimer^~Ub^D^. Although the map reveals clear density for Ub^D^ on both HECT domains, our model only focuses on one HECT domain bound to Ub^D^ (HECT 2).

#### Structure of UBR5~Ub^D^~Ub^A^: TS2

To build the structure mimicking TS2, the model of UBR5~Ub^D^ and the crystal structure showing UBR5’s UBA domain bound to Ub (PDB: 2QHO) were docked into the focused map. The map quality largely allowed building of the HECT domain including both Ubs (that is, Ub^A^ and Ub^D^), mostly on a side-chain level using COOT^86^. In this model, a K48C variant of Ub^A^ was introduced. There is clear density for UBR5 residues 2796–2798 (Phe–Gly–Phe), although the precise locations of side chains were ambiguous. For F2796, density was smeared, suggesting potential conformational heterogeneity for this residue. For F2798, lack of clear visibility for the subsequent, terminal residue precludes absolute determination if the density corresponds to the main chain or the side chain. The density is tentatively assigned to the side chain, based on similarity to the corresponding residue in the structure of a HUWE1 HECT domain complex with Ub^36^ and the biochemical effect of mutations, but its unambiguous placement will require future studies. Different UBR5 truncations, as well as a variety of different UBR5~Ub complexes, were subjected to Alphafold2, or Alphafold2.2 multimer, respectively, to provide potential insights into UBR5’s C-terminus. However, we note, that neither of the generated models resembled the C-terminus of UBR5 that could still be built with high confidence, yet the density followed the same trajectory as in the previously published crystal structure of HUWE1’s HECT domain bound to Ub (PDB: 6XZ1)^36^ (Extended Data Fig. 4c). Side chains of R72 and R74 of Ub^D^ were slightly moved to allow placement of UBR5’s C-terminus. Placement of the R54 rotamer in Ub^A^ was carried out in consideration of biochemical phenotypes caused by mutagenesis of E2287 and R54. However, future studies will be required to determine the precise location of these side chains. Even though the bonds of the applied probe were not visible in the density, we know the physical nature and geometry of the chemical probe. Thus, despite not being visible in the density, the three-way cross-link was built between UBR5 C2768, G75 of Ub^D^ and C48 of Ub^A^, as shown in Fig. 4a.

The final model was subjected to several iterations of real-space refinement in PHENIX^87^. Validation of the atomic model was performed using Molprobity^88^.

### Generation of di-Ubs and higher Ub chains

K27-, K29- and K33-linked di-Ubs were synthesized as described in the Supplementary Note. M1-linked di-Ub was expressed as a linear fusion in *E. coli*.

#### Enzymatic assembly of K6-, K11-, K48- and K63-linked di-Ub species

K6-, K11-, K48- and K63-linked di-Ubs were prepared using enzymatic assembly with tagless Ub.

Formation of K6-linked di-Ub was achieved by incubating 2.5 mM Ub with 0.1 μM E1, 0.6 μM UBE2L3, 10 μM NleL in the presence of 10 mM ATP in 40 mM Tris–HCl (pH 8.8), 10 mM MgCl_2_, 1 mM DTT for 3 h at 37 °C. The reaction was quenched with 10 mM DTT, and K48-linked Ub chains that were generated as a byproduct were removed by subsequent incubation with 2 μM OTUB1 for 3 h at 37 °C.

K11-linked Ub chains were obtained by incubating 0.5 mM Ub with 0.25 μM E1 and 5 μM Ube2S-UBA-IsoT^58^ in the presence of 10 mM ATP for 2 h at 37 °C.

To generate K48-linked di-Ub, 2.5 mM Ub was incubated with 1 μM E1 and 25 μM UBE2R1 in the presence of 10 mM ATP for 3 h at 37 °C. The reaction was quenched by adding 10 mM DTT and 1 μM associated molecule with the SH3 domain of STAM (AMSH).

K63-linked di-Ub was generated by incubating 1 mM Ub with 0.5 μM E1, 8 μM Ube2N and 8 μM Ube2V1 in 10 mM ATP, 40 mM Tris–HCl (pH 8.5), 10 mM MgCl_2_, 0.5 mM DTT at 37 °C for 30 min before stopping the reaction by addition of 10 mM DTT (final concentration).

Different chain lengths of the various chain types were then separated using iterative rounds of cation-exchange chromatography followed by size-exclusion chromatography in a final buffer of 25 mM HEPES (pH 7.5).

### Biochemical assays

Different migration properties on SDS–PAGE of various reaction products were used as read-out for all biochemical assays. After samples were collected and quenched with nonreducing or reducing (supplied with DTT at a final concentration of 100 mM) SDS–PAGE loading buffer, SDS–PAGE was performed. Fluorescent scanning was then conducted using the Amersham Imager 600. Intensity of the scans was increased, and the gels were cropped subsequently for figure preparation. After fluorescent scanning, each SDS–PAGE was stained with Coomassie Brilliant Blue to show protein inputs. All Coomassie-stained SDS–PAGE are shown in Supplementary Figs. 6 and 7. Raw, uncropped fluorescent images are available as Source data for Figs. 1, 2, 5, and Extended Data Figs. 1, 3, 4.

### Pulse-chase format: di-Ub synthesis assay

Di-Ub synthesis assays in a pulse-chase format were carried out to examine the effects of different UBR5 and Ub^A^ variants. The assays were performed with fluorescently labeled donor Ub (Ub^D^) and unlabeled acceptor Ub (Ub^A^). Unless stated otherwise, UBE2D2 was used as E2 as it showed higher reactivity toward UBR5 than UBE2L3 (ref. ^89^). In total, 20 μM of UBE2D2 was incubated with 30 μM fluorescent donor Ub^K48R^ (*Ub^D^) in the presence of 0.5 μM UBA1 in a buffer containing 25 mM HEPES (pH 7.5), 150 mM NaCl, 5 mM MgCl_2_, 2 mM ATP and 0.04 mg ml^−1^ BSA (pulse buffer) for 30 min at room temperature. Loading of the E2 was quenched with 50 mM ethylenediaminetetraacetic acid (EDTA).

During the chase reaction, UBR5 variants were mixed with distinct unlabeled acceptor Ub variants to test how well these can collaborate. Unless stated otherwise, the thioester-linked E2~*Ub^D^ was diluted into a mix of E3 (0.2 μM final) and the respective Ub^A^-6xHis (2 μM final) in 25 mM HEPES (pH 7.5), 150 mM NaCl and 1 mM DTT (chase buffer) to a final concentration of 0.2 μM. Samples were taken at the indicated times, and the reaction was quenched by adding nonreducing or reducing SDS–PAGE buffer.

#### UBR5 variants

Effects of E3 mutations were tested in the context of a WT UBR5 background. These include testing the effects of mutating the catalytic cysteine (Fig. 1b), the C-lobe–Ub^D^ interaction site A2790 (ref. ^33^; Fig. 2e), SDA^mut^ (Extended Data Fig. 3f), different variations of the C-terminal UBR5-tail (Fig. 5c), the UBA-variant L224D (Fig. 5d) and the LOL mutant (Fig. 5g). In addition to testing these various different UBR5 constructs, also different Ub^A^s were tested together with WT UBR5: in the Ub^D^-Ub^A^ interface (Fig. 5c), in the UBA-Ub^A^ interface (Fig. 5d), in the C-lobe-Ub^A^ interface (Fig. 5e), or in the N-lobe-Ub^A^ interface (Fig. 5f). Pulse reactions were followed by chase reactions with the respective UBR5 variants or different mutations of Ub^A^-6xHis.

Samples were taken at the indicated time points and mixed with nonreducing SDS–PAGE loading buffer. In the case of different Ub^A^ variants in the UBR5 UBA domain interface with Ub^A^ interface, the samples were taken after 1 min.

#### Autoubiquitylation

To address intrinsic activity, the extent of UBR5 autoubiquitylation was examined using a pulse-chase assay. Subsequently, to a pulse reaction, E2~*Ub^D^ was added to UBR5^WT^ or UBR5^WT^ premixed with WT Ub^A^-6xHis (Extended Data Fig. 1a). Samples were taken at the indicated time points, and nonreducing and reducing SDS–PAGE was performed.

#### UBR5 truncations

UBR5 truncations were also tested for their ability to form di-Ubs (Fig. 1h). The constructs used during the chase reaction comprised either WT UBR5, UBR5^Dimer^ (UBR5 L710D) or truncations: the entire C-terminal HECT domain including the inserted MLLE domain (HECT), or with the MLLE domain replaced by a structure-based six amino acid long linker (HECT^ΔMLLE^), or the isolated UBA domain (residues 179–230) connected to the complete HECT domain with the MLLE domain (residues 2377–2454, the resultant protein referred to as ‘UBA–HECT’) by a 15 amino acid long linker ((GGGSS)_3_)_._ To test these constructs, the respective E3 variant (0.5 μM final concentration) was mixed with tagless Ub^A^ or tagless Ub^A^ harboring a K48R variant (5 μM final concentration) and 1 μM UBE2D2~*Ub^D^. Samples were taken after 0.3, 2 and 5 min and mixed with nonreducing SDS–PAGE loading buffer.

#### Determination of linkage-specificity

To test the linkage-specificity of UBR5 and UBR5^Dimer^, the chase reaction was performed by mixing UBR5 or UBR5^Dimer^ with untagged acceptor Ub containing either all lysines (WT), no lysines (all lysines were mutated to arginines = ‘K0’), or only one distinct lysine (6, 11, 27, 29, 33, 48 or 63), and all other lysines were mutated to arginines. Samples were taken after 1 min and quenched with reducing SDS–PAGE buffer (Extended Data Fig. 1f).

#### Assay testing E2 mutation at interface with UBR5 N-lobe

To test whether mutating the E2 in the E2-N-lobe interface would affect the di-Ub-formation, the point mutation F62A^60^ of UBE2D3 and UBE2D3 WT were used during the pulse reaction (Fig. 2d). In total, 20 μM of the respective E2 was incubated with 30 μM fluorescent donor Ub^K48R^ and 0.5 μM UBA1 in the pulse buffer for 30 min at room temperature. Loading of the E2 was quenched with 50 mM EDTA. Subsequently, 0.2 μM E2~*Ub^D^ was added to a mix of 0.2 μM UBR5 and 2 μM Ub^A^-6xHis. Samples were taken at the indicated times and quenched with reducing and nonreducing SDS–PAGE loading buffer.

#### Testing potential pH influence on LOL mutant

Whether a varying pH influences the Ub-chain-forming activity of UBR5 was also addressed using the di-Ub synthesis assay^90^ (Extended Data Fig. 4d). The pulse reaction was performed in pH 7.5 as before so as to not influence E2-loading. Instead of performing the chase reaction in 25 mM HEPES (pH 7.5), 150 mM NaCl and 1 mM DTT, different buffer components were now used. For the reactions performed at pH 6.8, 7.5, 8.0 and 8.8, 25 mM Tris–HCl was adjusted to the respective pH. The remaining components, 150 mM NaCl and 1 mM DTT, were maintained in all reactions, regardless of the pH. Reactions at higher pH required different buffers, pH 9.5 was achieved using CAPSO and pH 11 was realized using CAPS. In total, 0.2 μM UBR5 or UBR5^D2283-2287A^ (LOL^mut^) was mixed with 2 μM Ub^A^-6xHis in the respective buffer. E2~*Ub^D^ was diluted into this sample by a ratio of 1:20 to not affect the pH substantially. Samples were taken after 60 s and nonreducing SDS–PAGE was performed.

### Pulse-chase format: tri-Ub synthesis assay

To test how well UBR5 can modify differently linked di-Ubs and to see whether this correlates with the accessibility of the respective lysine on Ub^A^ observed in the TS2 structure, a tri-Ub synthesis assay was performed (Fig. 5h). E2~*Ub^D^, generated in a pulse reaction, was mixed with UBR5 or UBR5^Dimer^ and di-Ub linked via the indicated lysine (2 μM final concentration). Samples were taken after 20 s and mixed with reducing SDS–PAGE loading buffer.

### Pulse-chase format: free Ub chain formation assay

Examination of polyubiquitylation activity of UBR5 and UBR5^Dimer^ (Extended Data Fig. 1g) was performed using a pulse-chase assay with fluorescein-labeled WT Ub that could serve as both donor and acceptor. The pulse reaction was performed by incubating 30 μM labeled Ub with 20 μM UBE2D2 and 0.5 μM E1 in pulse buffer for 30 min at room temperature. The reaction was stopped by the addition of 50 mM EDTA, and the chase reaction was performed by mixing 0.2 μM of the indicated UBR5 version with 1 μM E2~*Ub^D^. Samples were taken at the indicated time points and mixed with reducing SDS-loading buffer.

### Multiturnover format: polyubiquitylation

To test the polyubiquitylation activity of the SDA mutant UBR5^H1362-1364D^ (Fig. 2i), a multiturnover assay was performed. For this, 20 μM fluorescein-labeled WT Ub was mixed with 5 μM UBE2D2 and 0.5 μM of WT UBR5 or the SDA mutant in the pulse buffer. Addition of 0.5 μM E1 started the reaction, and samples were taken at the indicated time points, mixed with reducing SDS-loading buffer.

### Reporting summary

Further information on research design is available in the Nature Portfolio Reporting Summary linked to this article.

## Online content

Any methods, additional references, Nature Portfolio reporting summaries, source data, extended data, supplementary information, acknowledgements, peer review information; details of author contributions and competing interests; and statements of data and code availability are available at 10.1038/s41589-023-01414-2.

### Supplementary information

Supplementary Information Supplementary Tables 1–3, Supplementary Figs. 1–7, Supplementary Videos 1–3, Supplementary Note and Supplementary References.

Reporting Summary

Supplementary Video 1 Three-dimensional (3D)-VA analysis of UBR5^Dimer^~Ub^D^ showing conformational flexibility of C-lobe~Ub^D^ moiety.

Supplementary Video 2 Ubiquitylation cascade of UBR5^Dimer^.

Supplementary Video 3 Different transition states modeled in tetrameric UBR5 showing compatibility of all transition states with tetrameric oligomeric state.

### Source data

Source Data Fig. 1 Unprocessed, uncropped SDS–PAGE.

Source Data Fig. 2 Unprocessed, uncropped SDS–PAGE.

Source Data Fig. 5 Unprocessed, uncropped SDS–PAGE.

Source Data Extended Data Fig. 1 Unprocessed, uncropped SDS–PAGE.

Source Data Extended Data Fig. 3 Unprocessed, uncropped SDS–PAGE.

Source Data Extended Data Fig. 4 Unprocessed, uncropped SDS–PAGE.

## Data Availability

The structural data will be available from EMDB and RCSB upon manuscript publication (UBR5^Dimer^ EMDB-16355, PDB 8C06; UBR5^Dimer^~Ub^D^~Ub^A^ EMDB-16356, PDB 8C07). Cryo-EM maps were deposited and are available via the following accession number: UBR5^C2768A^ EMDB-16865, UBE2D2~Ub^D^~UBR5^Dimer^ EMDB-16867, UBR5~Ub^D^ EMDB-16866, UBR5^Dimer^~Ub^D^~Ub^A^ (global map) EMDB-17466. Raw gel images are provided as source data. Coomassie-stained SDS–PAGE of all assays are added as Supplementary Figs. 6 and 7. Accession codes of other (published) data used for comparison are as follows: PDB: 3JVZ, PDB: 4LCD, PDB: 2QHO, PDB: 6XZ1, PDB: 3NY3, PDB: 4BBN, EMD-28646. There is no restriction on data availability. Source data are provided with this paper.
