# Supplementary material for: Structural snapshots along K48-linked ubiquitin chain formation by the HECT E3 UBR5
Source: Nat Chem Biol. 2023 Aug 24;20(2):190–200. doi: 10.1038/s41589-023-01414-2 (PMC10830417; doi:10.1038/s41589-023-01414-2)
Supplement: Supplementary file 1 — Supplementary Tables 1–3, Supplementary Figs. 1–7, Supplementary Videos 1–3, Supplementary Note and Supplementary References. [file 41589_2023_1414_MOESM1_ESM.pdf]

nature chemical biology

Article

https://doi.org/10.1038/s41589-023-01414-2

Structural snapshots along K48-linked ubiquitin chain formation by the HECT E3 UBR5

In the format provided by the authors and unedited

Supplementary Information

Table of Contents

Supplementary Table 1:
Cryo-EM data collection, refinement, and validation statistics

Supplementary Figures
Supplementary Figure 1: Cryo-EM processing scheme of UBR5^{C2768A}
Supplementary Figure 2: Cryo-EM processing scheme of UBR5^{Dimer}
Supplementary Figure 3: Cryo-EM processing scheme of stable mimic representing UBR5~Ub^{D}~E2
Supplementary Figure 4: Cryo-EM processing scheme of stable mimic representing UBR5~Ub^{D}
Supplementary Figure 5: Cryo-EM processing scheme of stable mimic representing K48-linked Ub chain formation by UBR5
Supplementary Figure 6: Coomassie stained assays showing protein inputs
Supplementary Figure 7: Coomassie stained assays showing protein inputs

Supplementary videos
Supplementary video 1: 3D-VA analysis of UBR5^{Dimer}~Ub^{D} showing conformational flexibility of C-lobe~Ub^{D} moiety
Supplementary video 2: Ubiquitylation cascade of UBR5^{Dimer}
Supplementary video 3: Different transition states modelled in tetrameric UBR5, showing compatibility of all transition states with tetrameric oligomeric state.

Supplementary Note
Supplementary Table 2: Oligonucleotides used for construct design
Supplementary Table 3: Constructs employed for recombinant protein expression in the respective system.

General Procedures for Chemical Synthesis
Chemical synthesis of Ub-VME and Rhodamine-Ub-VME
Chemical synthesis of K27, K29 and K33-linked di-ubiquitin species

References

Supplementary Table 1: Cryo-EM data collection, refinement, and validation statistics

	Apo Tetramer (EMDB-16865)	Apo Dimer (EMDB-16355) (PDB 8C06)	E2-Ub-bound Dimer (EMDB-16867)	UbVME-bound Dimer (EMDB-16866)	K48-DiUb-bound Dimer (EMDB-17466)	K48-DiUb-bound Dimer (EMDB-16356) (PDB 8C07)
**Data collection and processing**						
Magnification	64,000	105,000	73,000	22,000	22,000	105,000
Voltage (kV)	300	300	200	200	200	300
Electron exposure (e–/Å^2)	56.3	67.8	70	60	60	69
Defocus range (μm)	-2.4 - -0.8	-3.0- -0.5	-3.5- -1.0	-2.6- -0.8	-3.0- -0.3	-2.2 - -0.6
Pixel size (Å)	1.384	0.8512	1.997	1.885	1.885	0.8512
Symmetry imposed	C2	C2	C1	C1	C1	C1
Initial particle images (no.)	1,122,292	762,722	1,381,245	834,722	306,268	1,708,682
Final particle images (no.)	148,825	226,919	46,615	197,281	42,761	141,034
Map resolution (Å) FSC threshold	3.7 (0.143)	2.7 (0.143)	7.3 (0.143)	5.3 (0.143)	8.3 (0.143)	3.3 (0.143)
Map resolution range (Å)	3.3-9.8	2.6-5.1	5.6-25.1	4.7-18.2	7.54-21.6	3.2-10
**Refinement**						
Initial model used (PDB code)		AlphaFold2				8C06
Model resolution (Å)		2.7				3.3
Model						
composition		25332				4974
Non-hydrogen		3298				644
atoms		6				1
Protein residues						
Ligands						
B factors (Å^2)						
Protein		62				81.2
Ligand		52				84
R.m.s.						
deviations		0.003				0.004
Bond lengths		0.765				0.944
(Å)						
Bond angles (°)						
Validation						

MolProbity	1.4	1.7
score	2.3	4.1
Clashscore	0	0
Poor rotamers		
(%)		
Ramachandran		
plot	95	92
Favored (%)	5	8
Allowed (%)	0	0
Disallowed		
(%)		

Supplementary Figures

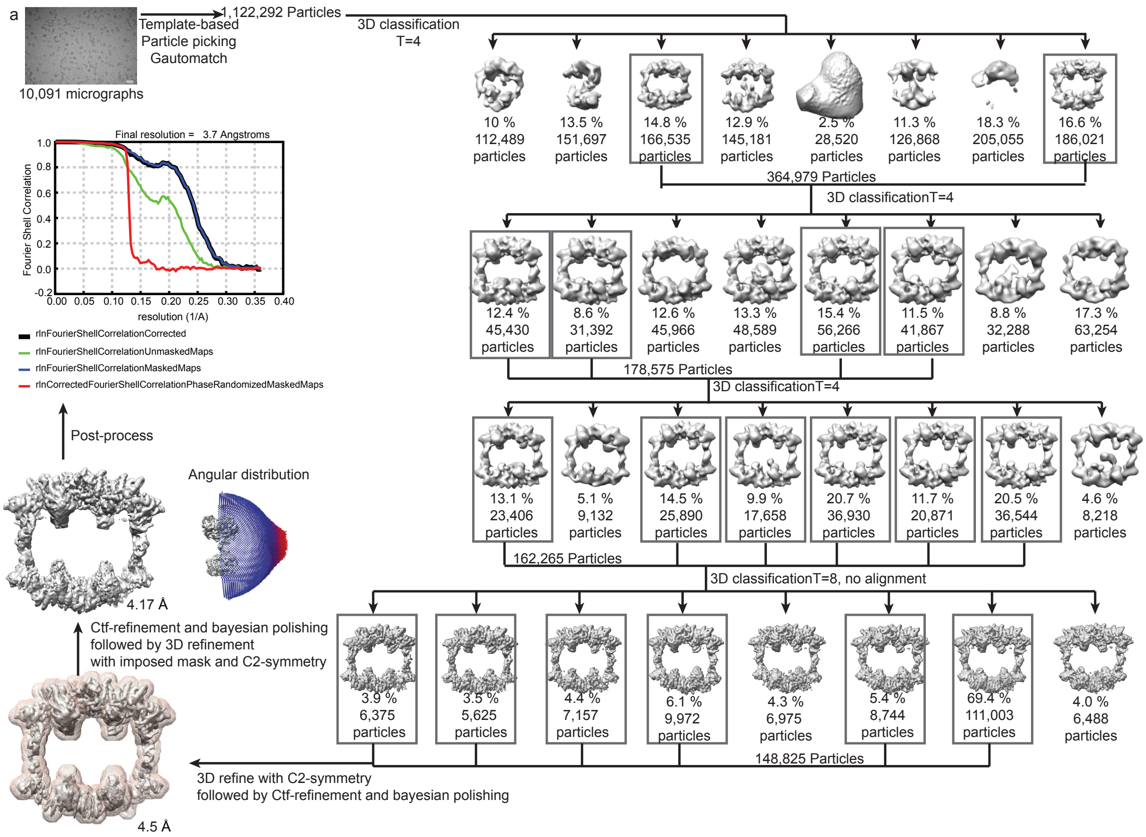

Supplementary Figure 1: Cryo-EM processing scheme of UBR5^{C2768A}

Cryo-EM processing schematic of UBR5^{C2768A}. Scalebar on micrograph corresponds to 500 Å. The micrograph is representative for the collected 10,091 micrographs of this dataset. Data processed in RELION 3.1.1[75] yielded a 3D reconstruction with a resolution of 3.7 Å by the gold-standard Fourier shell correlation of 0.143.

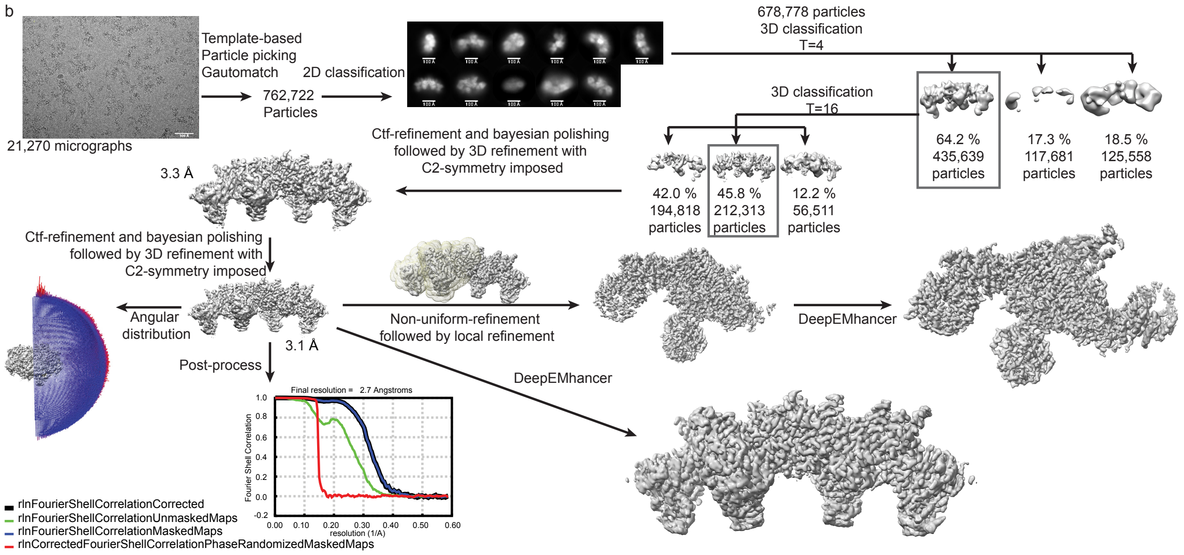

Supplementary Figure 2: Cryo-EM processing scheme of UBR5^{Dimer}

Cryo-EM processing scheme of UBR5^{Dimer}. Scalebar on micrograph corresponds to 500 Å. The micrograph is representing the collected 21,270 micrographs of this dataset. Scalebars on 2D classes correspond to 100Å. Mask imposed for local refinement subsequently to unmasked non-uniform refinement is shown in yellow transparent surface. Data processing was performed in RELION 4.0 followed by processing in CryoSparc4.2.0 for non-uniform refinement and local refinement. A final 3D reconstruction with a resolution of 2.7 Å by the gold-standard Fourier shell correlation of 0.143 and a local refined reconstruction of 3 Å was achieved.

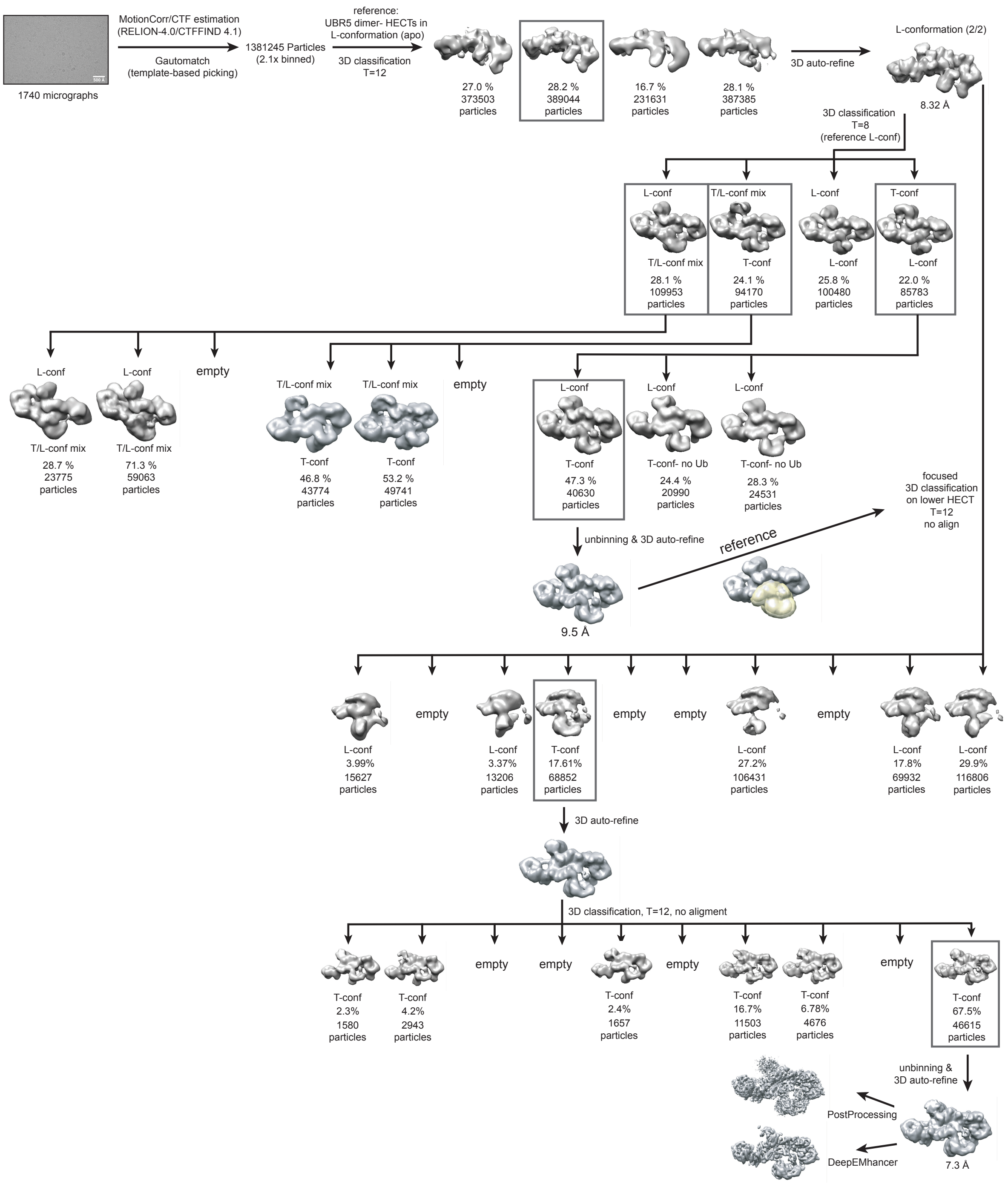

Supplementary Figure 3: Cryo-EM processing scheme of stable mimic representing UBE2D2~Ub^{D}~UBR5^{Dimer}

Cryo-EM processing schematic of UBE2D2~Ub^{D}~UBR5^{Dimer}. Scalebar on micrograph corresponds to 500 Å. The depicted micrograph is representative for the collected 1,740 micrographs of this dataset. Mask used for focused 3D-classification is shown in yellow transparent surface. Data processed in RELION 4.0 yielded a final 3D reconstruction with a resolution of 7.3 Å by the gold-standard Fourier shell correlation of 0.143.

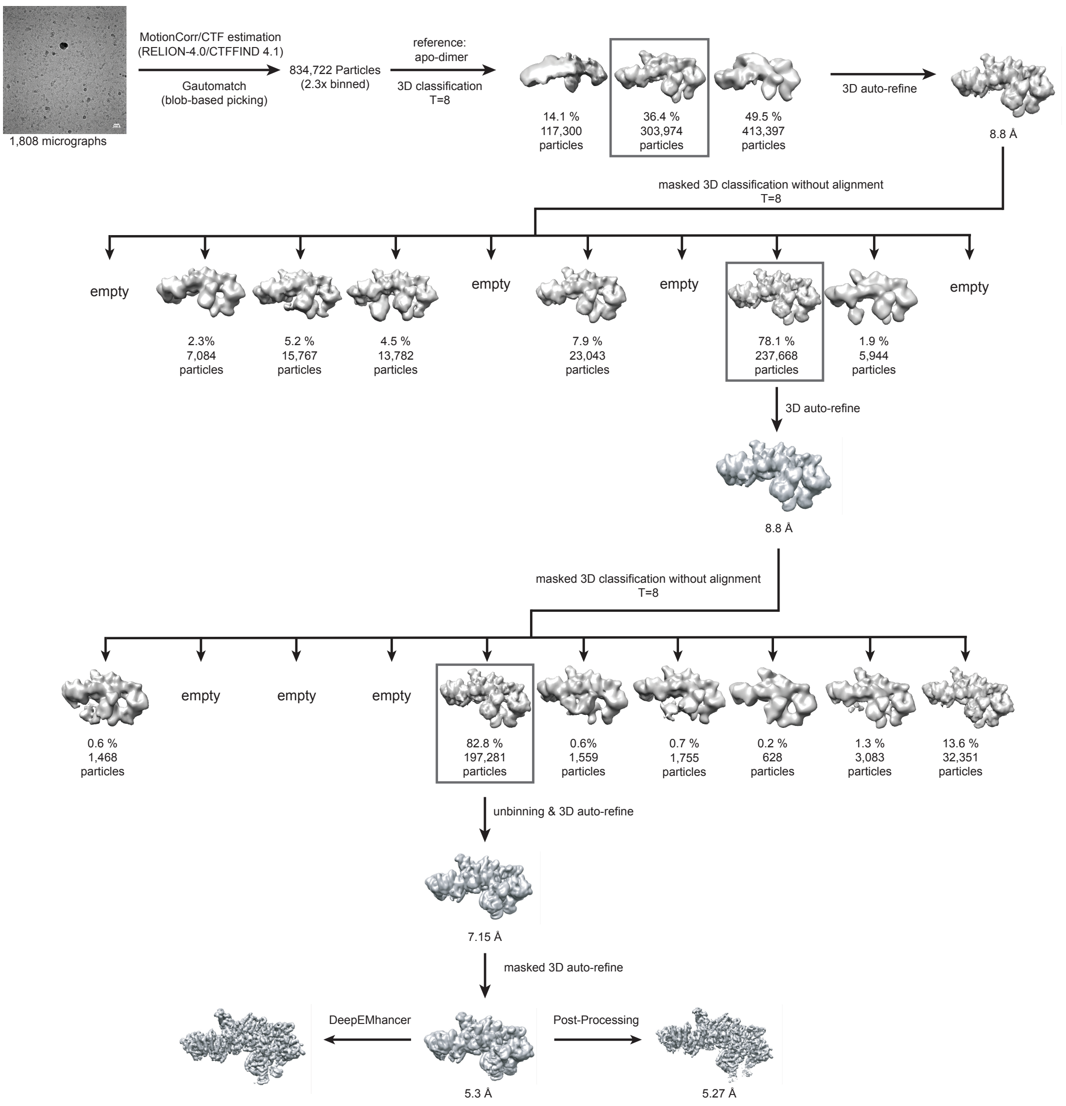

Supplementary Figure 4: Cryo-EM processing scheme of stable mimic representing UBR5~Ub^{D}

Cryo-EM processing schematic of UBR5^{Dimer}~Ub^{D}. A scalebar on the micrograph representative for the collected 1,808 micrographs depicts 300 Å. Data processing was performed using RELION 4.0 and yielded a final 3D reconstruction with a resolution of 5.3 Å by the gold-standard Fourier shell correlation of 0.143.

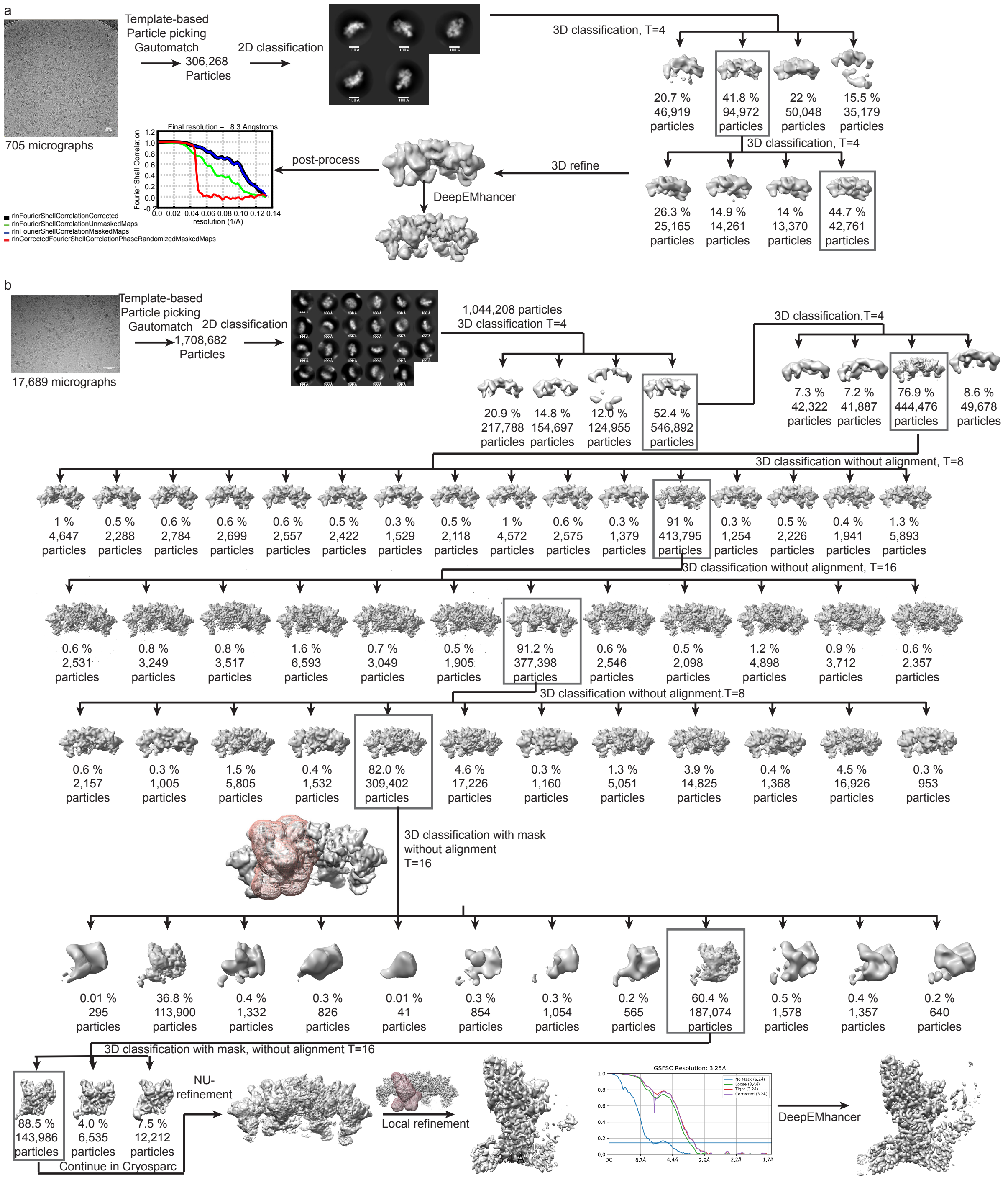

Supplementary Figure 5: Cryo-EM processing scheme of stable mimic representing K48-linked Ub chain formation by UBR5

a, Cryo-EM processing schematic of UBR5^{Dimer}~Ub^{D}~Ub^{A} complex for global map. Scalebar on micrograph corresponds to 300 Å. The micrograph represents the 705 collected micrographs of this dataset. Scalebars on 2D classes correspond to 100Å. Data processed with RELION 3.1.1 with final resolution of 8.3 Å by the gold-standard Fourier shell correlation of 0.143. **b,** Cryo-EM processing schematic of UBR5^{Dimer}~Ub^{D}~Ub^{A} complex. Micrograph represents the 17,689 collected micrographs and the scalebar corresponds to 500 Å. Scalebars on 2D classes correspond to 100Å. Masks used at various steps of focused 3D classification and 3D refinement are shown in pink transparent surfaces. Data was processed with RELION 3.1.1 until focused 3D classification. Non-uniform refinement and focused 3D refinement were performed using Cryosparc with a final 3D reconstruction 3.4 Å resolution by the gold-standard Fourier shell correlation of 0.143.

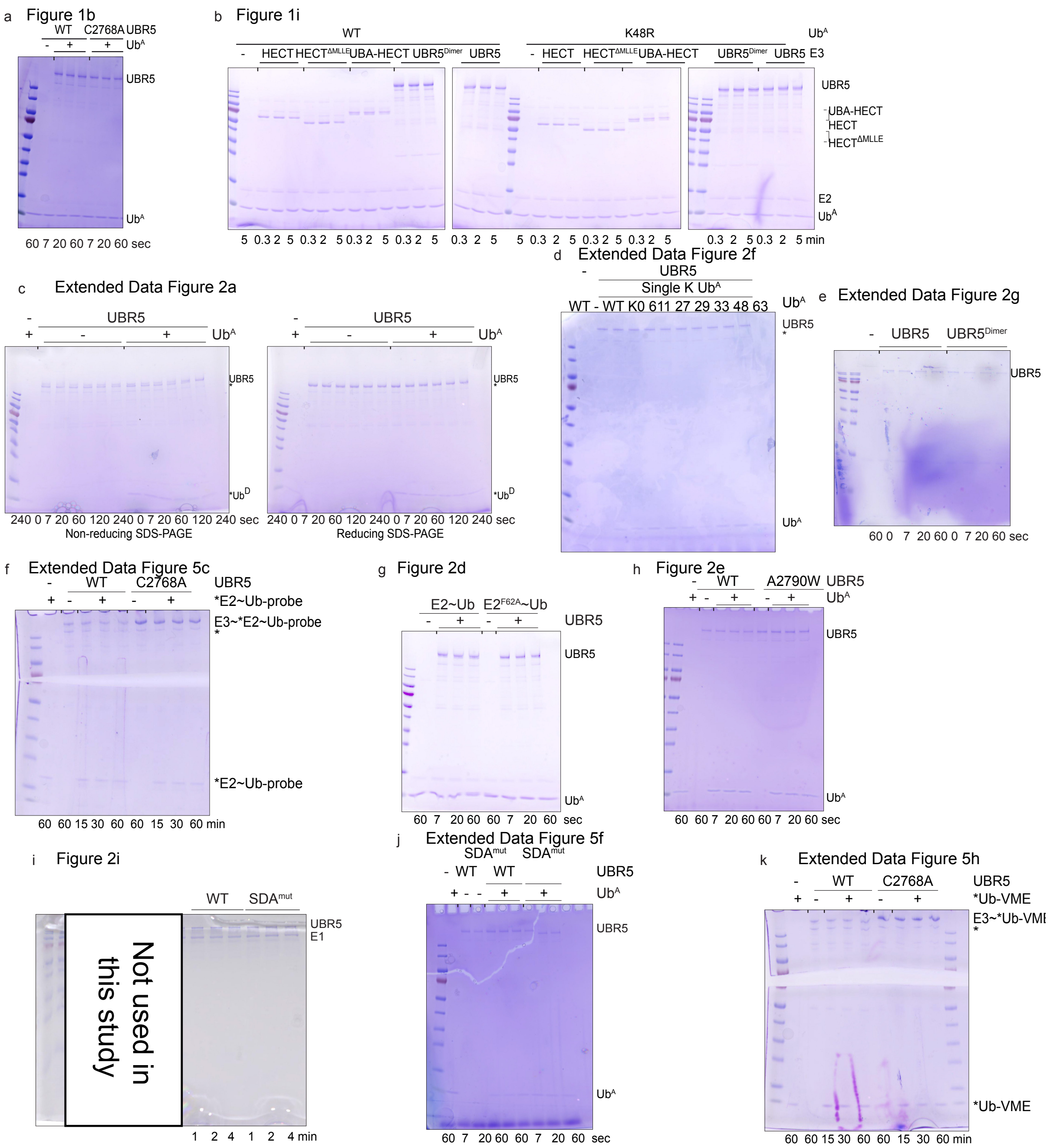

Supplementary Figure 6: Coomassie stained assays showing protein inputs. The order the assays are discussed in the text is maintained.

a, Coomassie-stain of Di-ubiquitin synthesis assay in Figure 1b**. b,** Coomassie-stain of Di-ubiquitin synthesis assay in Figure 1i**. c,** Coomassie-stain of Di-ubiquitin synthesis assay in Extended Data Figure 1a**. d,** Coomassie-stain of Di-ubiquitin synthesis assay in Extended Data Figure 1f **. e,** Coomassie-stain of Di-ubiquitin synthesis assay in Extended Data Figure 1g**. f,** Coomassie-stain of Di-ubiquitin synthesis assay in Extended Data Figure 3c**. g,** Coomassie-stain of Di-ubiquitin synthesis assay in Figure 2d**. h,** Coomassie-stain of Di-ubiquitin synthesis assay in Figure 2e**. i,** Coomassie-stain of Di-ubiquitin synthesis assay in Figure 2i**. j,** Coomassie-stain of Di-ubiquitin synthesis assay in Extended Data Figure 3f**. k,** Coomassie-stain of Di-ubiquitin synthesis assay in Extended Data Figure 3h

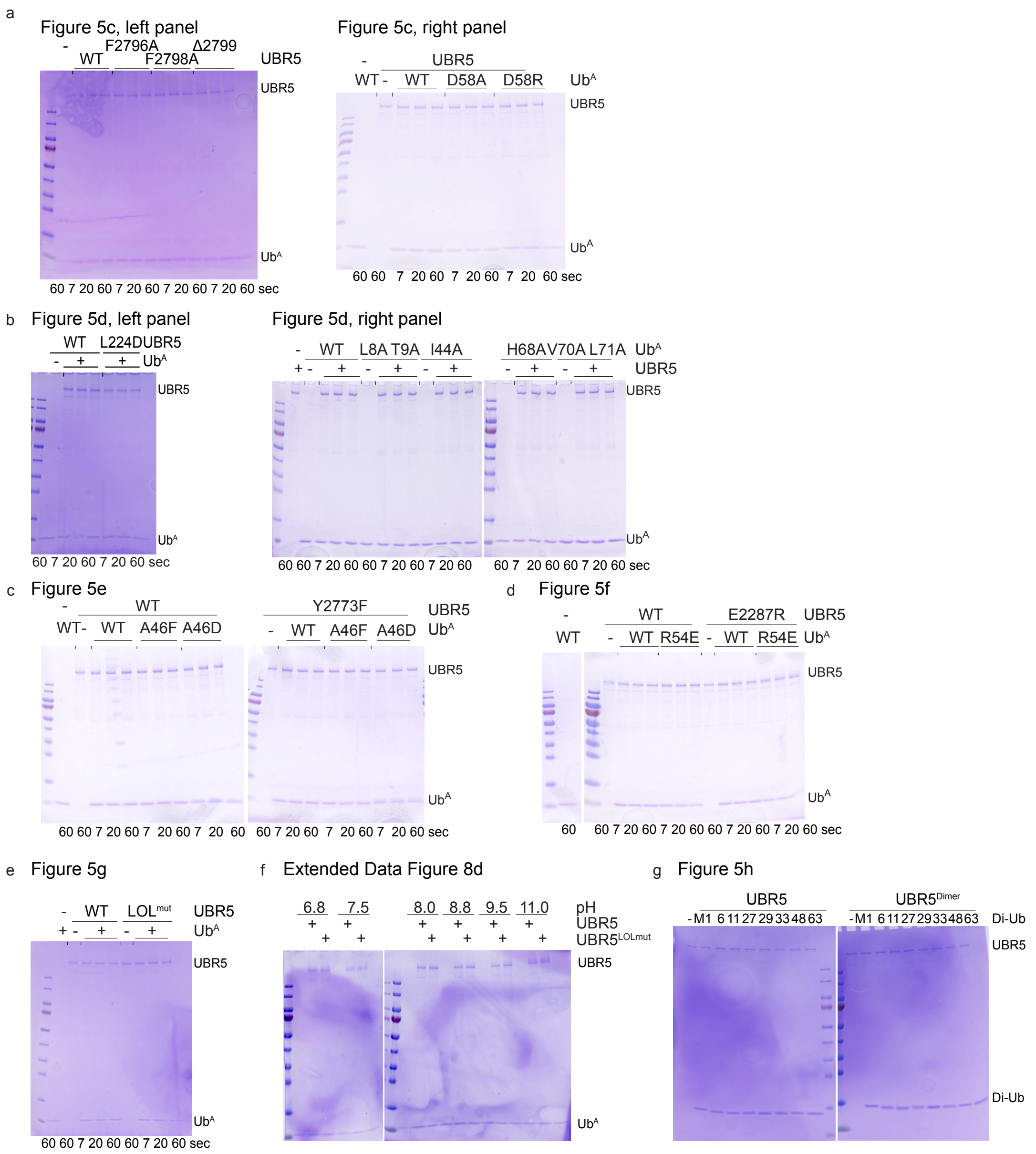

Supplementary Figure 7: Coomassie stained assays showing protein inputs.
a, Coomassie-stain of Di-ubiquitin synthesis assay in Figure 5c. **b,** Coomassie-stain of Di-ubiquitin synthesis assay in Figure 5d. **c,** Coomassie-stain of Di-ubiquitin synthesis assay in Figure 5e. **d,** Coomassie-stain of Di-ubiquitin synthesis assay in Figure 5f. **e,** Coomassie-stain of Di-ubiquitin synthesis assay in Figure 5g. **f,** Coomassie-stain of Di-ubiquitin synthesis assay in Extended Data Figure 4d. **g,** Coomassie-stain of Di-ubiquitin synthesis assay in Figure 5h

Supplementary videos

Supplementary Video 1: 3D-VA analysis of $UBR5^{Dimer}$~Ub^{D} showing conformational flexibility of C-lobe~Ub^{D} moiety

Supplementary Video 2: Ubiquitylation cascade of $UBR5^{Dimer}$

Supplementary Video 3: Different transition states modelled in tetrameric UBR5, showing compatibility of all transition states with tetrameric oligomeric state.

Supplementary Note

Supplementary Table 2: Oligonucleotides used for construct design

Cloning strategy	Name	Sequence (5' → 3')
Gibson assembly	UBR5 into pEG 2xStrep-GFP-3C	GATCCGGAAGCGGCGCGCCC ATGACGTCCATCCATTTCGTG
Gibson assembly	UBR5 into pEG 2xStrep-GFP-3C	GATTCGAAAGCGGCCGCTTATTAGGGCCCCTACACAA AACCAAAATTCTTGGTC
Gibson assembly	Linearize pEG 2xStrep-GFP-3C	GGGCGCGCCGCTTCCGGATC
Gibson assembly	Linearize pEG 2xStrep-GFP-3C	TAGgggcccTAATAAgcggccgctttcg
Gibson assembly	Linearize pFLN GST-TEV	AGAGCCACCGGATCCC
Gibson assembly	Linearize pFLN GST-TEV	TAAgaattcaaaggcctacgtcg
Gibson assembly	UBR5 HECT into pFLN GST-TEV	GGGATCCGGTGGCTCT TTTGAGGTAAAAGAATCAAAATTCCGC
Gibson assembly	UBR5 HECT into pFLN GST-TEV	GGGATCCGGTGGCTCT ATTCCGGCTTCCGTCATC
Gibson assembly	UBA into pFLN GST-TEV	GGGATCCGGTGGCTCT ATTCCGGCTTCCGTCATC
Gibson assembly	Linearize pFLN GST-TEV-HECT	CTAGTGGCGGTGGAAGTTCA GGTGGTTTTGAGGTAAAAGAATCA
Quickchange mutagenesis	UBR5 HECT Δ2377-2454 (ΔMLLE)	cgacactaggccctttaga GGAGGTAGTGGTGGTTCT gaaaatggagctgatagta
Quickchange mutagenesis	UBR5 HECT Δ2377-2454 (ΔMLLE)	tactatcagctccattttcAGAACCACCACTACCTCCtctaaagggcct agtgtcg
Quickchange mutagenesis	UBR5 C2768A	gggacgtaaagtcgagaaatggcagtatttgcagtaggaagatg
Quickchange mutagenesis	UBR5 C2768A	catcttcctactgcaaatactgccatttctcgactttacgtccc
Quickchange mutagenesis	UBR5 L224D	ttcatcatcccggctatctaaattatttacagcaaggttcacatcaag
Quickchange mutagenesis	UBR5 L224D	cttgatgtgaaccttgctgtaaataatttagatagccgggatgatgaa
Quickchange mutagenesis	UBR5 L710D	gatgctgacccttcttctctcgatcaggattgtaggttacttaga
Quickchange mutagenesis	UBR5 L710D	cttctctcctgcaggattgtagggatcttagaattgatgaattgcaggt
Quickchange mutagenesis	UBR5 E2283-2288A	aaaaacttcgtgctacaccactggccgctgctgccgcagccttaaatgtgactttt actctg
Quickchange mutagenesis	UBR5 E2283-2288A	cagagtaaaagtcacatttaaggctgcggcagcagcggccagtggtgtagcac gaagttttt
Quickchange mutagenesis	UBR5 A2790W	cacaaaaccaaaattcttggtcttaatccagagtaacaatttctgtttgagaatctg
Quickchange mutagenesis	UBR5 A2790W	cacaaaaccaaaattcttggtcttaatccagagtaacaatttctgtttgagaatctg
Quickchange mutagenesis	UBR5 HLL1362-1364DDD	ctgattgaggtatacttgctcttctggatcatcatcgcctattctactgctggcactaa gagg

Quickchange mutagenesis	UBR5 HLL1362-1364DDD	cctcttagtgccagcagtagaataggcgatgatgatccagaagagcaagtata cctcaatcag
Quickchange mutagenesis	UBR5 Y2773F	atagagtgggacgaaaagtcgagaaatgcaagtatttgc
Quickchange mutagenesis	UBR5 Y2773F	gcaaatacttgcatttctcgacttttcgtcccactctat
Quickchange mutagenesis	UBR5 F2796A	attactacacaaaaccagcattcttggtcttaatggcgagtaacaatttctg
Quickchange mutagenesis	UBR5 F2796A	cagaaattgttactcgccattaagaccaagaatgctggttttgtgtagtaat
Quickchange mutagenesis	UBR5 F2798A	ggccgcttattactacacagcaccaaaattcttggtcttaatggcg
Quickchange mutagenesis	UBR5 F2798A	cgccattaagaccaagaattttggtgctgtgtagtaataagcggcc
Quickchange mutagenesis	UBR5 Δ2799	cgaaagcggccgcttattactactaaaaaccaaaattcttggtcttaat
Quickchange mutagenesis	UBR5 Δ2799	attaagaccaagaattttggtttttagtagtaataagcggccgctttcg
Quickchange mutagenesis	UBR5 E2287R	tgctacaccactgccccttcctggctcatccttaaatg
Quickchange mutagenesis	UBR5 E2287R	catttaaggatgagccaggaaggggcagtggtgtagca
Quickchange mutagenesis	Ub^{A}-6xHis D58A	gactccttttgaatattgtaggcagacaaagtacgtccatctt
Quickchange mutagenesis	Ub^{A}-6xHis D58A	aagatggacgtactttgtctgcctacaatattcaaaaggagtc
Quickchange mutagenesis	Ub^{A}-6xHis D58R	agactccttttgaatattgtagcgagacaaagtacgtccatcttcc
Quickchange mutagenesis	Ub^{A}-6xHis D58R	ggaagatggacgtactttgtctcgctacaatattcaaaaggagtct
Quickchange mutagenesis	Ub^{A}-6xHis I44A	gctgcttgccagcaaaggccagtctctgctgatcag
Quickchange mutagenesis	Ub^{A}-6xHis I44A	ctgatcagcagagactggcctttgctggcaagcagc
Quickchange mutagenesis	Ub^{A}-6xHis H68A	gcacgaagtctcaacacaagagcaagagtagactccttttgaatatt
Quickchange mutagenesis	Ub^{A}-6xHis H68A	aatattcaaaaggagtctactcttgctcttgtgttgagacttcgtgc
Quickchange mutagenesis	Ub^{A}-6xHis A46F	ccagctgcttgccaaaaaagatcagtctctgctgatcagga
Quickchange mutagenesis	Ub^{A}-6xHis A46F	tcctgatcagcagagactgatcttttttggcaagcagctgg
Quickchange mutagenesis	Ub^{A}-6xHis A46D	ttccagctgcttgccatcaaagatcagtctctgc
Quickchange mutagenesis	Ub^{A}-6xHis A46D	gcagagactgatctttgatggcaagcagctggaa
Quickchange mutagenesis	Ub^{A}-6xHis R54E	tattgtagtcagacaaagtctctccatcttccagctgcttgccag
Quickchange mutagenesis	Ub^{A}-6xHis R54E	ctggcaagcagctggaagatggagagactttgtctgactacaata

Supplementary Table 3: Constructs employed for recombinant protein expression in the respective system.

Protein	Construct	Vector	Expression system
E1	GST-TEV-UBA1	pLIB	High-Five insect cells
E2	GST-TEV-UBE2D2	pGEX	BL21(DE3) RIL cells
	GST-TEV-UBE2D3	pGEX	BL21(DE3) RIL cells
	GST-TEV-UBE2D3^{F62A}	pGEX	BL21(DE3) RIL cells
Donor ubiquitin	GST-3C-Cys-Ub	pGEX	BL21(DE3) RIL cells

	GST-3C-Cys-Ub^{K48R}	pGEX	BL21(DE3) RIL cells
Single K ubiquitin	GST-3C-Ub	pGEX	BL21(DE3) RIL cells
	GST-3C-Ub^{K0}	pGEX	BL21(DE3) RIL cells
	GST-3C-Ub^{K6only}	pGEX	BL21(DE3) RIL cells
	GST-3C-Ub^{K11only}	pGEX	BL21(DE3) RIL cells
	GST-3C-Ub^{K27only}	pGEX	BL21(DE3) RIL cells
	GST-3C-Ub^{K29only}	pGEX	BL21(DE3) RIL cells
	GST-3C-Ub^{K33only}	pGEX	BL21(DE3) RIL cells
	GST-3C-Ub^{K48only}	pGEX	BL21(DE3) RIL cells
	GST-3C-Ub^{K63only}	pGEX	BL21(DE3) RIL cells
M1-linked Di-Ub	GST-TEV-Ub$_2$	pGEX	BL21(DE3) RIL cells
Acceptor ubiquitin	Ub(1-76)-6xHis	pRSF	BL21(DE3) RIL cells
	Ub(1-76)^{L8A,T9A}-6xHis	pRSF	BL21(DE3) RIL cells
	Ub(1-76)^{I44A}-6xHis	pRSF	BL21(DE3) RIL cells
	Ub(1-76)$^{V70A\ L71A}$-6xHis	pRSF	BL21(DE3) RIL cells
	Ub(1-76)^{H68A}-6xHis	pRSF	BL21(DE3) RIL cells
	Ub(1-76)^{R54E}-6xHis	pRSF	BL21(DE3) RIL cells
	Ub(1-76)^{A46F}-6xHis	pRSF	BL21(DE3) RIL cells
	Ub(1-76)^{A46D}-6xHis	pRSF	BL21(DE3) RIL cells
	Ub(1-76)^{D58A}-6xHis	pRSF	BL21(DE3) RIL cells
	Ub(1-76)^{D58R}-6xHis	pRSF	BL21(DE3) RIL cells
	Ub	pET22b	BL21(DE3) RIL cells
	Ub^{K48R}	pET22b	BL21(DE3) RIL cells
HECT domain	GST-TEV-HECT^{withMLLE}	pFLN	High-Five insect cells
	GST-TEV-HECT$^{\Delta MLLE}$	pFLN	High-Five insect cells
	GST-TEV-UBA-(GSG)$_5$-HECT	pFLN	High-Five insect cells
UBR5-mutants	TwinStrep-GFP-3C-UBR5	pEG	HEK293S, BacMam-system
	TwinStrep-GFP-3C-UBR5^{C2768A}	pEG	HEK293S, BacMam-system
	TwinStrep-GFP-3C-UBR5^{L710D} (UBR5$^{Dimer)}$	pEG	HEK293S, BacMam-system
	TwinStrep-GFP-3C-UBR5$^{HLL1362-1364DDD}$ (SDA^{mut})	pEG	HEK293S, BacMam-system
	TwinStrep-GFP-3C-UBR5^{A2790W}	pEG	HEK293S, BacMam-system
	TwinStrep-GFP-3C-UBR5^{L224D}	pEG	HEK293S, BacMam-system
	TwinStrep-GFP-3C-UBR5$^{D2283-2287A}$ (LOL^{mut})	pEG	HEK293S, BacMam-system
	TwinStrep-GFP-3C-UBR5^{E2287R}	pEG	HEK293S, BacMam-system
	TwinStrep-GFP-3C-UBR5^{F2796A}	pEG	HEK293S, BacMam-system
	TwinStrep-GFP-3C-UBR5^{F2798A}	pEG	HEK293S, BacMam-system
	TwinStrep-GFP-3C-UBR5$^{\Delta 2799}$	pEG	HEK293S, BacMam-system
	TwinStrep-GFP-3C-UBR5^{Y2773F}	pEG	HEK293S, BacMam-system
Probe-constructs	His-Ub(1-75)-Intein	pET	BL21(DE3) RIL cells
	Ub^{K48C}	pET	BL21(DE3) RIL cells
	GST-TEV-UBE2D2^{C21A,C107A,C111S}	pGEX	BL21(DE3) RIL cells

General Procedures for Chemical Synthesis

General reagents were obtained from Sigma Aldrich, Acros and Fluka used as received. Solvents were purchased from Aldrich or BIOSOLVE. Peptide synthesis reagents were purchased from Novabiochem. LC-MS measurements were performed on a Waters Acquity

H-class UPLC with a LCT™ ESI-Mass Spectrometer. Samples were run using 2 mobile phases: A = 1 % CH_3CN, 0.1 % formic acid in water and B = 1 % water and 0.1 % formic acid in CH_3CN. Data processing was performed using Waters MassLynx Mass Spectrometry Software 4.1 (deconvolution with Maxent1 function).

Solid Phase Peptide Synthesis
SPPS was performed on a Syro II MultiSyntech Automated Peptide synthesizer using standard 9-fluorenylmethoxycarbonyl (Fmoc) based solid phase peptide chemistry at 25 µmol scale, using 4 x fold excess of amino acids relative to pre-loaded Fmoc amino acid trityl resin (0.2 mmol/g, Rapp Polymere GmbH).

RP-HPLC purifications
Waters preparative RP-HPLC system, equipped with a Waters C18-Xbridge 5 µm OBD (10 x 150 mm) column at a flowrate of 37.5 mL/min. using 3 mobile phases: A: MQ, B: CH_3CN and C: 1 % TFA in MQ. Prep-HPLC program: Gradient: 0 – 5 min: 5 % B, 5 % C; 5 – 7 min: 5 -> 20% B, 5% C; 7 – 18 min: 20 -> 45 % B, 5 % C. On a Waters C18-Xbridge 5 µm OBD (30 x 150 mm) column at a flowrate of 37.5 mL/min. Pure fractions were pooled and lyophilized.

Gel filtration
Size exclusion chromatography was performed on a Sephadex S75 10/300 column (GE Healthcare), using a 20 mM Tris-HCl, 150 mM NaCl buffer, pH 7.6. Appropriate fractions were pooled and concentrated using an Amicon spinfilter (MWCO 10 kDa) to a final concentration of 5.0 mg/mL.

(HR)-LC-MS-measurements
High resolution liquid chromatography mass analysis was performed on a Waters Acquity H-Class UPLC system equipped with a Waters ACQUITY Quaternary Solvent Manager(QSM) and Waters ACQUITY FTN AutoSampler. Separation was achieved on a Waters Acquity UPLC Protein BEH C4 column, 300Å, 1,7 uM (2.1 x 50 mm); flow rate = 0.6 mL/min , runtime = 4.55 min, column T = 60°C using 2 mobile phases: A = 0,1% formic acid in water and B = 0,1% formic acid in CH3CN. The products were analyzed by intact MS analysis (MS1) and masses were detected in a range from 550-2000 Da from 2.51 - 4.50 min and were recorded on a Waters XEVO-G2 XS Q-Tof mass spectrometer equipped with an electrospray ion source in positive mode (Capillary Voltage: 0.5 kV, desolvation gas flow: 900 L/h, desolvation gas temperature: 500°C, source temperature: 130 °C, probe angle: 9.5) with a resolution of R = 22,000. Data processing was performed using Waters MassLynx Mass Spectrometry Software 4.1 (deconvolution with MaxEnt1 function).

Chemical synthesis of Ub-VME and Rhodamine-Ub-VME
Ub(1-75) was synthesized on a Syro II MultiSyntech Automated Peptide synthesizer using standard 9-fluorenylmethoxycarbonyl (Fmoc) based solid phase peptide chemistry on a 25 µmol [1,2] scale. The resin was used as such to prepare Ub-VME or functionalised on the N-terminus by coupling rhodamine. The unmodified or rhodamine-modified Ub was removed from the resin by using 1,1,1,3,3,3-hexafluoropropan-2-ol (HFIP) as described. Gly-VME (5 equiv) was coupled to the C-terminus of Ub using PyBOP (5 equiv), triethylamine (Et_3N) (10 equiv) in DCM (5 mL) and stirred for 5 h at ambient temperature. Excess Gly-VME was removed by washing the DCM solution with 1 M $KHSO_4$. The organic layer was dried with Na_2SO_4 and concentrated to dryness in vacuo. To remove the side-chain protecting groups, the residue was taken up in trifluoroacetic acid/triisopropylsilane/water (5 mL; 95:2.5:2.5) and stirred for 3 h at ambient temperature. The reaction mixture was added to a falcon tube containing ice-cold pentane/diethyl ether (1:3; 40 mL), upon which the product precipitated. The precipitate was isolated by centrifugation (1500 x g, 6 min, 4°C) and washed by three cycles of resuspension in ice-cold diethyl ether and centrifugation. Finally, the pellet was

taken up in water/acetonitrile/acetic acid (65:25:10), frozen, lyophilized and purified giving Ub-VME as white powder or RhoUbVME as a pink powder. The purity of the peptides was determined by LC-MS analysis and the crude product was purified using RP-HPLC followed by lyophilization of the appropriate fractions.

Ub-VME
Deconvoluted ESI MS^+ (amu) calculated: 8589.6$[M+H]^+$; found 8590.0 $[M+H]^+$. Rt 1.34 min; HR-MS analysis for $C_{380}H_{631}N_{105}O_{118}S$: $[M+ 7H]^{7+}$ calculated: 1228.24, found: 1227.68, $[M+ 8H]^{8+}$ calculated: 1074.84, found: 1074.35, $[M+ 9H]^{9+}$ calculated: 955.52, found: 954.98, $[M+ 10H]^{10+}$ calculated: 860.07, found: 859.68, $[M+ 11H]^{11+}$ calculated: 781.98, found: 781.62, $[M+ 12H]^{12+}$ calculated: 716.90, found: 716.57.

Rho-Ub-VME
Deconvoluted ESI MS^+ (amu) calculated: 8945.7 $[M+H]^+$; found 8943.0 $[M+H]^+$. Rt 1.44 min; HR-MS analysis for $C_{401}H_{643}N_{107}O_{122}S$: $[M+ 5H]^{5+}$ calculated: 1790.35, found: 1789.58, $[M+ 6H]^{6+}$ calculated: 1492.13, found: 1491.49, $[M+ 7H]^{7+}$ calculated: 1279.11, found: 1278.56, $[M+ 8H]^{8+}$ calculated: 1119.35, found: 1118.87, $[M+ 9H]^{9+}$ calculated: 995.09, found: 994.66, $[M+ 10H]^{10+}$ calculated: 895.68, found: 895.30.

Chemical synthesis of K27, K29 and K33-linked di-ubiquitin species
K27-, K29-, and K33-linked di-Ub was prepared using analogues methods as described in [2] in short:
The γ-thiolysine-Ub_p (StBu protected) was dissolved in DMSO (15 mg/100 μL) and added to 8 M Gdn-HCl/100 mM phosphate buffer, pH 7.6 (final concentration 7.5 mg/mL) supplemented with 100 mM TCEP and reacted at 37°C. After LC-MS analysis revealed complete deprotection of the thiolysine 1 equivalent of Ub^D-thioester dissolved in DMSO (15 mg/100 μL) and 8 M Gdn.HCl/100 mM phosphate buffer, pH 7.6 (final concentration 7.5 mg/mL) and 50 mM MPAA were added. The pH was readjusted to 7.6 and the reaction was reacted for 16 h at 37°C. RP-HPLC purification was followed by lyophilization of the appropriate fractions.
Desulfurization was achieved by dissolving in DMSO (concentration 15 mg/100 μL) and subsequent dilution into 8 M Gdn. HCl/100 mM phosphate buffer at pH 7.6 (final concentration 5 mg/mL) and 150 mM TCEP. VA044 (25 mg/mL) and GSH (25 mg/mL) were added again, the pH was adjusted to 7.0. The mixture was allowed to react for 16 hours at 37°C followed by RP-HPLC purification and lyophilization of the appropriate fractions.
The lyophilized di-Ub was dissolved in DMSO (concentration 7.5 mg/100 μL) and diluted into 20 mM Tris-HCl pH 7.6, 150 mM NaCl (7.5 mg/mL) and purified on an S75 16/600 Sephadex size exclusion column. Appropriate fractions were collected and pooled followed by spin-filtration (Amicon 3 kDa MWCO) to concentrate the sample to 5.0 mg/mL. The aliquots were snap frozen and stored at -80°C until further use.

Synthesized Di-Ub
Deconvoluted ESI MS^+ (amu) calcd: 17093.7, found 17094.4, rt: 1.36 min. HR-MS analysis for $C_{757}H_{1258}N_{210}O_{235}S$: $[M+ 10H]^{10+}$ calculated: 1710.3376, found: 1710.3276, $[M+ 11H]^{11+}$ calculated: 1554.9436, found: 1554.9438, $[M+ 12H]^{12+}$ calculated: 1425.4490, found: 1425.4441, $[M+ 13H]^{13+}$ calculated: 1315.8766, found: 1315.8804, $[M+ 14H]^{14+}$ calculated: 1221.9574, found: 1221.9567, $[M+ 15H]^{15+}$ calculated: 1140.5608, found: 1140.5573, $[M+ 16H]^{16+}$ calculated: 1069.3387, found: 1069.3364, $[M+ 17H]^{17+}$ calculated: 1006.4957, found: 1006.4905, $[M+ 18H]^{18+}$ calculated: 950.6353, found: 950.6315, $[M+ 19H]^{19+}$ calculated: 900.6549, found: 900.6536, $[M+ 20H]^{20+}$ calculated: 855.6725, found: 855.6719, $[M+ 21H]^{21+}$ calculated: 814.9742, found: 814.9700, $[M+ 22H]^{22+}$ calculated: 777.9758, found: 777.9737, $[M+ 23H]^{23+}$ calculated: 744.1946, found: 744.1901, $[M+ 24H]^{24+}$ calculated: 713.2284, found: 713.2257, $[M+ 25H]^{25+}$ calculated: 684.7396, found: 684.7383.

References

1 de Jong, A. *et al.* Ubiquitin-based probes prepared by total synthesis to profile the activity of deubiquitinating enzymes. *Chembiochem* **13**, 2251-2258, doi:10.1002/cbic.201200497 (2012).

2 El Oualid, F. *et al.* Chemical synthesis of ubiquitin, ubiquitin-based probes, and diubiquitin. *Angew Chem Int Ed Engl* **49**, 10149-10153, doi:10.1002/anie.201005995 (2010).
