# Supplementary material for: Structural snapshots along K48-linked ubiquitin chain formation by the HECT E3 UBR5
Source: Nat Chem Biol. 2023 Aug 24;20(2):190–200. doi: 10.1038/s41589-023-01414-2 (PMC10830417; doi:10.1038/s41589-023-01414-2)
Supplement: Supplementary file 2 — Reporting Summary [file 41589_2023_1414_MOESM2_ESM.pdf]

nature portfolio

Corresponding author(s): Brenda A. Schulman

Last updated by author(s): Jul 14, 2023

Reporting Summary

Nature Portfolio wishes to improve the reproducibility of the work that we publish. This form provides structure for consistency and transparency in reporting. For further information on Nature Portfolio policies, see our Editorial Policies and the Editorial Policy Checklist.

Statistics

For all statistical analyses, confirm that the following items are present in the figure legend, table legend, main text, or Methods section.

n/a	Confirmed	
☒	☐	The exact sample size (*n*) for each experimental group/condition, given as a discrete number and unit of measurement
☒	☐	A statement on whether measurements were taken from distinct samples or whether the same sample was measured repeatedly
☒	☐	The statistical test(s) used AND whether they are one- or two-sided<br>*Only common tests should be described solely by name; describe more complex techniques in the Methods section.*
☒	☐	A description of all covariates tested
☒	☐	A description of any assumptions or corrections, such as tests of normality and adjustment for multiple comparisons
☒	☐	A full description of the statistical parameters including central tendency (e.g. means) or other basic estimates (e.g. regression coefficient) AND variation (e.g. standard deviation) or associated estimates of uncertainty (e.g. confidence intervals)
☒	☐	For null hypothesis testing, the test statistic (e.g. *F*, *t*, *r*) with confidence intervals, effect sizes, degrees of freedom and *P* value noted<br>*Give P values as exact values whenever suitable.*
☒	☐	For Bayesian analysis, information on the choice of priors and Markov chain Monte Carlo settings
☒	☐	For hierarchical and complex designs, identification of the appropriate level for tests and full reporting of outcomes
☒	☐	Estimates of effect sizes (e.g. Cohen's *d*, Pearson's *r*), indicating how they were calculated

Our web collection on statistics for biologists contains articles on many of the points above.

Software and code

Policy information about availability of computer code

Data collection	Gel imaging: Amersham Imager 600, Amersham Typhoon; Cryo-EM: SerialEM v3.8.0-b5, FEI EPU v2.7.0
Data analysis	Data processing: RELION v3.1.1, RELION v4.0, CryoSparc v4.2.0, Gautomatch v0.56, CTFFIND v4.1;<br>Structure Analysis and Visualization: UCSF Chimera v1.15, ChimeraX v1.4, PyMol v2.3.4;<br>Model Building: AlphaFold2, Alphafold 2.2 multimer, COOT v0.9.6, Phenix.refine v1.19.2-4158, DeepEMhancer (https://github.com/rsanchezgarc/deepEMhancer)<br>Others: Waters MassLynx Mass Spectrometry Software 4.1, Refeyn AcquireMP 2.3.0, Refeyn DiscoverMP 2.3.0, MolProbity v.4.2, ChemDraw v22.2.0

For manuscripts utilizing custom algorithms or software that are central to the research but not yet described in published literature, software must be made available to editors and reviewers. We strongly encourage code deposition in a community repository (e.g. GitHub). See the Nature Portfolio guidelines for submitting code & software for further information.

Data

Policy information about availability of data

All manuscripts must include a data availability statement. This statement should provide the following information, where applicable:

- Accession codes, unique identifiers, or web links for publicly available datasets
- A description of any restrictions on data availability
- For clinical datasets or third party data, please ensure that the statement adheres to our policy

Data availability statement
The structural data will be available from EMDB and RCSB upon manuscript publication (UBR5Dimer EMDB-16355, PDB 8C06; UBR5Dimer~UbD~UbA EMDB-16356, PDB 8C07. Cryo-EM maps were deposited and are available via the following accession number: UBR5C2768A EMDB-16865, UBE2D2~UbD~UBR5Dimer EMDB-16867, UBR5~UbD EMDB-16866, UBR5Dimer~UbD~UbA (global map) EMDB-17466). Raw gel images are provided as source data. Coomassie-stained SDS-PAGE of all assays are added as Supplementary Figures 6 and 7. Accession codes of other (published) data that was used for comparison: PDB: 4LCD, PDB: 3JVZ, PDB: 2QHO, PDB: 6XZ1, PDB: 3NY3, PDB: 4BBN, EMD-28646. There is no restriction on data availability.

Human research participants

Policy information about studies involving human research participants and Sex and Gender in Research.

Reporting on sex and gender	No research involving human participants has been performed
Population characteristics	No research involving human participants has been performed
Recruitment	No research involving human participants has been performed
Ethics oversight	No research involving human participants has been performed

Note that full information on the approval of the study protocol must also be provided in the manuscript.

Field-specific reporting

Please select the one below that is the best fit for your research. If you are not sure, read the appropriate sections before making your selection.

☒ Life sciences ☐ Behavioural & social sciences ☐ Ecological, evolutionary & environmental sciences

For a reference copy of the document with all sections, see nature.com/documents/nr-reporting-summary-flat.pdf

Life sciences study design

All studies must disclose on these points even when the disclosure is negative.

Sample size	Calculations to predetermine sample size were not performed. Sample sizes were chosen to facilitate clear and reliable interpretation of the results. The rationale of the sample size was based on previous experience with these experiments in terms of variability.
Data exclusions	No data were excluded.
Replication	All experiments were repeated at least twice, but mostly three or more times with several controls included. All replication-attempts were succesful.
Randomization	No grouping of samples was performed
Blinding	No grouping of samples was performed

Reporting for specific materials, systems and methods

We require information from authors about some types of materials, experimental systems and methods used in many studies. Here, indicate whether each material, system or method listed is relevant to your study. If you are not sure if a list item applies to your research, read the appropriate section before selecting a response.

Materials & experimental systems

n/a	Involved in the study
☒	☐ Antibodies
☐	☒ Eukaryotic cell lines
☒	☐ Palaeontology and archaeology
☒	☐ Animals and other organisms
☒	☐ Clinical data
☒	☐ Dual use research of concern

Methods

n/a	Involved in the study
☒	☐ ChIP-seq
☒	☐ Flow cytometry
☒	☐ MRI-based neuroimaging

Eukaryotic cell lines

Policy information about cell lines and Sex and Gender in Research

Cell line source(s)	HEK293S GnTI- were obtained from ATCC (identifier: CRL-3022), Sf9 were obtained from Thermo Fischer (identifier: 11496015), Hi5 cells (BTI-TN-5B1-4) were obtained from ThermoFisher Scientific (catalogue number: B85502).
Authentication	Cell lines were not authenticated.
Mycoplasma contamination	Cell lines were tested regularly for mycoplasm contamination and always were mycoplasm-negative.
Commonly misidentified lines (See ICLAC register)	No commonly misidentified cell lines were used in this study.
