# Supplementary figures and images for: Structural snapshots along K48-linked ubiquitin chain formation by the HECT E3 UBR5
Source: Nat Chem Biol. 2023 Aug 24;20(2):190–200. doi: 10.1038/s41589-023-01414-2 (PMC10830417; doi:10.1038/s41589-023-01414-2)

Figure 1b

Molecular weight marker for Figure 1b

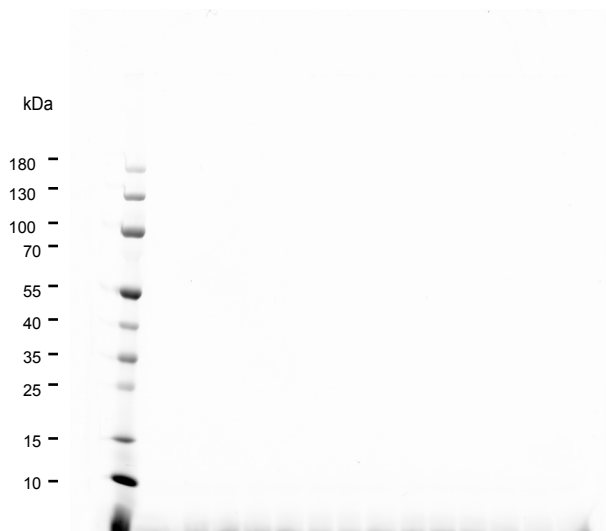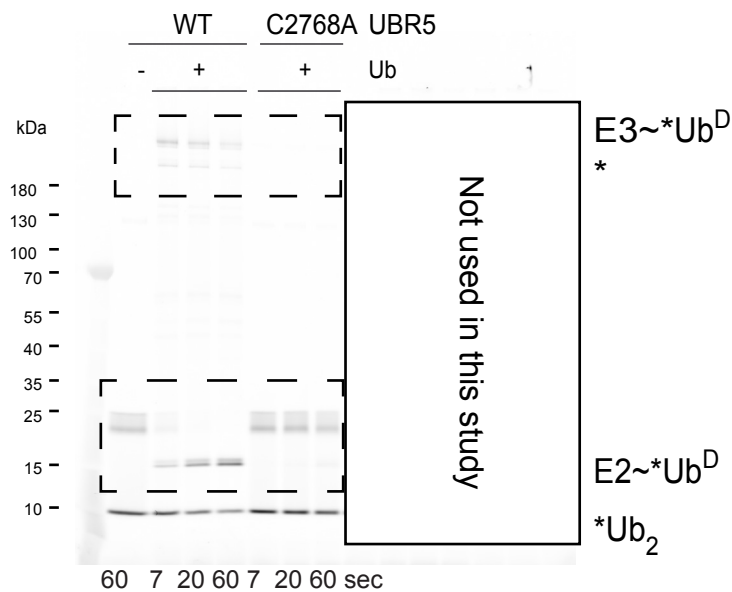

Figure 1i

Molecular weight marker for Figure 1i

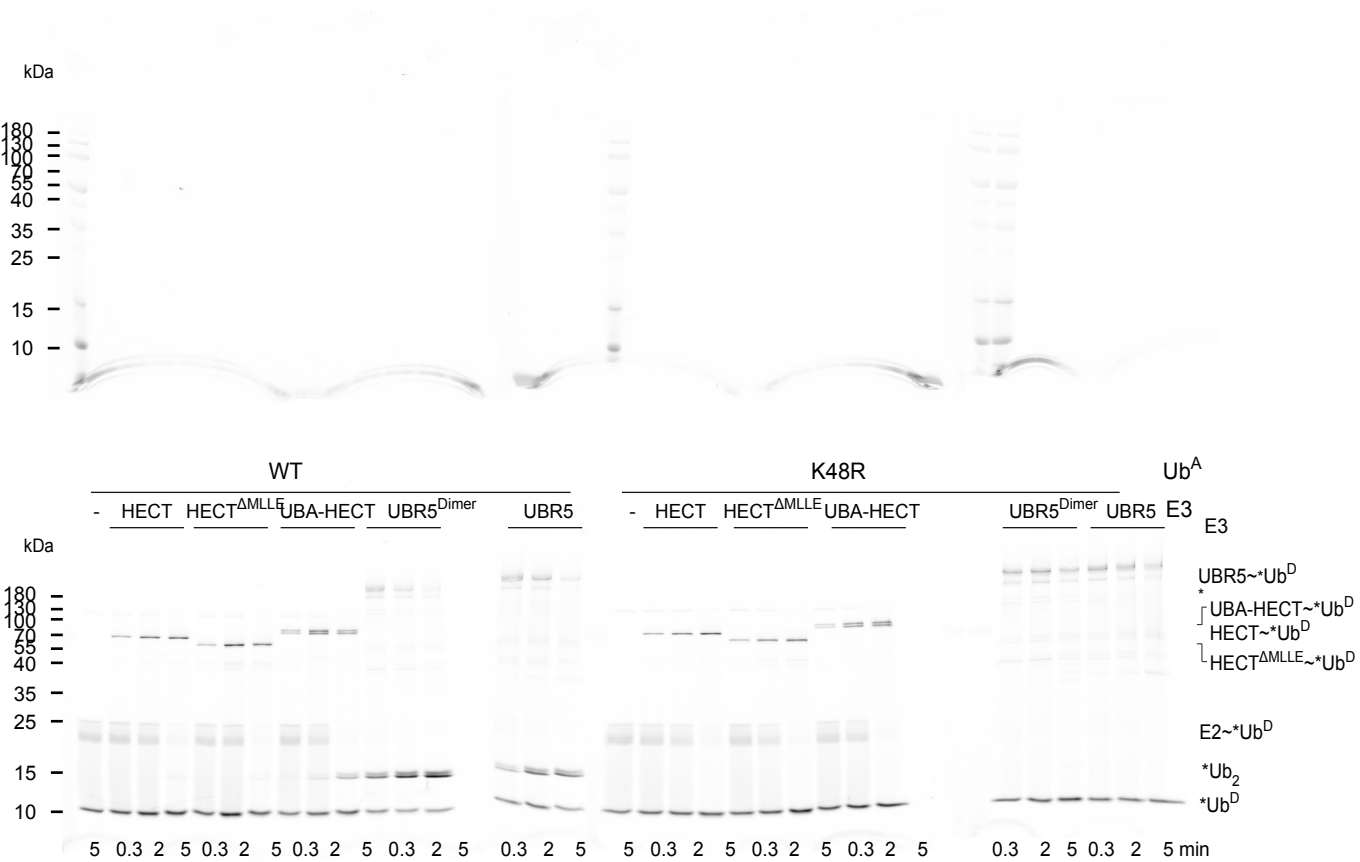

Supplement: Supplementary file 6 — Unprocessed, uncropped SDS–PAGE. [file 41589_2023_1414_MOESM6_ESM.pdf]
