# Supplementary material for: Structural snapshots along K48-linked ubiquitin chain formation by the HECT E3 UBR5
Source: Nat Chem Biol. 2023 Aug 24;20(2):190–200. doi: 10.1038/s41589-023-01414-2 (PMC10830417; doi:10.1038/s41589-023-01414-2)

Figure 2d

Molecular weight marker for Figure 2d, non-reducing SDS-PAGE

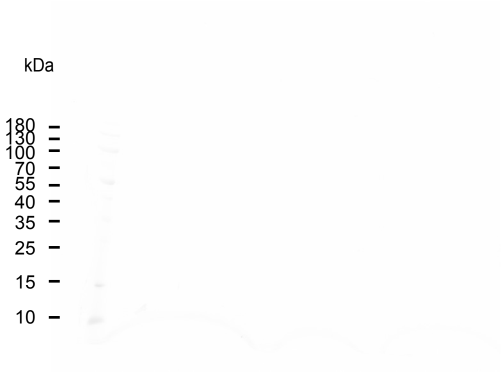

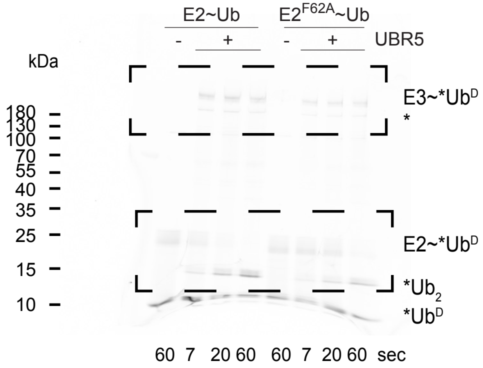

Non-reducing

Molecular weight marker for Figure 2d, reducing SDS-PAGE

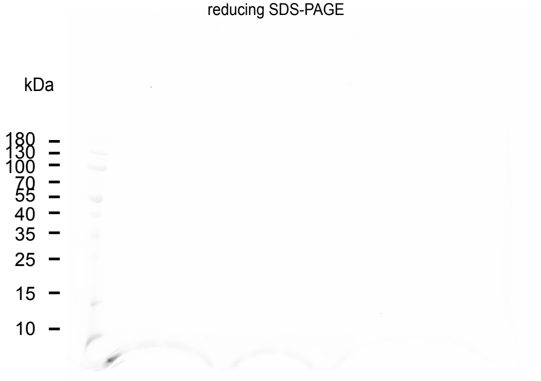

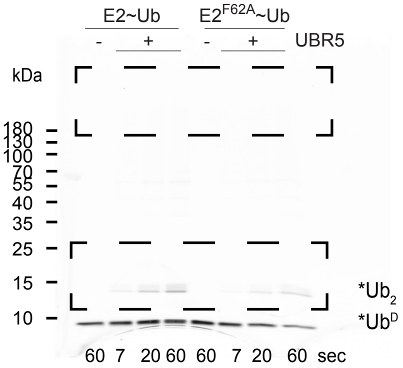

Reducing

Figure 2e

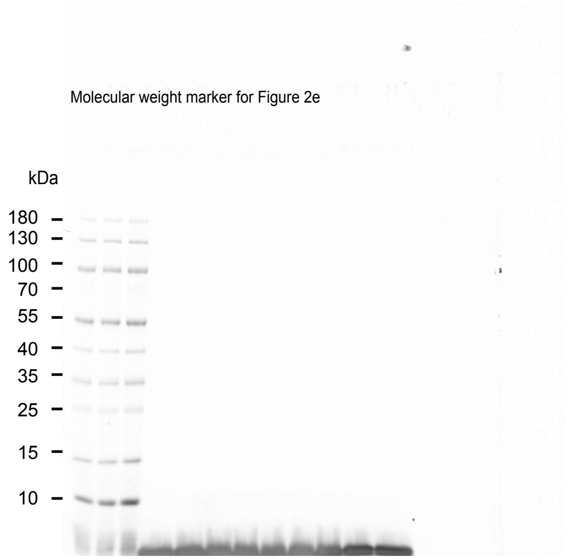
Molecular weight marker for Figure 2e
kDa
180
130
100
70
55
40
35
25
15
10

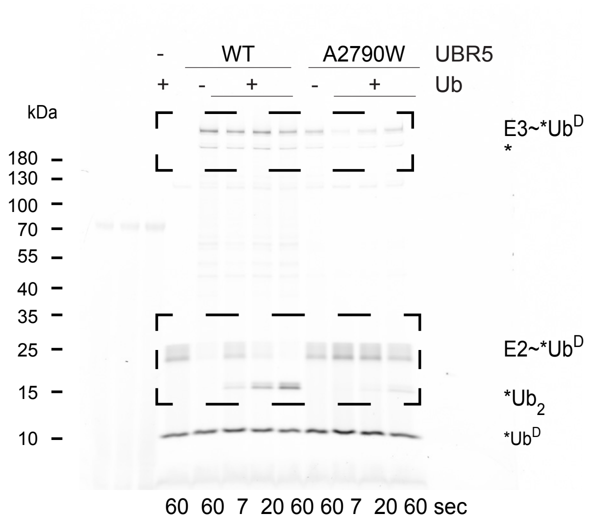
- WT A2790W UBR5
+ - + - + Ub
kDa
180
130
100
70
55
40
35
25
15
10
E3~*Ub^D
*
E2~*Ub^D
*Ub2
*Ub^D
60 60 7 20 60 60 7 20 60 sec

Figure 2i

Molecular weight marker for Figure 2i, left SDS-PAGE

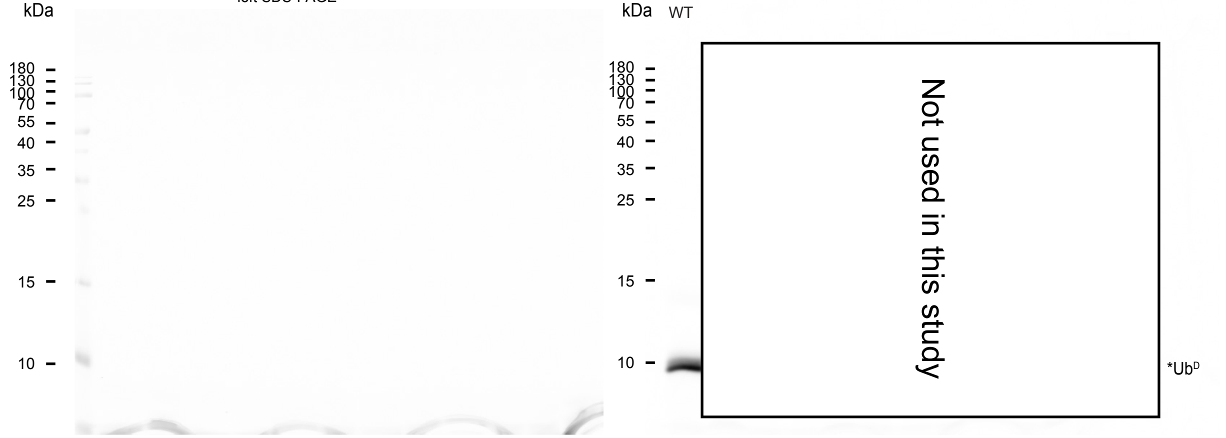

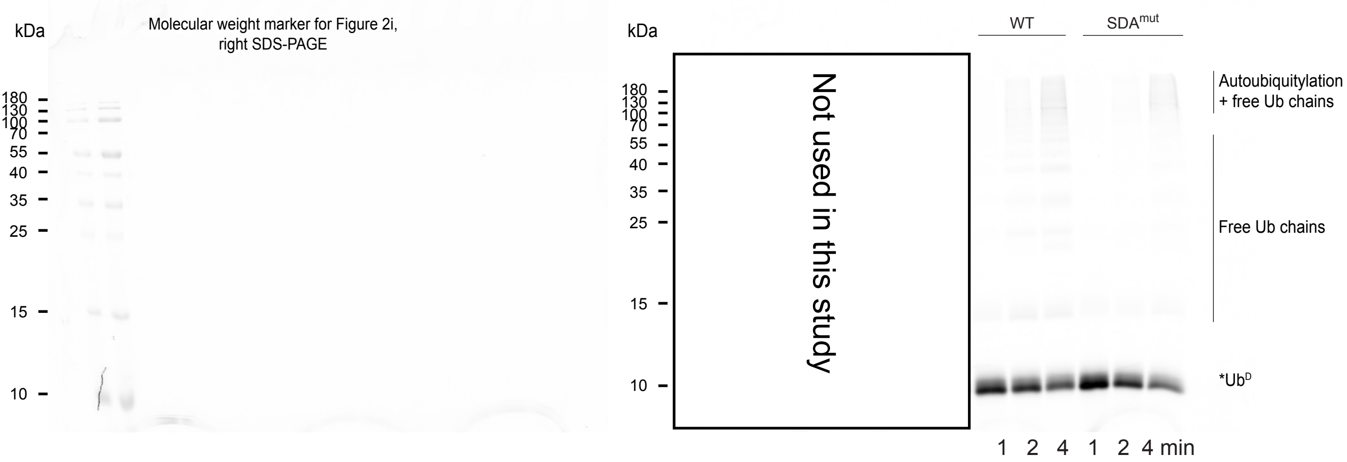

Supplement: Supplementary file 7 — Unprocessed, uncropped SDS–PAGE. [file 41589_2023_1414_MOESM7_ESM.pdf]
