# Supplementary material for: Structural snapshots along K48-linked ubiquitin chain formation by the HECT E3 UBR5
Source: Nat Chem Biol. 2023 Aug 24;20(2):190–200. doi: 10.1038/s41589-023-01414-2 (PMC10830417; doi:10.1038/s41589-023-01414-2)
Supplement: Supplementary file 8 — Unprocessed, uncropped SDS–PAGE. [file 41589_2023_1414_MOESM8_ESM.pdf]

Figure 5c, left panel

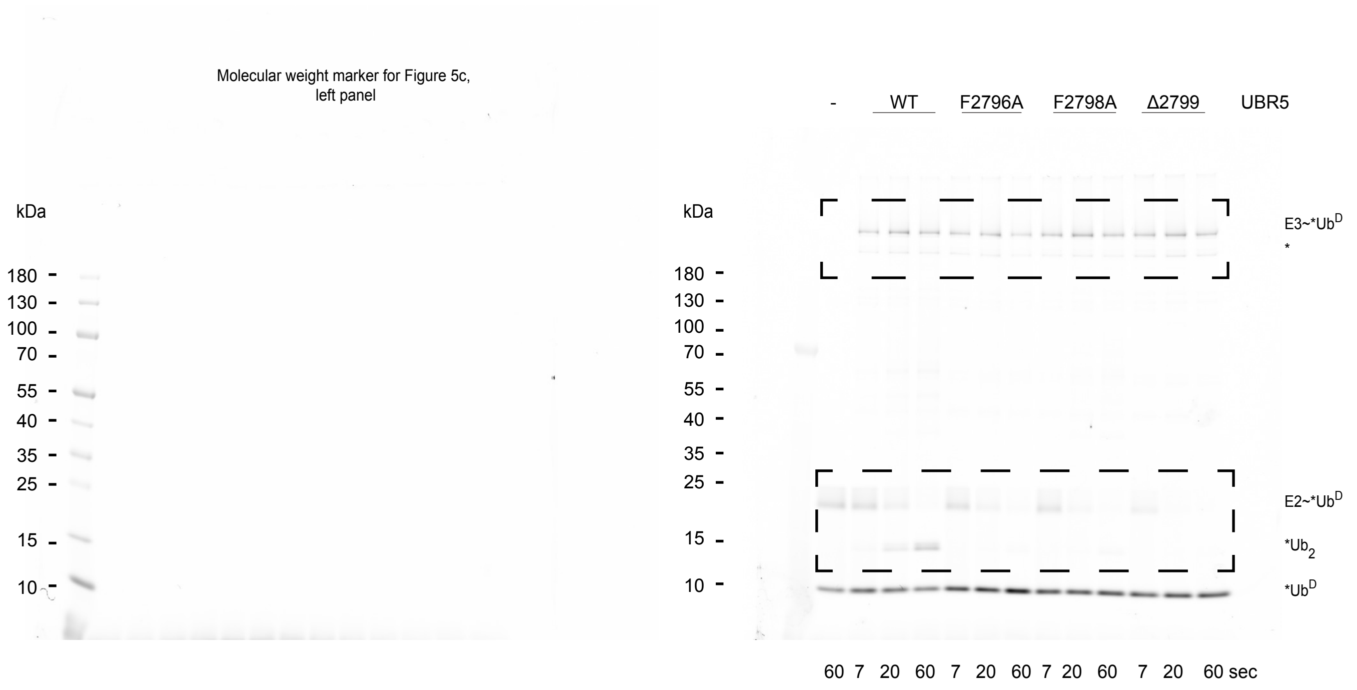

Figure 5c, right panel

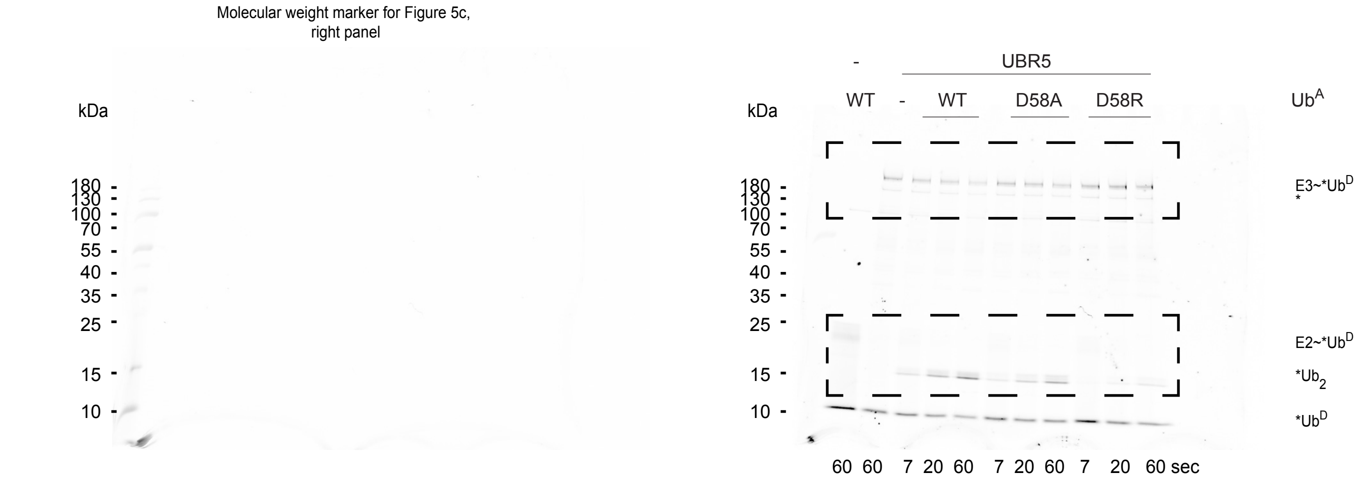

Figure 5d, left panel

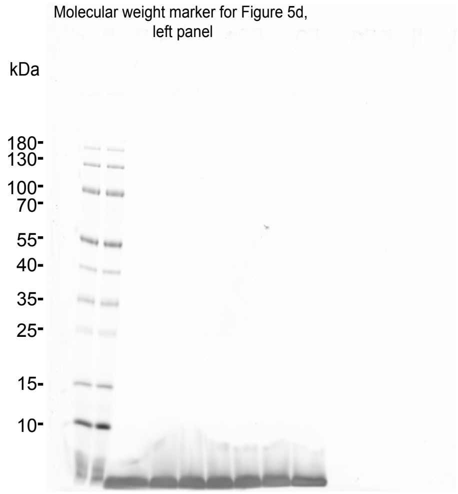

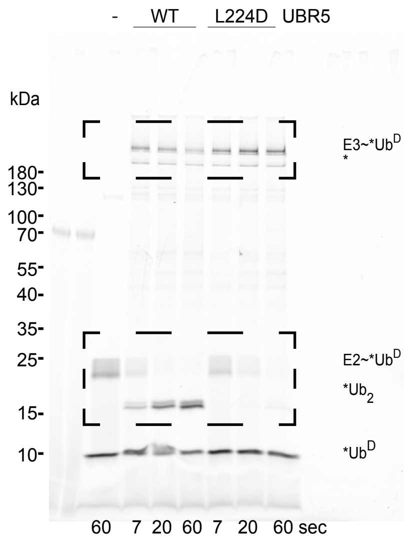

Figure 5d, right panel

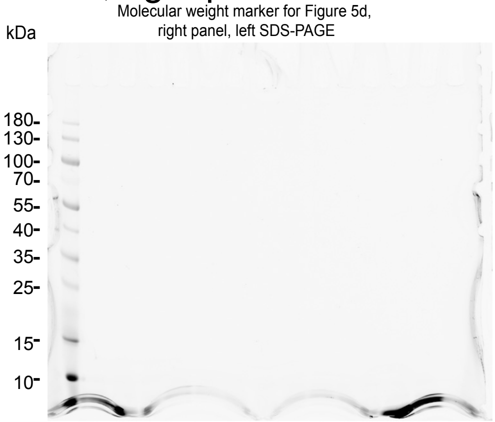

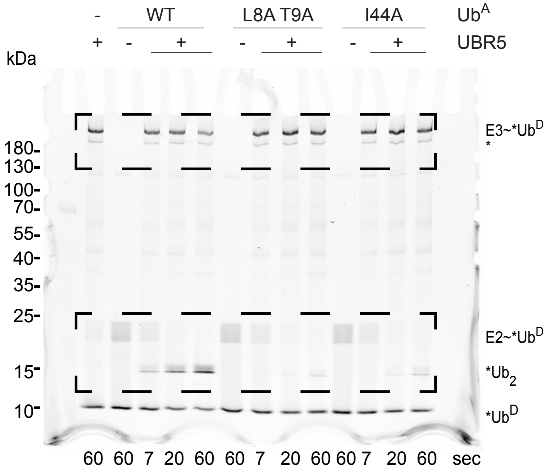

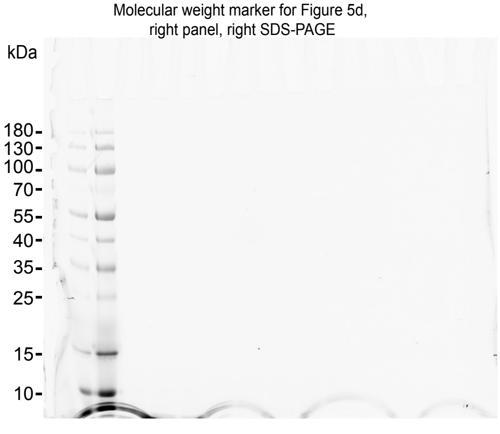

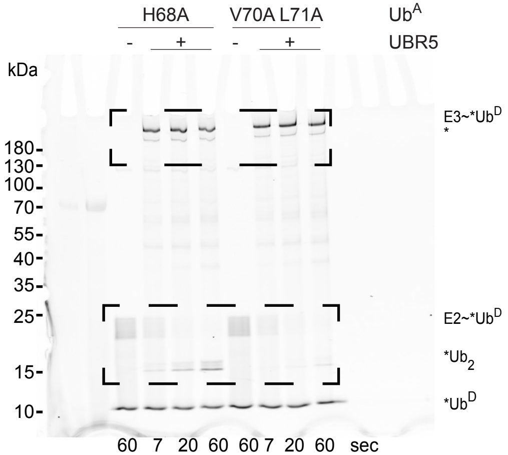

Figure 5e

Molecular weight marker for Figure 5e,
left SDS-PAGE

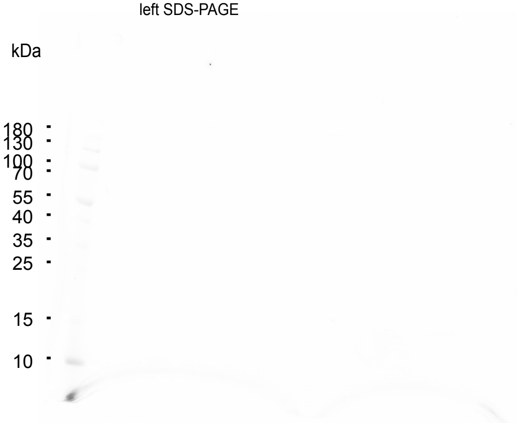

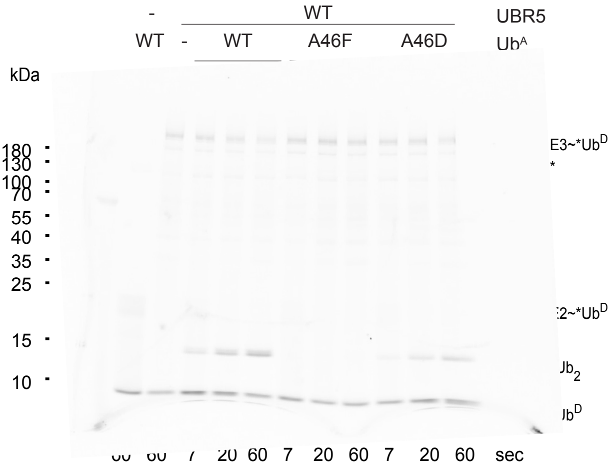

Molecular weight marker for Figure 5e,
right SDS-PAGE

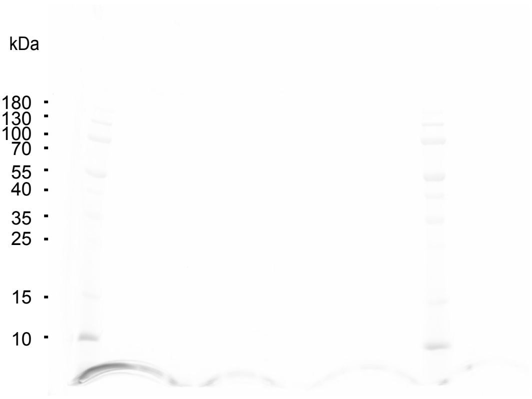

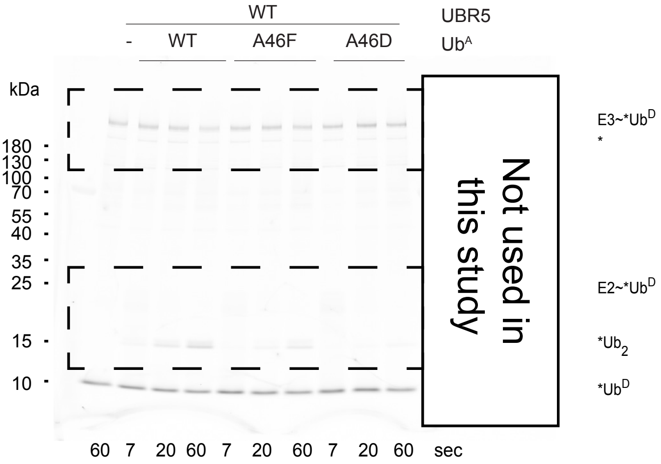

Figure 5f

Molecular weight marker for Figure 5f, left SDS-PAGE

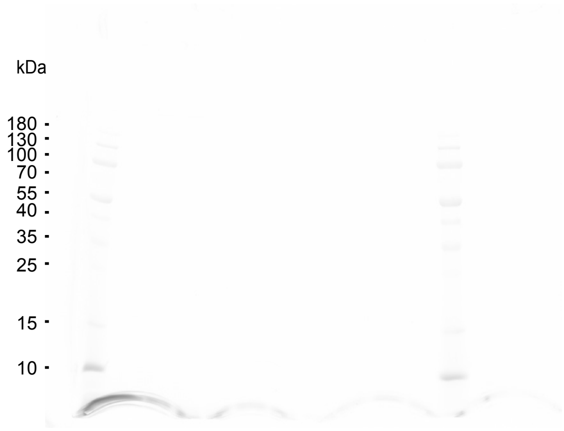

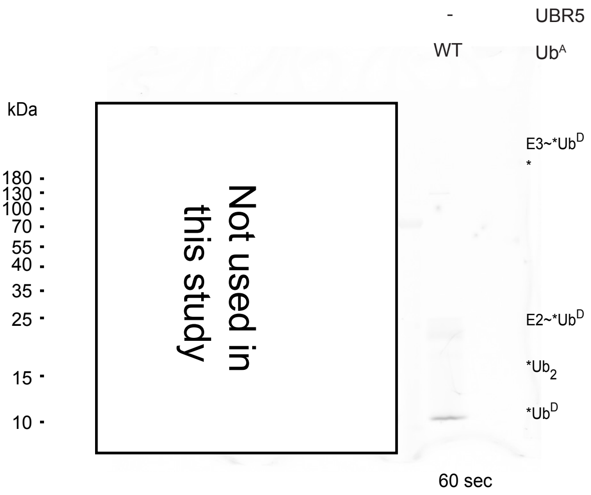

Molecular weight marker for Figure 5f, right SDS-PAGE

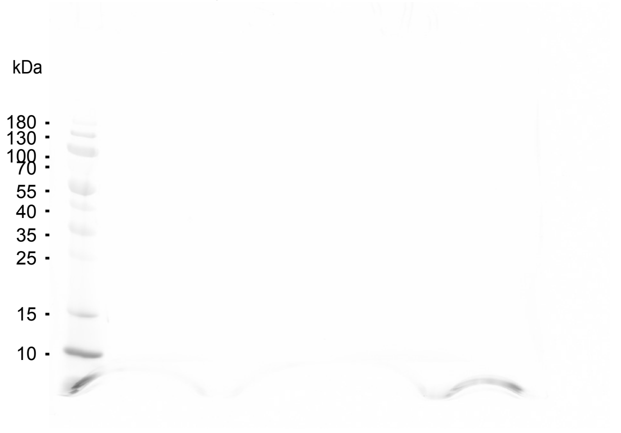

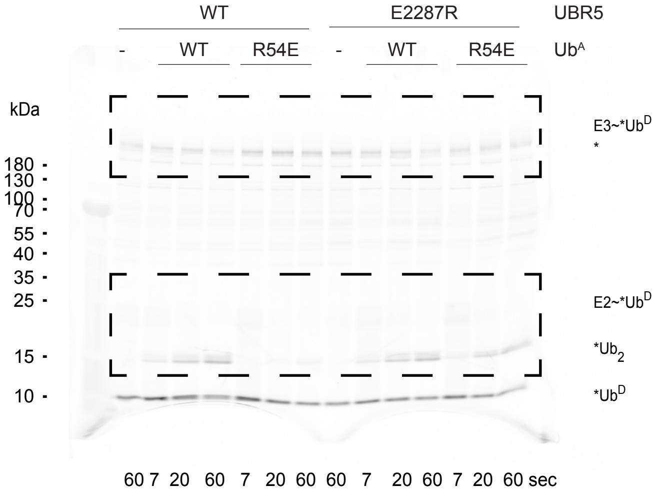

Figure 5g

Molecular weight marker for Figure 5g

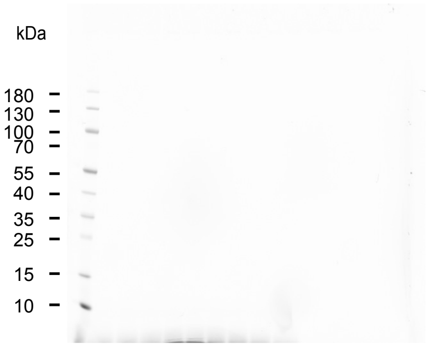

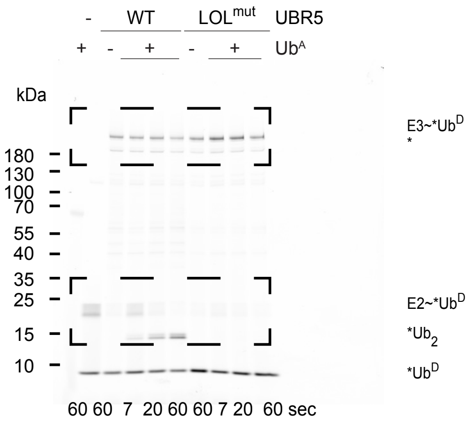

Figure 5h

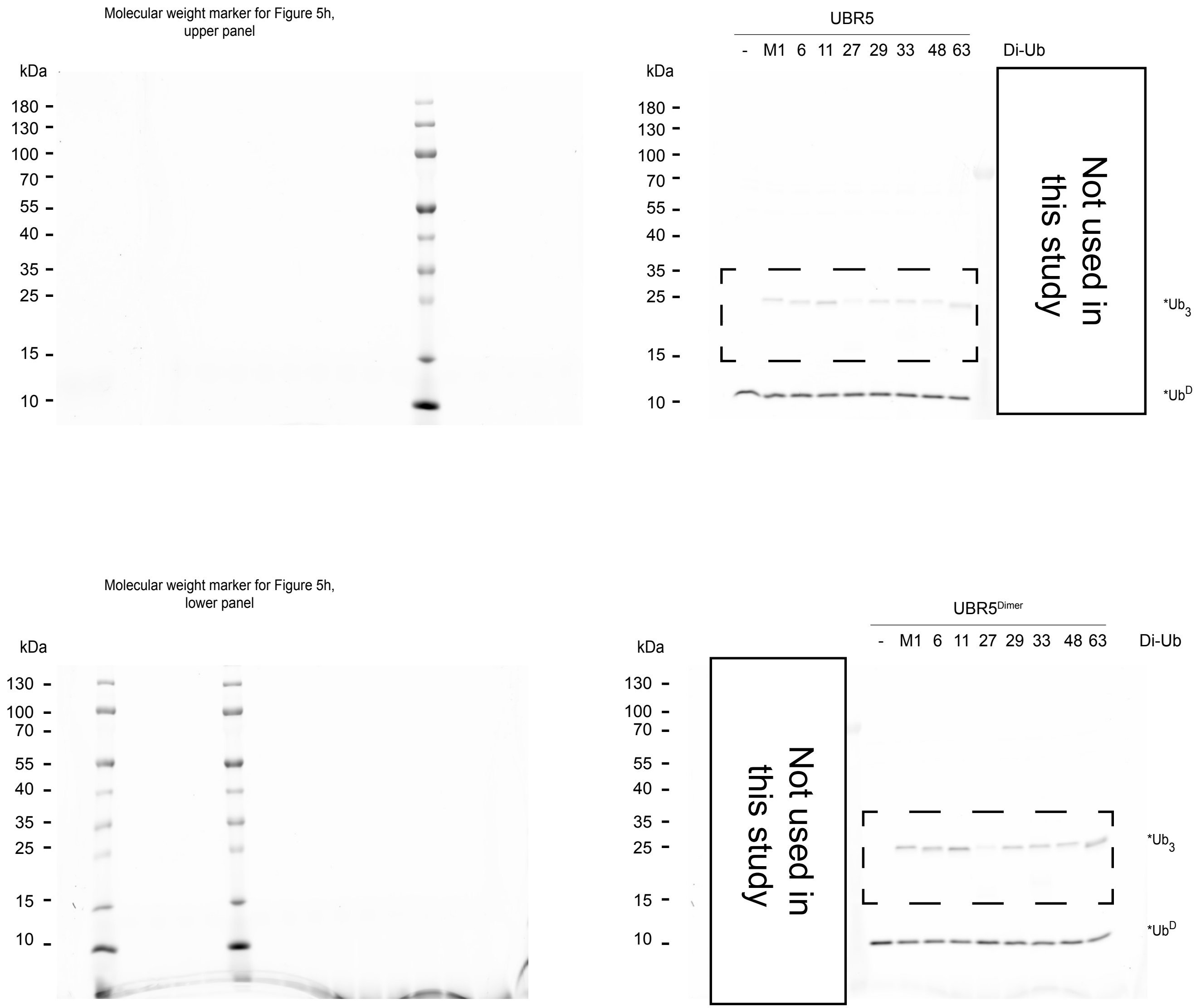
Molecular weight marker for Figure 5h, upper panel
kDa
180
130
100
70
55
40
35
25
15
10
UBR5
- M1 6 11 27 29 33 48 63
Di-Ub
Not used in this study
*Ub3
*UbD
Molecular weight marker for Figure 5h, lower panel
kDa
130
100
70
55
40
35
25
15
10
UBR5Dimer
- M1 6 11 27 29 33 48 63
Di-Ub
Not used in this study
*Ub3
*UbD
