# Supplementary material for: Structural snapshots along K48-linked ubiquitin chain formation by the HECT E3 UBR5
Source: Nat Chem Biol. 2023 Aug 24;20(2):190–200. doi: 10.1038/s41589-023-01414-2 (PMC10830417; doi:10.1038/s41589-023-01414-2)

# Extended Data Figure 1a

Molecular weight marker for Extended Data Figure 1a,
non-reducing PAGE

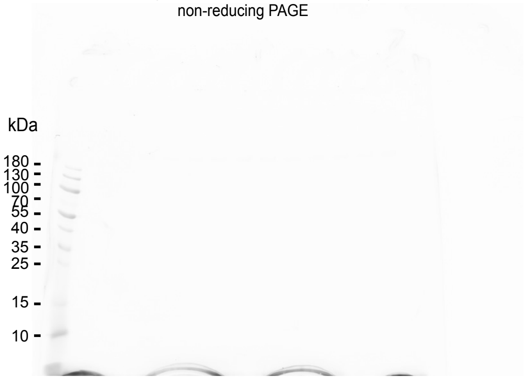


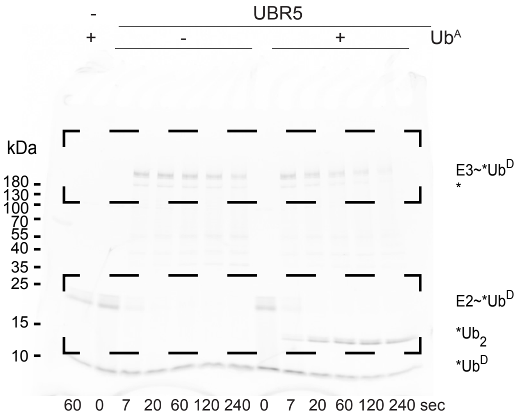


Non-reducing SDS-PAGE

Molecular weight marker for Extended Data Figure 1a,
reducing PAGE

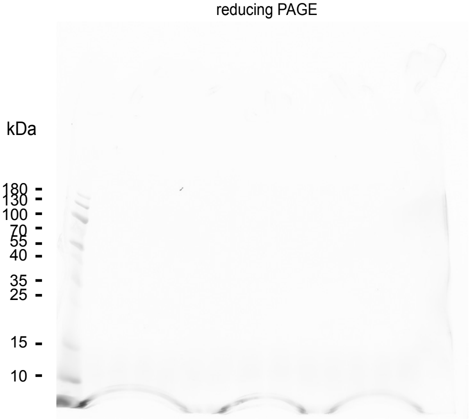


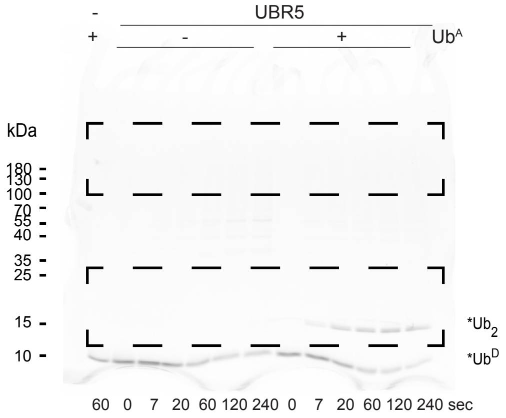


Reducing SDS-PAGE

# Extended Data Figure 1f

Molecular weight marker for Extended Data Figure 1f, upper panel

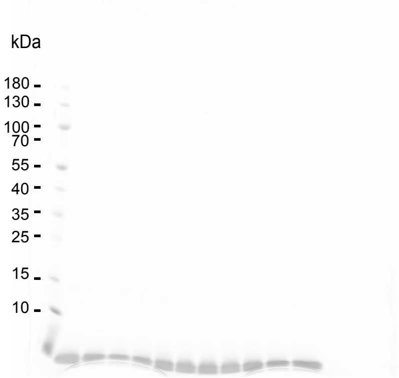


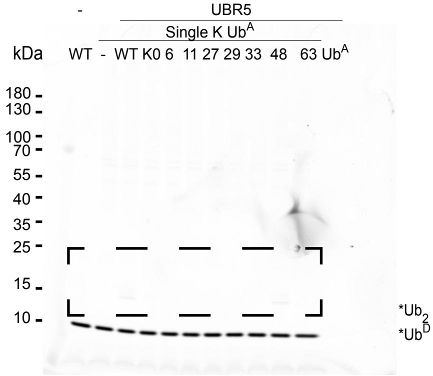


Molecular weight marker for Extended Data Figure 1f, lower panel

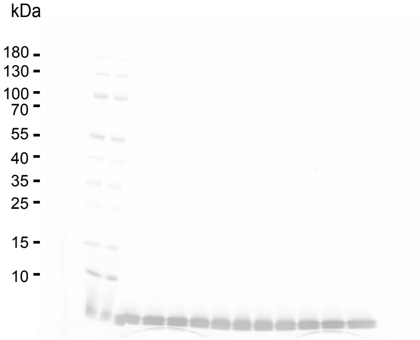


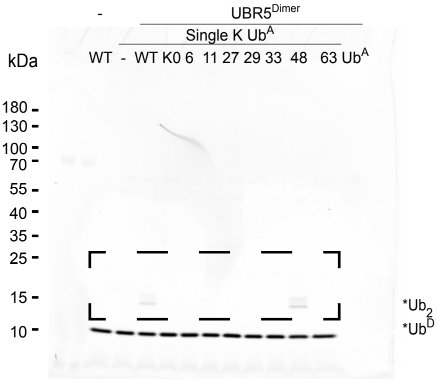

# Extended Data Figure 1g

Molecular weight marker for Extended Data Figure 1g,

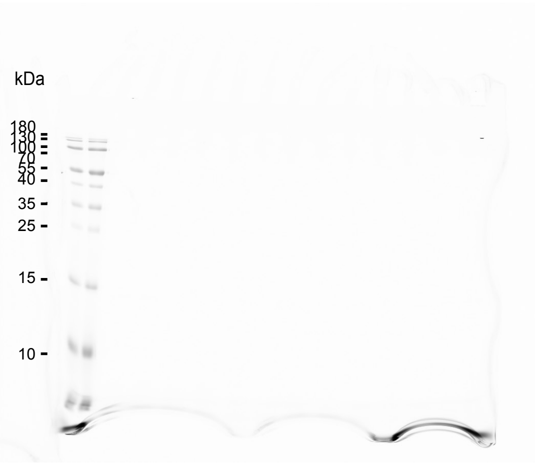


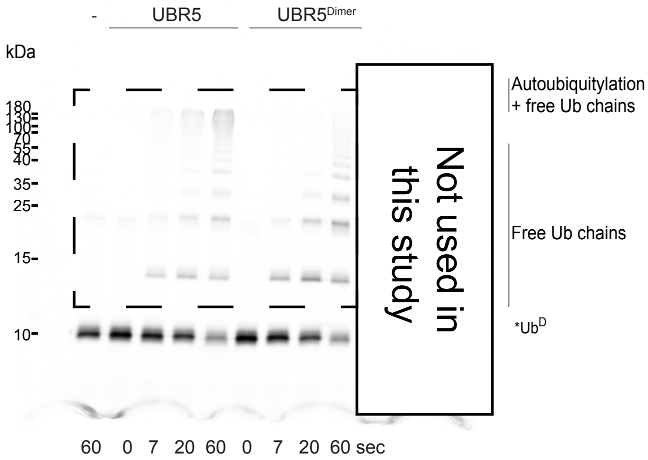

Supplement: Supplementary file 9 — Unprocessed, uncropped SDS–PAGE. [file 41589_2023_1414_MOESM9_ESM.pdf]
