# Supplementary material for: Structural snapshots along K48-linked ubiquitin chain formation by the HECT E3 UBR5
Source: Nat Chem Biol. 2023 Aug 24;20(2):190–200. doi: 10.1038/s41589-023-01414-2 (PMC10830417; doi:10.1038/s41589-023-01414-2)

Extended Data Figure 4d

Molecular weight marker for Extended Data Figure 4d, left SDS-PAGE

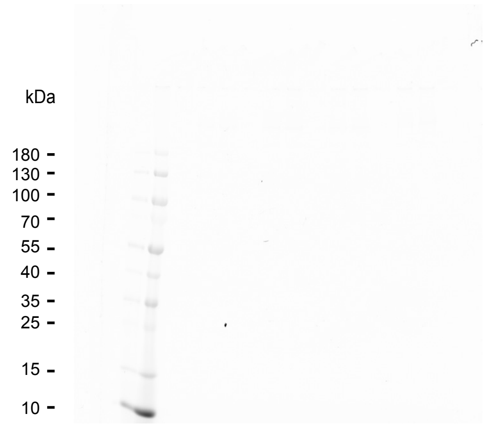

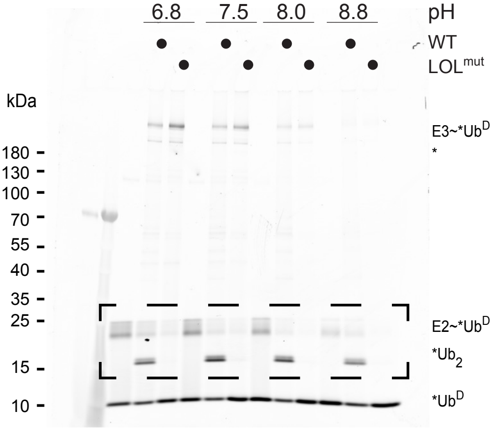

Molecular weight marker for Extended Data Figure 4d, right SDS-PAGE

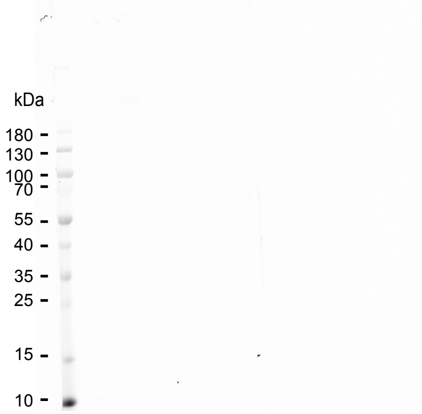

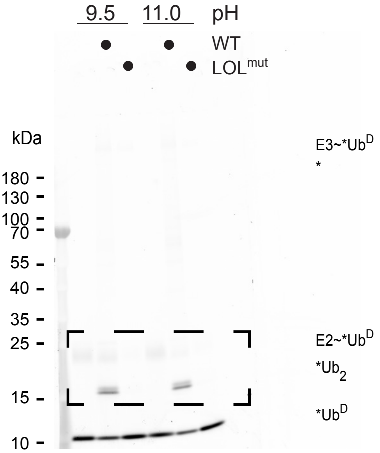

Supplement: Supplementary file 11 — Unprocessed, uncropped SDS–PAGE. [file 41589_2023_1414_MOESM11_ESM.pdf]
